# Supplementary material for: The Influence of Regiochemistry on the Performance of Organic Mixed Ionic and Electronic Conductors
Source: Angew Chem Weinheim Bergstr Ger. 2023 Jun 12;135(29):e202304390. doi: 10.1002/ange.202304390 (PMC10962556; doi:10.1002/ange.202304390)
Supplement: Supplementary file 1 — Supporting Information [file ANGE-135-0-s001.pdf]

Angewandte Chemie
Eine Zeitschrift der Gesellschaft Deutscher Chemiker

Supporting Information

The Influence of Regiochemistry on the Performance of Organic Mixed Ionic and Electronic Conductors

R. Halaksa, J. H. Kim, K. J. Thorley, P. A. Gilhooly-Finn, H. Ahn, A. Savva, M.-H. Yoon, C. B. Nielsen**

Supporting Information

Table of contents

1 General information 2
2 Synthesis 3
3 NMR spectra 14
4 Gel permeation chromatography 27
5 Thermogravimetric analysis 28
6 Differential scanning calorimetry 32
7 Cyclic voltammetry 35
8 Spectroelectrochemistry 50
9 OECT fabrication 56
10 OECT performance 57
11 Electrochemical impedance spectroscopy 57
12 Density Functional Theory calculations 59
13 Grazing-Incidence Wide-Angle X-ray Scattering 60
14 Electrochemical quartz crystal microbalance 64
References 66

1 General information

Chemicals were purchased from Acros Organics, Fluorochem, and TCI and were used without further purification. Solvents are HPLC grade and were purchased from Honeywell and used without further purification. 3-(2-(2-(2-methoxyethoxy)ethoxy)ethoxy)thiophene (4) was prepared using a previously reported procedure with a yield of 81 %.[1] Triisopropyl(3-(2-(2-(2-methoxyethoxy)ethoxy)ethoxy)-thiophen-2-yl)silane (5) was prepared using a previously reported procedure with a yield of 89 %.[2]

^{1}H and ^{13}C NMR spectroscopy were carried out on Bruker AV400 or Bruker AVIII400 spectrometers. Chemical shifts (δ) are quoted in ppm relative to the residual solvent peak. d-$CHCl_3$ was purchased from Cambridge isotopes and d_2-TCE was purchased from Fluorochem.

HRMS was carried out on Synapt G2-Si High-Definition Mass Spectrometer. MALDI-TOF was carried out on a Bruker Autoflex Maldi-TOF. TGA was carried out on a TA instruments Q500. DSC was carried out on a Perkin Elmer DSC4000. GPC was carried out using Shimadzu Prominence GPC system, comprised of a SIL-20A auto sampler, LC-20AT liquid chromatograph, CTO-20A column oven, RID-20A refractive index detector and an SPD-20A UV-Vis detector. HPLC grade chlorobenzene was purchased from Acros Organics. Analysis was carried out on Shimadzu's LabSolutions software. UV-Vis spectroscopy and spectroelectrochemistry were carried out using a Shimadzu UV3600 UV-vis-nIR spectrometer. Thin films for UV-Vis were prepared using a Laurell Technologies WS-650Mz-23NPPB spin coater (RPM = 1000, time = 30 s, acceleration = 400). Cyclic voltammetry was carried out using a PalmSens 3 electrochemical cell on glassy carbon electrode with Ag/Ag^{+} reference electrode and platinum wire counter electrode.

GIWAXS measurements were conducted at the 9A U-SAXS beamline of the Pohang Light Source (PLS), Republic of Korea. The wavelength of X-rays was 1.12370 c5 (E = 11.025 keV), and the incidence angle of the beam light was ~0.1°. The images from GIWAXS were obtained with a 2D FT-CCD module of Rayonix MX170-HS, while the sample-to-detector distance was adjusted to be 225 mm. All polymer films were fabricated by spin-casting of polymer solution (5 mg/mL) on indium tin oxide (ITO)-coated glass substrates. Working electrodes were prepared by clipping the polymer-coated ITO glass with an alligator clip to make electrical wiring after partial removal of polymer film to expose ITO surface; the exposed ITO surface and alligator clip was not contacted with electrolyte during applying potential to the polymer films. Using the polymer-coated ITO glass working electrodes, an Ag/AgCl reference electrode, and a Pt counter electrode, electrochemical potential (V_{offset}) is applied to the polymer films using PGSTAT304N (Metrohm Autolab, the Netherlands); 0.1 M NaCl aqueous solution was used as an electrolyte after 30 min N_2 bubbling. In the case of oxidized films, after 10 s of oxidation by applying 0.8 V, the polymer films were taken out of the electrolyte and washed with deionized water and dried by N_2 blowing. The restored films were prepared by applying 0.8 V for 10 s and 0 V for 10 s, successively, and then the films were washed and dried by N_2 blowing.

All calculations were run using Gaussian 16 Rev A.03 (M. J. Frisch et al., Gaussian 16, Revision A.03. Gaussian, Inc., Wallingford CT, 2016.). Torsional PES were calculated using B3LYP-D3/6-31G* in the gas phase, with all internal co-ordinates angle allowed to relax except the fixed dihedral for each data point. Polymer fragments were built using the dihedral energetic minima found from the torsional PES, and the geometry re-optimized in the gas phase using ωB97XD/6-31G* with an ω value of 0.1 which is close to the optimal value for conjugated polymers. The ω value was then optimized to best satisfy Koopmans' theorem, as per our previous work (M. Moser et al., J. Mater. Chem. C, 2019, 7, 5359). All further calculations used this optimized ω value, with the addition of solvation using a self-consistent reaction field polarisable continuum for chloroform.

2 Synthesis

1,4-dibromo-2,5-bis[2-[2-(2-methoxyethoxy)ethoxy]ethoxy]benzene (3)

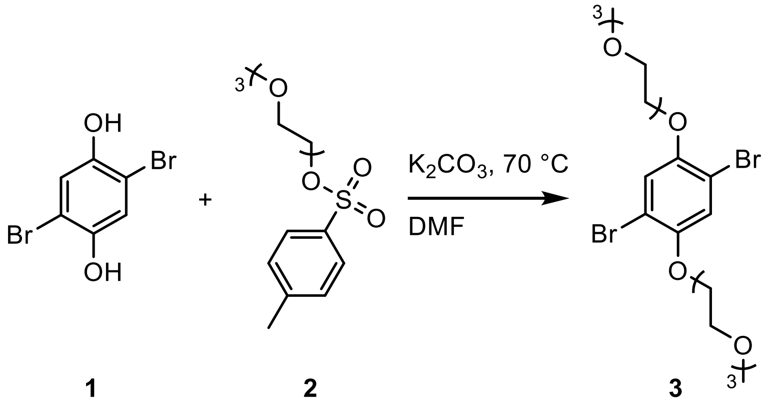

1 **2** **3**

2,5-Dibromohydroquinone (3.18 g, 19.0 mmol) and 2-(2-(2-methoxyethoxy)ethoxy)ethyl benzenesulfonate (5.78 g, 41.8 mmol) were dissolved in anhydrous DMF (150 ml). Then potassium carbonate (5.78 g, 41.8 mmol) was added at RT and temperature was raised to 70 °C and reaction mixture was stirred for 30 hours. Reaction mixture was poured into mixture of water and saturated aqueous ammonium chloride solution (300 ml) (1/1) and extracted with diethyl ether (4x100 ml). Combined organic extracts were washed with water (3x100 ml), brine (100 ml), dried ($MgSO_4$) and evaporated. Obtained crude product was dissolved in mixture of hexane and ethyl-acetate (1/1) and filtered through silica pad and evaporated. Crude product was dissolved in ethanol (40 ml) and solution of potassium hydroxide (3.54 g. 63.1 mmol) dissolved in water (40 ml) was added and reaction mixture was stirred at 70 °C overnight. Reaction mixture was poured into water (100 ml) and extracted with diethyl ether (3x100 ml). Combined organic extracts were washed with water (3x100), brine (2x100 ml), dried ($MgSO_4$) and evaporated to obtain product as a light orange oil (4.87 g, 45.7 %). 1H NMR (400 MHz, CDCl3) δ 7.14 (s, 2H), 4.14 – 4.10 (m, 4H), 3.88 – 3.85 (m, 4H), 3.78 – 3.75 (m, 4H), 3.70 – 3.63 (m, 8H), 3.57 – 3.52 (m, 4H), 3.37 (s, 6H).

(3-(2-(2-(2-methoxyethoxy)ethoxy)ethoxy)thiophene-2,5-diyl)bis(triisopropylsilane) (6)

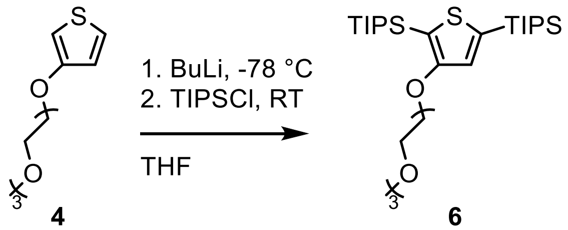

4 **6**

Compound **4** (3.77 g, 15.3 mmol) was dissolved in anhydrous THF (75 ml) in nitrogen atmosphere and reaction mixture was cooled down to -78 °C. Butyllithium (12.9 ml, 32.1 mmol, 2.5M solution in hexanes) was added dropwise and reaction mixture was allowed to stir at -78 °C one hour. Then triisopropylsilyl chloride (6.49 g, 33.7 mmol) was added and reaction was warmed up to room temperature over 2 hours. Reaction was then poured into water (150 ml) and extracted with EtAc (3x70 ml). Combined organic extract were washed with water (3x70 ml), brine (2x70 ml), dried with anhydrous $MgSO_4$ and evaporated. Silica column chromatography of crude product using hexane/EtAc (6/1) provided title compound as light-yellow oil (6.16 g, 11.1 mmol, 72 %). ^1H NMR (400 MHz, $CDCl_3$) δ 7.00 (s, 1H), 4.15 – 4.08 (m, 2H), 3.77 (t, *J* = 5.3 Hz, 2H), 3.70 – 3.67 (m, 2H), 3.65 (ddd, *J* = 7.6, 4.0,

1.7 Hz, 4H), 3.56 – 3.52 (m, 2H), 3.37 (s, 3H), 1.44 – 1.24 (m, 6H), 1.08 (dd, J = 7.5, 5.0 Hz, 36H). ^{13}C NMR (101 MHz, $CDCl_3$) δ 164.65, 139.49, 123.87, 115.51, 72.05, 70.81, 70.68, 70.20, 70.08, 59.09, 18.90, 18.71, 12.15, 11.80. MALDI-TOF calculated for [C29H58O4SSi2+Na$^+$] 581.349, found 581.225.

(5-bromo-4-(2-(2-(2-methoxyethoxy)ethoxy)ethoxy)thiophen-2-yl)triisopropylsilane (7)

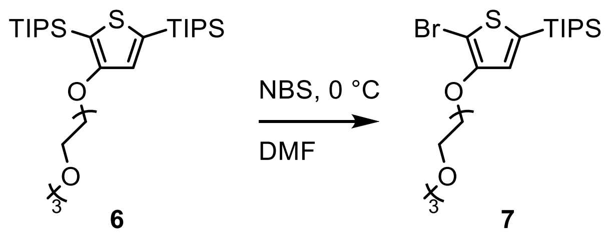

Compound **6** (2.12 g, 3.79 mmol) was dissolved in anhydrous DMF (11 ml) and reaction mixture was cooled down to 0 °C and protected against light. NBS (0.71 g, 3.98 mmol) dissolved in anhydrous DMF (11 ml) was added dropwise and reaction mixture was allowed to stir over night. Then, it was poured into water (40 ml) and extracted with EtAc (3x40 ml). Combined organic extracts were washed with water (3x40 ml), brine (2x40 ml), dried with $MgSO_4$ and evaporated. Silica column chromatography using hexane and EtAc (6/1) as an eluent provided title compound as a light orange oil (1.65 g, 3.43 mmol, 90 %). ^{1}H NMR (400 MHz, $CDCl_3$) δ 6.85 (s, 1H), 4.24 – 4.18 (m, 2H), 3.86 – 3.79 (m, 2H), 3.77 – 3.72 (m, 2H), 3.70 – 3.63 (m, 4H), 3.55 (dd, J = 5.8, 3.6 Hz, 2H), 3.40 – 3.35 (m, 3H), 1.33 – 1.23 (m, 3H), 1.08 (d, J = 7.3 Hz, 18H). ^{13}C NMR (101 MHz, $CDCl_3$) δ 155.32, 133.47, 125.19, 96.73, 71.79, 71.46, 70.80, 70.50, 70.38, 69.72, 58.80, 18.32, 11.34. HRMS Calculated for [C20H37BrO4SSi+H$^+$] 481.1455, found 481.1455.

triisopropyl(4-(2-(2-(2-methoxyethoxy)ethoxy)ethoxy)thiophen-2-yl)silane (8)

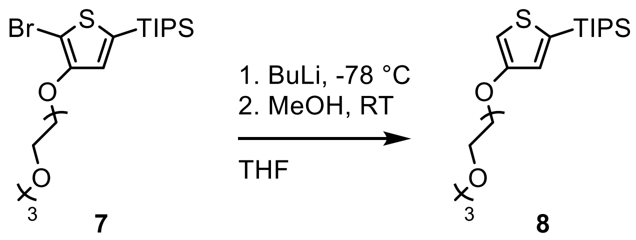

Compound **7** (1.62 g, 3.36 mmol) was dissolved in anhydrous THF (16 ml) in nitrogen atmosphere and the reaction mixture was cooled down to -78 °C. Butyllithium (1.41 ml, 3.53 mmol, 2.5M solution in hexanes) was added dropwise and reaction mixture was stirred at -78 °C one hour. Then methanol (1 ml) was added, and reaction mixture was allowed to warm up over one hour. Then it was poured into water (20 ml) and extracted with EtAc (3x20 ml). Combined organic extracts were washed with water (3x20 ml), brine (2x20 ml), dried with $MgSO_4$ and evaporated. Silica column chromatography using hexane and EtAc (4/1) as an eluent provided title compound as light-yellow oil (1.10 g, 2.73 mmol, 81 %). ^{1}H NMR (400 MHz, $CDCl_3$) δ 6.90 (d, J = 1.2 Hz, 1H), 6.50 (d, J = 1.1 Hz, 1H), 4.12 – 4.06 (m, 2H), 3.85 – 3.79 (m, 2H), 3.70 (dd, J = 5.7, 3.3 Hz, 2H), 3.67 – 3.60 (m, 4H), 3.52 (dd, J = 5.8, 3.6 Hz, 2H), 3.34 (s, 3H), 1.33 – 1.21 (m, 3H), 1.07 (d, J = 7.4 Hz, 18H). ^{13}C NMR (101 MHz, $CDCl_3$) δ 158.76, 133.60, 127.51, 102.81, 71.96, 70.80, 70.67, 70.58, 69.81, 69.66, 59.00, 18.55, 11.62. HRMS Calculated for [C20H386O4SSi+H$^+$] 403.2338, found 403.2335.

((2,5-bis(2-(2-(2-methoxyethoxy)ethoxy)ethoxy)-1,4-phenylene)bis(3-(2-(2-(2-methoxyethoxy)-ethoxy)ethoxy)thiophene-5,2-diyl))bis(triisopropylsilane) (9)

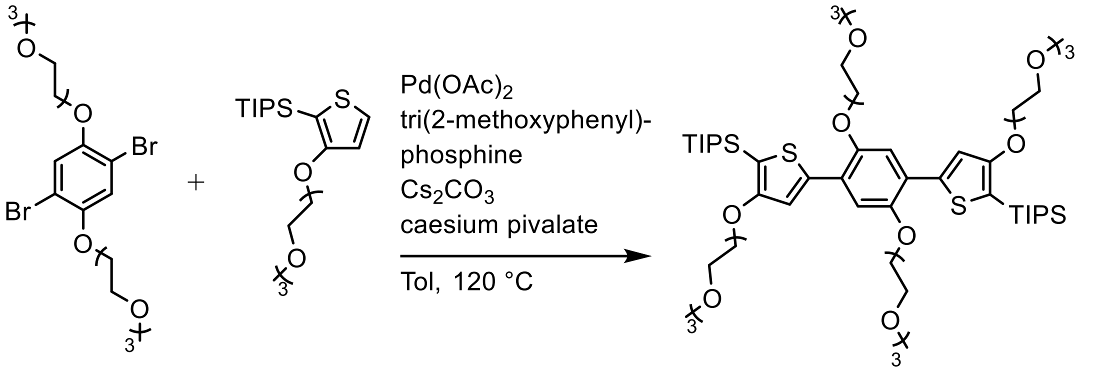

3 **5** **9**

Palladium acetate (11.2 mg, 49.7 µmol), tris(2-methoxyphenyl)phosphine (35.0 mg, 99.3 µmol), caesium carbonate (1.21 g, 3.73 mmol) and caesium pivalate (0.58 g, 2.48 mmol) were mixed together in sealed tube and the mixture was purged with nitrogen 15 min. Then, anhydrous toluene (10 ml) was added followed by compound **3** (0.63 g, 1.12 mmol) and compound **5** (1.00 g, 2.48 mmol). Reaction mixture was bubbled with nitrogen additional 30 min. Temperature was raised to 120 °C and reaction mixture was allowed to stir 80 hours. After cooling down reaction mixture was diluted with chloroform, filtered through celite pad, dried with anhydrous $MgSO_4$ and evaporated. Crude product was purified via silica chromatography using toluene/acetone (7/3) recovering title compound as brown oil (1.16 g, 0.96 mmol, 86 %). ^{1}H NMR (400 MHz, $CDCl_3$) δ 7.46 (s, 2H), 7.19 (s, 2H), 4.24 (t, *J* = 5.0 Hz, 4H), 4.16 (t, *J* = 5.2 Hz, 4H), 3.94 (t, *J* = 5.0 Hz, 4H), 3.79 (t, *J* = 5.1 Hz, 4H), 3.74 – 3.58 (m, 24H), 3.55 – 3.47 (m, 8H), 3.38 – 3.31 (m, 12H), 1.47 – 1.35 (m, 6H), 1.10 (d, *J* = 7.5 Hz, 36H). ^{13}C NMR (101 MHz, $CDCl_3$) δ 163.20, 149.45, 142.86, 123.26, 115.43, 112.69, 109.69, 71.99, 71.96, 70.87, 70.76, 70.74, 70.64, 70.61, 70.10, 69.93, 69.85, 68.98, 59.06, 59.03, 18.91, 12.15. HRMS Calculated for [C60H106O16S2Si2+H^+] 1203.6539, found 1203.6530.

5,5'-(2,5-bis(2-(2-(2-methoxyethoxy)ethoxy)ethoxy)-1,4-phenylene)bis(3-(2-(2-(2-methoxy-ethoxy)ethoxy)ethoxy)thiophene) (11)

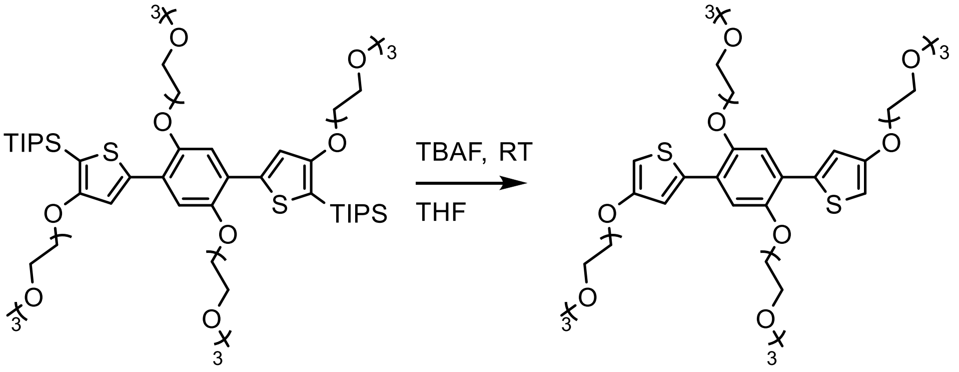

9 **11**

Compound **9** (1.09 g, 0.91 mmol) was dissolved in anhydrous THF (23 ml) in nitrogen atmosphere. Tetrabutylammonium fluoride (2.26 ml, 2.26 mmol, 1M solution in tetrahydrofuran) was added dropwise at RT and reaction mixture was allowed to stir at RT 2 hours. NH_4Cl (1 spatula) was then added, and reaction mixture was filtered and evaporated. Silica column chromatography using

toluene/acetone (1/1) as eluent provided title compound as brown oil (0.72 g, 0.80 mmol, 89 %). ^{1}H NMR (400 MHz, $CDCl_3$) δ 7.20 (d, *J* = 1.7 Hz, 2H), 7.14 (s, 2H), 6.22 (d, *J* = 1.7 Hz, 2H), 4.18 – 4.15 (m, 4H), 4.10 – 4.06 (m, 4H), 3.89 – 3.85 (m, 4H), 3.82 – 3.77 (m, 4H), 3.71 – 3.67 (m, 8H), 3.65 – 3.58 (m, 16H), 3.51 – 3.45 (m, 8H), 3.32 (s, 6H), 3.30 (s, 6H). ^{13}C NMR (101 MHz, $CDCl_3$) δ 156.81, 149.44, 137.42, 123.11, 117.79, 112.81, 98.22, 71.82, 70.75, 70.68, 70.58, 70.55, 70.45, 70.45, 69.65, 69.60, 69.22, 68.98, 58.90, 58.88. HRMS Calculated for [C60H106O16S2Si2+H^{+}] 891.3871, found 891.3870.

((2,5-bis(2-(2-(2-methoxyethoxy)ethoxy)ethoxy)-1,4-phenylene)bis(4-(2-(2-(2-methoxyethoxy)-ethoxy)ethoxy)thiophene-5,2-diyl))bis(triisopropylsilane) (10)

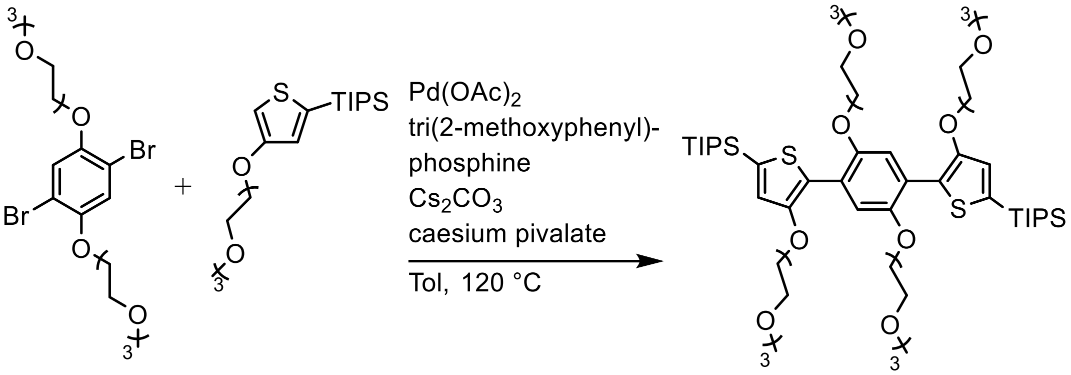

3 8 10

Palladium acetate (30.1 mg, 134 µmol), tris(2-methoxyphenyl)phosphine (94.5 mg, 268 µmol), caesium carbonate (3.28 g, 10.1 mmol) and caesium pivalate (1.57 g, 6.71 mmol) were mixed together in sealed tube and the mixture was purged with nitrogen 15 min. Then, anhydrous toluene (27 ml) was added followed by compound **8** (2.70 g, 6.71 mmol) and compound **3** (1.69 g, 3.02 mmol). Reaction mixture was bubbled with nitrogen additional 30 min. Temperature was raised to 120 °C and reaction mixture was allowed to stir 80 hours. After cooling down reaction mixture was diluted with chloroform, filtered through celite pad, dried with anhydrous $MgSO_4$ and evaporated. Crude product was purified via silica chromatography using toluene/acetone (7/3) recovering title compound* as brown oil (2.65 g, 2.20 mmol, 73 %).

**Compound 10 could not be purified fully by column chromatography (several attempts were made with different conditions). Therefore, the crude product was used in the next step to prepare Compound 12 after which purification was successful.*

2,2'-(2,5-bis(2-(2-(2-methoxyethoxy)ethoxy)ethoxy)-1,4-phenylene)bis(3-(2-(2-(2-methoxyethoxy)-ethoxy)ethoxy)thiophene) (12)

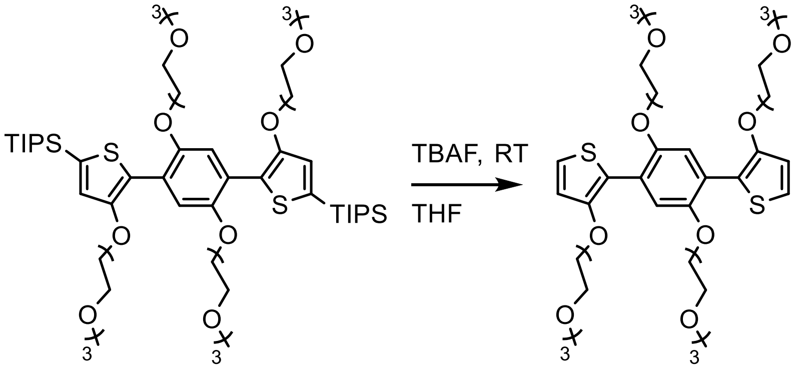

Compound **10** (2.56 g, 2.13 mmol) was dissolved in anhydrous THF (53 ml) in nitrogen atmosphere. Tetrabutylammonium fluoride (5.32 ml, 5.32 mmol, 1M solution in tetrahydrofuran) was added dropwise at RT and reaction mixture was allowed to stir at RT 2 hours. NH_4Cl (1 spatula) was then added, and reaction mixture was filtered and evaporated. Silica column chromatography using toluene/acetone (1/1) as eluent provided title compound as yellow oil (1.35 g, 1.52 mmol, 71 %). ^{1}H NMR (400 MHz, $CDCl_3$) δ 7.76 (s, 2H), 7.19 (d, *J* = 5.6 Hz, 2H), 6.89 (d, *J* = 5.6 Hz, 2H), 4.22 – 4.17 (m, 8H), 3.93 – 3.88 (m, 4H), 3.82 – 3.78 (m, 4H), 3.74 – 3.70 (m, 4H), 3.68 – 3.58 (m, 20H), 3.54 – 3.48 (m, 8H), 3.36 (s, 6H), 3.35 (s, 6H). ^{13}C NMR (101 MHz, $CDCl_3$) δ 153.92, 148.73, 123.75, 121.41, 117.06, 116.64, 114.32, 72.05, 72.00, 70.97, 70.89, 70.80, 70.79, 70.74, 70.64, 70.10, 69.86, 69.07, 59.12, 59.09. HRMS Calculated for [C42H66O16S2+H$^+$] 891.3870, found 891.3861.

(4,4''-bis(2-(2-(2-methoxyethoxy)ethoxy)ethoxy)-[2,2':5',2''-terthiophene]-5,5''-diyl)bis-(triisopropylsilane) (13)

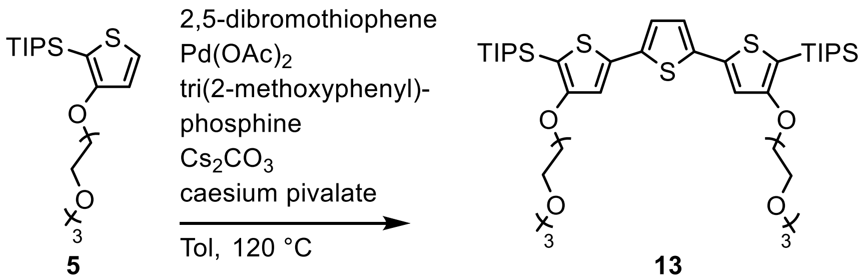

Palladium acetate (33.5 mg, 149 µmol), tris(2-methoxyphenyl)phosphine (105 mg, 298 µmol), caesium carbonate (3.64 g, 11.2 mmol) and caesium pivalate (1.74 g, 7.45 mmol) were mixed together in sealed tube and the mixture was purged with nitrogen 15 min. Then, anhydrous toluene (30 ml) was added followed by compound **5** (3.00 g, 7.45 mmol) and 2,5-dibromothiophene (0.81 g, 3.35 mmol). Reaction mixture was bubbled with nitrogen additional 30 min. Temperature was raised to 120 °C and reaction mixture was allowed to stir 80 hours. After cooling down reaction mixture was diluted with chloroform, filtered through celite pad, dried with anhydrous $MgSO_4$ and evaporated. Crude product was purified via silica chromatography using hexane/EtAc (1/1) recovering title compound as brown oil (0.48 g, 0.54 mmol, 16 %). ^{1}H NMR (400 MHz, $CDCl_3$) δ 7.05 (s, 2H), 7.01 (s, 2H), 4.16 – 4.11 (m, 4H), 3.80 – 3.76 (m, 4H), 3.71 – 3.62 (m, 12H), 3.56 – 3.51 (m, 4H), 3.36 (s, 6H), 1.45 – 1.33 (m, 6H), 1.10 (d, *J* = 7.4 Hz, 36H). ^{13}C NMR (101 MHz, $CDCl_3$) δ 163.22, 140.57, 136.44, 123.75, 113.06, 109.17, 71.96, 70.76, 70.73, 70.62,

70.12, 70.02, 59.02, 18.80, 12.08. MALDI-TOF calculated for [C44H76O8S3Si2+H$^+$] 885.432, found 885.296.

4,4''-bis(2-(2-(2-methoxyethoxy)ethoxy)ethoxy)-2,2':5',2''-terthiophene (15)

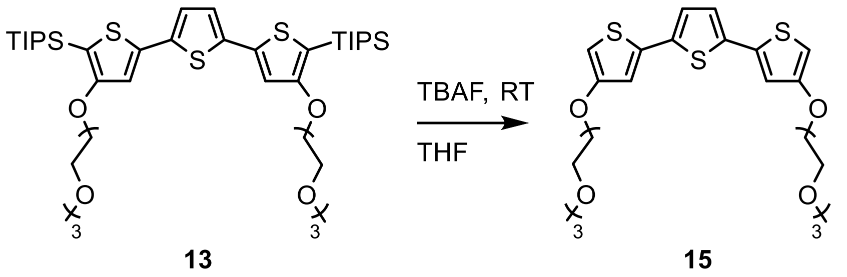

Compound **13** (0.51 g, 0.58 mmol) was dissolved in anhydrous THF (15 ml) in nitrogen atmosphere. Tetrabutylammonium fluoride (1.45 ml, 1.45 mmol, 1M solution in tetrahydrofuran) was added dropwise at RT and reaction mixture was allowed to stir at RT 2 hours. NH_4Cl (1 spatula) was then added, and reaction mixture was filtered and evaporated. Silica column chromatography using EtAc as eluent provided title compound as brown oil (0.29 g, 0.50 mmol, 86 %). ^{1}H NMR (400 MHz, $CDCl_3$) δ 7.03 (s, 2H), 6.86 (d, *J* = 1.6 Hz, 2H), 6.17 (d, *J* = 1.7 Hz, 2H), 4.15 – 4.11 (m, 4H), 3.87 – 3.82 (m, 4H), 3.76 – 3.71 (m, 4H), 3.71 – 3.64 (m, 8H), 3.58 – 3.53 (m, 4H), 3.38 (s, 6H). ^{13}C NMR (101 MHz, $CDCl_3$) δ 157.19, 136.50, 135.45, 124.06, 116.07, 96.95, 71.93, 70.81, 70.65, 70.57, 69.63, 69.51, 59.02. HRMS Calculated for [C26H366O8S3+H$^+$] 573.1650, found 573.1663.

(3,3''-bis(2-(2-(2-methoxyethoxy)ethoxy)ethoxy)-[2,2':5',2''-terthiophene]-5,5''-diyl)bis-(triisopropylsilane) (14)

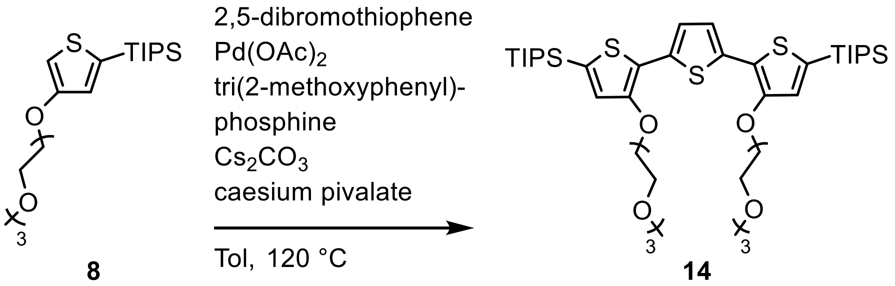

Palladium acetate (22.3 mg, 99.3 µmol), tris(2-methoxyphenyl)phosphine (70.0 mg, 199 µmol), caesium carbonate (2.43 g, 7.45 mmol) and caesium pivalate (1.16 g, 4.97 mmol) were mixed together in sealed tube and the mixture was purged with nitrogen 15 min. Then, anhydrous toluene (20 ml) was added followed by compound **8** (2.00 g, 4.97 mmol) and 2,5-dibromothiophene (0.54 g, 2.24 mmol). Reaction mixture was bubbled with nitrogen additional 30 min. Temperature was raised to 120 °C and reaction mixture was allowed to stir 80 hours. After cooling down reaction mixture was diluted with chloroform, filtered through celite pad, dried with anhydrous $MgSO_4$ and evaporated. Crude product was purified via silica chromatography using hexane/EtAc (1/1) recovering title compound as brown oil (0.74 g, 0.83 mmol, 37 %). ^{1}H NMR (400 MHz, $CDCl_3$) δ 7.21 (s, 2H), 6.95 (s, 2H), 4.30 – 4.24 (m, 4H), 3.92 – 3.88 (m, 4H), 3.79 – 3.73 (m, 4H), 3.69 – 3.59 (m, 8H), 3.53 – 3.48 (m, 4H), 3.34 (s, 6H), 1.38 – 1.25 (m, 6H), 1.11 (d, *J* = 7.4 Hz, 36H). ^{13}C NMR (101 MHz, $CDCl_3$) δ 153.24, 133.37, 130.01, 125.63, 123.21, 121.93, 71.92, 71.25, 70.98, 70.71, 70.55, 70.07, 58.98, 18.58, 11.63. MALDI-TOF calculated for [C44H76O8S3Si2+H$^+$] 885.432, found 885.142.

3,3''-bis(2-(2-(2-methoxyethoxy)ethoxy)ethoxy)-2,2':5',2''-terthiophene (16)

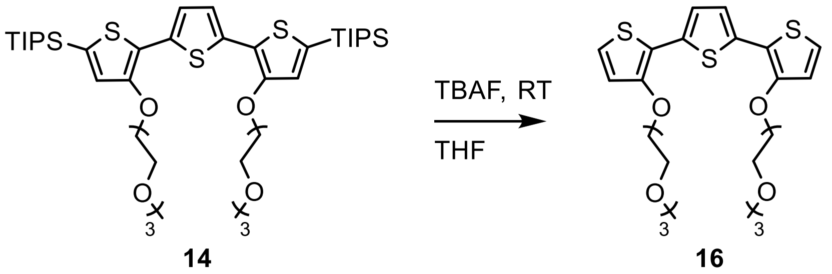

Compound **14** (0.64 g, 0.72 mmol) was dissolved in anhydrous THF (18 ml) in nitrogen atmosphere. Tetrabutylammonium fluoride (1.80 ml, 1.80 mmol, 1M solution in tetrahydrofuran) was added dropwise at RT and reaction mixture was allowed to stir at RT 2 hours. NH_4Cl (1 spatula) was then added, and reaction mixture was filtered and evaporated. Silica column chromatography using EtAc as eluent provided title compound as brown oil (0.31 g, 0.55 mmol, 77 %). ^{1}H NMR (400 MHz, $CDCl_3$) δ 7.16 (s, 2H), 7.00 (d, *J* = 5.5 Hz, 2H), 6.84 (d, *J* = 5.6 Hz, 2H), 4.25 – 4.21 (m, 4H), 3.89 – 3.83 (m, 4H), 3.75 – 3.70 (m, 4H), 3.67 – 3.58 (m, 8H), 3.52 – 3.47 (m, 4H), 3.33 (s, 6H). ^{13}C NMR (101 MHz, $CDCl_3$) δ 152.13, 133.20, 123.19, 121.25, 118.16, 116.59, 71.91, 71.31, 70.94, 70.67, 70.54, 70.01, 58.99. HRMS Calculated for [C26H366O8S3+H$^+$] 573.1650, found 573.1663.

poly(2-(2,5-bis(2-(2-(2-methoxyethoxy)ethoxy)ethoxy)-4-(3-(2-(2-(2-methoxyethoxy)ethoxy)-ethoxy)thiophen-2-yl)phenyl)-3-(2-(2-(2-methoxyethoxy)ethoxy)ethoxy)-5-phenylthiophene) (inDTP-P)

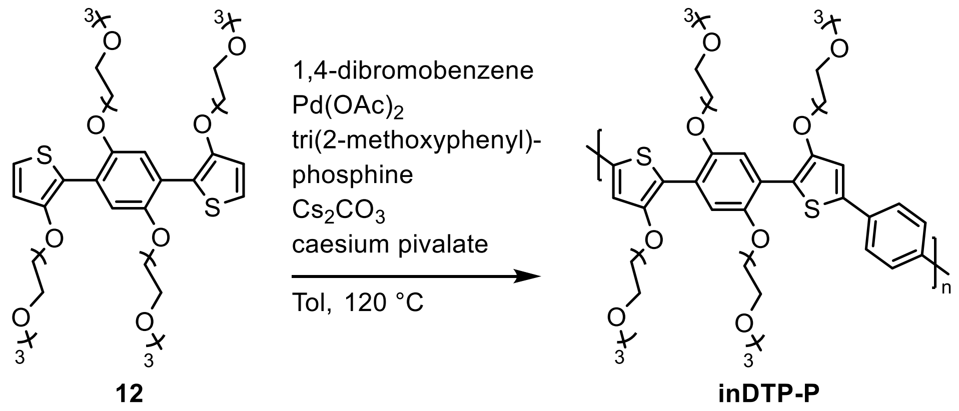

Palladium acetate (2.00 mg, 8.98 μmol), tris(2-methoxyphenyl)phosphine (6.30 mg, 18.0 μmol), caesium carbonate (0.22 g, 0.67 mmol), caesium pivalate (52.5 mg, 0.22 mmol) and 1,4-dibromobenzene (52.9 mg, 0.22 mmol) were mixed together in sealed tube and the mixture was purged with nitrogen 15 min. Then, anhydrous toluene (1.5 ml) was added followed by compound **12** (0.20 g, 0.22 mmol. Reaction mixture was bubbled with nitrogen additional 30 min. Temperature was raised to 120 °C and reaction mixture was allowed to stir 80 hours. After cooling down, reaction mixture was diluted with chloroform (5 ml) and the solution was added dropwise into well-stirred methanol (50 ml). Precipitate was filtered into Soxhlet thimble and extracted with hexane (16 h), methanol (6 h) and acetone (16 h). Purified product was recovered via chloroform extraction and evaporation as a dark red solid (183 mg, 84 %). ^{1}H NMR (400 MHz, TCE-d2) δ 7.95 (s, 2H), 7.73 (s, 4H), 7.29 (s, 2H), 4.45 – 4.33 (m, 8H), 4.12 – 4.02 (m, 4H), 4.01 – 3.92 (m, 4H), 3.86 – 3.65 (m, 24H), 3.61 – 3.54 (m, 8H), 3.41 (d, *J* = 8.2 Hz, 12H).

poly(5-(2,5-bis(2-(2-(2-methoxyethoxy)ethoxy)ethoxy)-4-(4-(2-(2-(2-methoxyethoxy)ethoxy)-ethoxy)thiophen-2-yl)phenyl)-3-(2-(2-(2-methoxyethoxy)ethoxy)ethoxy)-2-phenylthiophene) (outDTP-P)

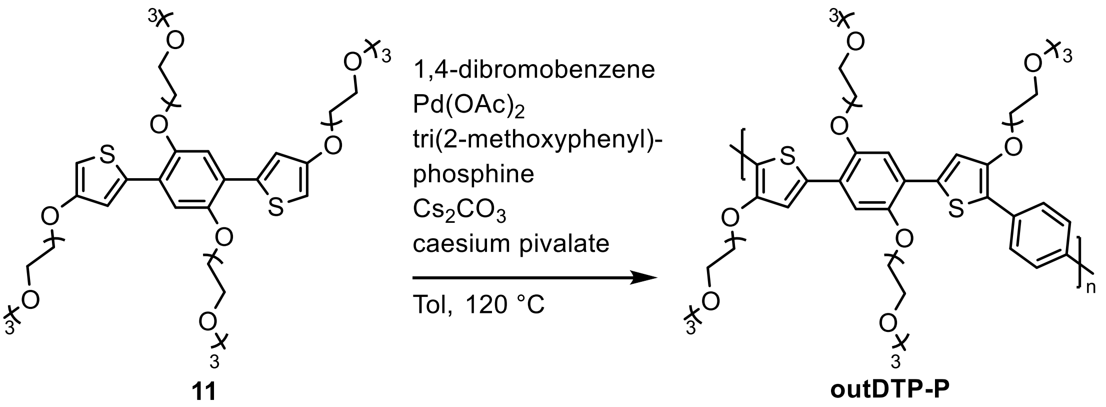

Palladium acetate (2.00 mg, 8.98 µmol), tris(2-methoxyphenyl)phosphine (6.30 mg, 18.0 µmol), caesium carbonate (0.22 g, 0.67 mmol), caesium pivalate (52.5 mg, 0.22 mmol) and 1,4-dibromobenzene (52.9 mg, 0.22 mmol) were mixed together in sealed tube and the mixture was purged with nitrogen 15 min. Then, anhydrous toluene (1.5 ml) was added followed by compound **11** (0.20 g, 0.22 mmol. Reaction mixture was bubbled with nitrogen additional 30 min. Temperature was raised to 120 °C and reaction mixture was allowed to stir 80 hours. After cooling down, reaction mixture was diluted with chloroform (5 ml) and the solution was added dropwise into well-stirred methanol (50 ml). Precipitate was filtered into Soxhlet thimble and extracted with hexane (16 h), methanol (6 h) and acetone (16 h). Purified product was recovered via chloroform extraction and evaporation as a dark red solid (173 mg, 80 %). ^{1}H NMR (400 MHz, TCE-d2) δ 7.88 (s, 4H), 7.55 (s, 2H), 7.33 (s, 2H), 4.37 (t, *J* = 4.9 Hz, 8H), 4.03 (t, *J* = 4.9 Hz, 4H), 3.97 – 3.92 (m, 4H), 3.83 – 3.62 (m, 24H), 3.59 – 3.51 (m, 8H), 3.37 (t, *J* = 3.9 Hz, 12H).

poly(5-(2,5-bis(2-(2-(2-methoxyethoxy)ethoxy)ethoxy)-4-(3-(2-(2-(2-methoxyethoxy)ethoxy)-ethoxy)thiophen-2-yl)phenyl)-4-(2-(2-(2-methoxyethoxy)ethoxy)ethoxy)-2,2'-bithiophene) (inDTP-T)

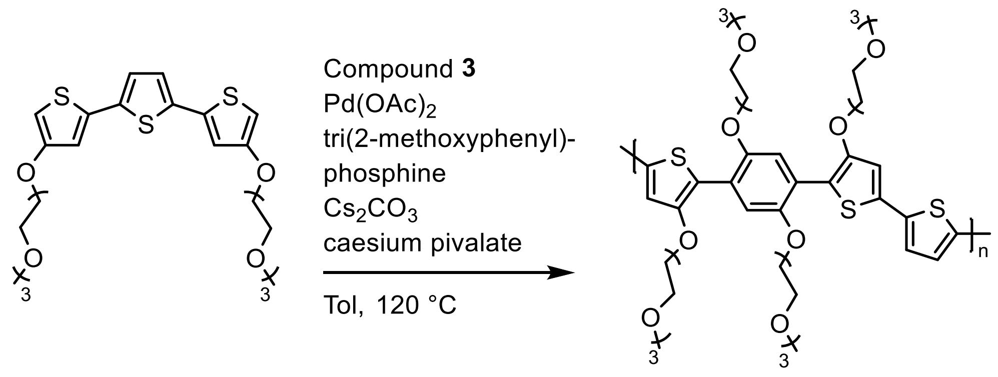

Palladium acetate (3.40 mg, 14.9 µmol), tris(2-methoxyphenyl)phosphine (10.5 mg, 29.9 µmol), caesium carbonate (0.37 g, 1.12 mmol) and caesium pivalate (87.4 mg, 0.37 mmol) were mixed together in sealed tube and the mixture was purged with nitrogen 15 min. Then, anhydrous toluene (2.5 ml) was added followed by compound **15** (0.21 g, 0.37 mmol) and compound **3** (0.21 g, 0.37 mmol). Reaction mixture was bubbled with nitrogen additional 30 min. Temperature was raised

to 120 °C and reaction mixture was allowed to stir 80 hours. After cooling down, reaction mixture was diluted with chloroform (5 ml) and the solution was added dropwise into well-stirred methanol (50 ml). Precipitate was filtered into Soxhlet thimble and extracted with hexane (16 h), methanol (6 h) and acetone (16 h). Purified product was recovered via chloroform extraction and evaporation as a dark blue solid (261 mg, 72 %). ^{1}H NMR (400 MHz, TCE-d2) δ 7.97 (s, 2H), 7.22 (s, 2H), 7.12 (s, 2H), 4.43 – 4.30 (m, 8H), 4.05 (t, *J* = 4.8 Hz, 4H), 3.98 – 3.92 (m, 4H), 3.87 – 3.81 (m, 4H), 3.79 – 3.66 (m, 20H), 3.61 – 3.54 (m, 8H), 3.41 (d, *J* = 6.3 Hz, 12H).

poly(5-(2,5-bis(2-(2-(2-methoxyethoxy)ethoxy)ethoxy)-4-(4-(2-(2-(2-methoxyethoxy)ethoxy)-ethoxy)thiophen-2-yl)phenyl)-3-(2-(2-(2-methoxyethoxy)ethoxy)ethoxy)-2,2'-bithiophene) (outDTP-T)

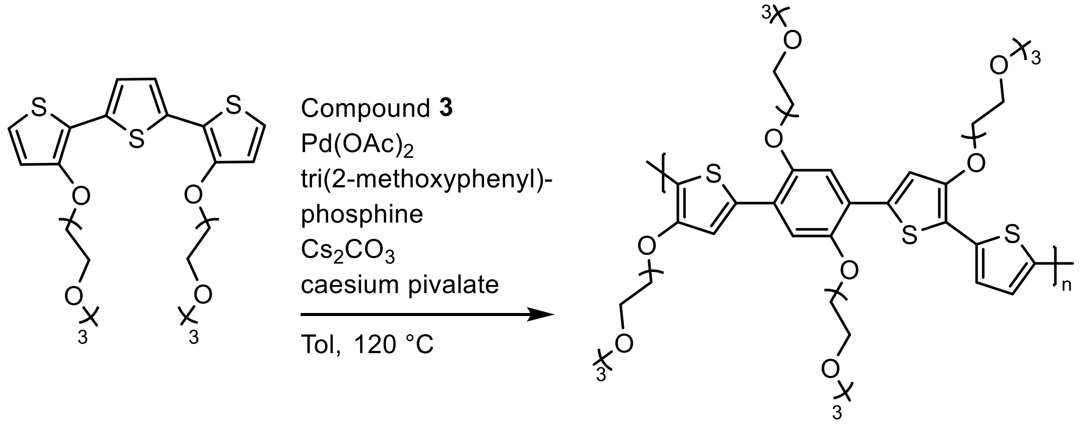

16 **outDTP-T**

Palladium acetate (3.90 mg, 17.5 µmol), tris(2-methoxyphenyl)phosphine (12.4 mg, 35.0 µmol), caesium carbonate (0.42 g, 1.31 mmol) and caesium pivalate (0.10 g, 0.44 mmol) were mixed together in sealed tube and the mixture was purged with nitrogen 15 min. Then, anhydrous toluene (2.9 ml) was added followed by compound **16** (0.25 g, 0.44 mmol) and compound **3** (0.25 g, 0.44 mmol). Reaction mixture was bubbled with nitrogen additional 30 min. Temperature was raised to 120 °C and reaction mixture was allowed to stir 80 hours. After cooling down, reaction mixture was diluted with chloroform (20 ml), filtered through celite pad and evaporated. Crude product was dissolved in chloroform (5 ml) and the solution was added dropwise into well-stirred methanol (50 ml). Precipitate was filtered into Soxhlet thimble and extracted with hexane (16 h), methanol (6 h) and acetone (16 h). Purified product was recovered via chloroform extraction and evaporation as a dark blue solid (79 mg, 19 %). ^{1}H NMR (400 MHz, TCE-d2) δ 7.52 (s, 2H), 7.33 (s, 4H), 4.53 – 4.32 (m, 8H), 4.10 – 3.99 (m, 8H), 3.88 – 3.81 (m, 8H), 3.80 – 3.73 (m, 8H), 3.72 – 3.67 (m, 8H), 3.62 – 3.56 (m, 8H), 3.41 (s, 12H).

poly(5-(2,5-bis(2-(2-(2-methoxyethoxy)ethoxy)ethoxy)-4-(3-(2-(2-(2-methoxyethoxy)ethoxy)-ethoxy)thiophen-2-yl)phenyl)-4-(2-(2-(2-methoxyethoxy)ethoxy)ethoxy)-2,2':5',2''-terthiophene) (inDTP-2T)

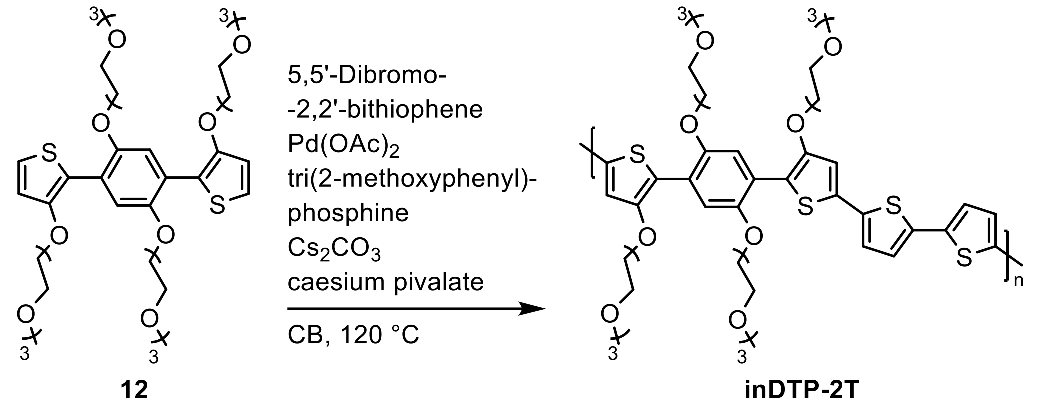

12 **inDTP-2T**

Palladium acetate (2.00 mg, 8.98 µmol), tris(2-methoxyphenyl)phosphine (6.30 mg, 18.0 µmol), caesium carbonate (0.22 g, 0.67 mmol), caesium pivalate (52.5 mg, 0.22 mmol) and 5,5′-dibromo-2,2′-bithiophene (72.7 mg, 0.22 mmol) were mixed together in sealed tube and the mixture was purged with nitrogen 15 min. Then, anhydrous chlorobenzene (1.5 ml) was added followed by compound **12** (0.20 g, 0.22 mmol. Reaction mixture was bubbled with nitrogen additional 30 min. Temperature was raised to 120 °C and reaction mixture was allowed to stir 80 hours. After cooling down, reaction mixture was diluted with chloroform (5 ml) and the solution was added dropwise into well-stirred methanol (50 ml). Precipitate was filtered into Soxhlet thimble and extracted with hexane (16 h), methanol (6 h) and acetone (16 h). Purified product was recovered via chloroform extraction and evaporation as a black solid (147 mg, 68 %). ^{1}H NMR (400 MHz, TCE-d2) δ 8.03 – 7.86 (m, 2H), 7.33 – 7.06 (m, 6H), 4.43 – 4.26 (m, 8H), 4.12 – 3.80 (m, 12H), 3.78 – 3.62 (m, 20H), 3.60 – 3.53 (m, 8H), 3.44 – 3.30 (m, 12H).

poly(5-(2,5-bis(2-(2-(2-methoxyethoxy)ethoxy)ethoxy)-4-(4-(2-(2-(2-methoxyethoxy)ethoxy)-ethoxy)thiophen-2-yl)phenyl)-3-(2-(2-(2-methoxyethoxy)ethoxy)ethoxy)-2,2':5',2''-terthiophene) (outDTP-2T)

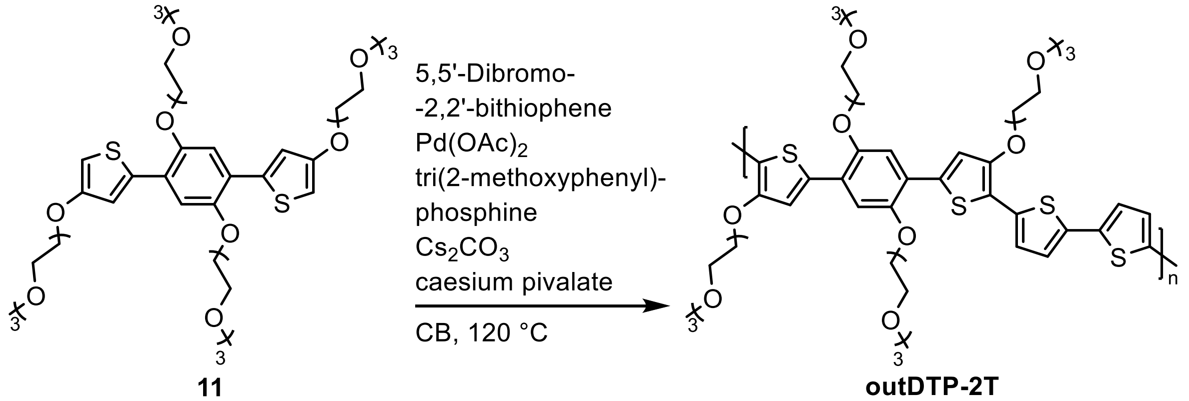

11 **outDTP-2T**

Palladium acetate (2.00 mg, 8.98 µmol), tris(2-methoxyphenyl)phosphine (6.30 mg, 18.0 µmol), caesium carbonate (0.22 g, 0.67 mmol), caesium pivalate (52.5 mg, 0.22 mmol) and 5,5′-dibromo-2,2′-bithiophene (72.7 mg, 0.22 mmol) were mixed together in sealed tube and the mixture was purged with nitrogen 15 min. Then, anhydrous chlorobenzene (1.5 ml) was added followed by compound **11** (0.20 g, 0.22 mmol. Reaction mixture was bubbled with nitrogen additional 30 min. Temperature was raised to 120 °C and reaction mixture was allowed to stir 80 hours. After cooling down, reaction mixture was diluted with chloroform (5 ml) and the solution was added dropwise into well-stirred methanol (50 ml). Precipitate was filtered into Soxhlet thimble and extracted with hexane (16 h),

methanol (6 h) and acetone (16 h). Purified product was recovered via chloroform extraction and evaporation as a black solid (171 mg, 79 %). ^{1}H NMR (400 MHz, TCE-d2) δ 7.48 (s, 2H), 7.29 (s, 4H), 7.16 (s, 2H), 4.47 – 4.29 (m, 8H), 4.01 (dt, J = 15.6, 4.7 Hz, 8H), 3.85 – 3.61 (m, 26H), 3.56 (d, J = 3.4 Hz, 8H), 3.41 – 3.31 (m, 12H).

3 NMR spectra

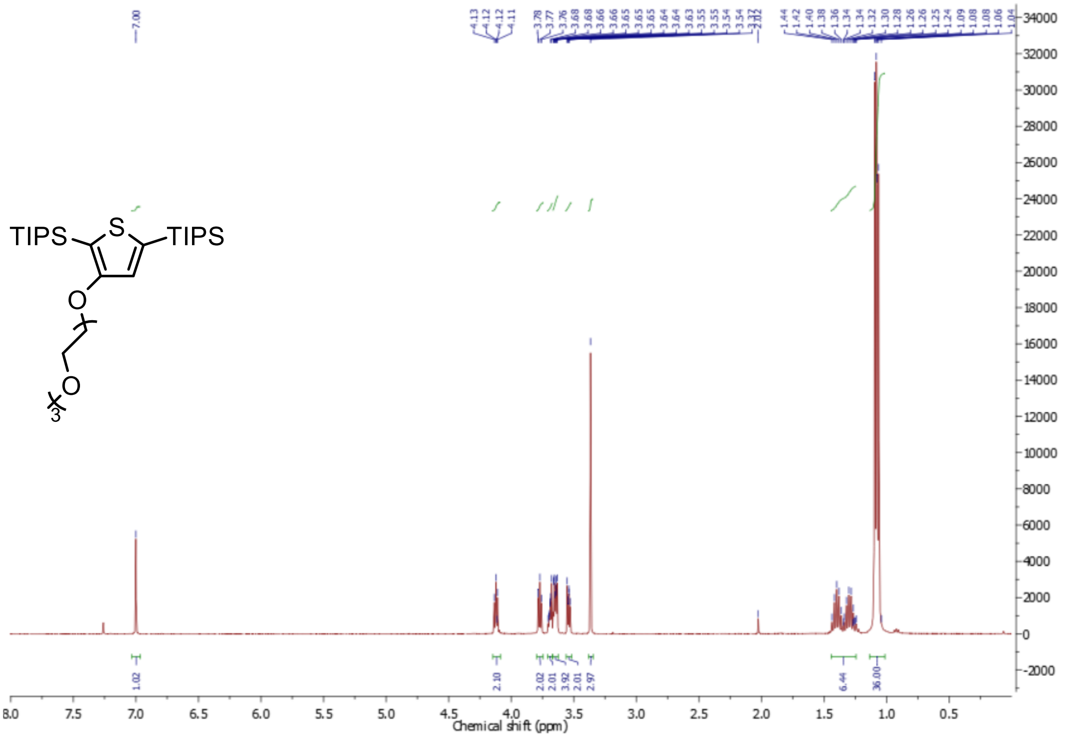

Figure S1: ^{1}H NMR spectrum of compound **6**

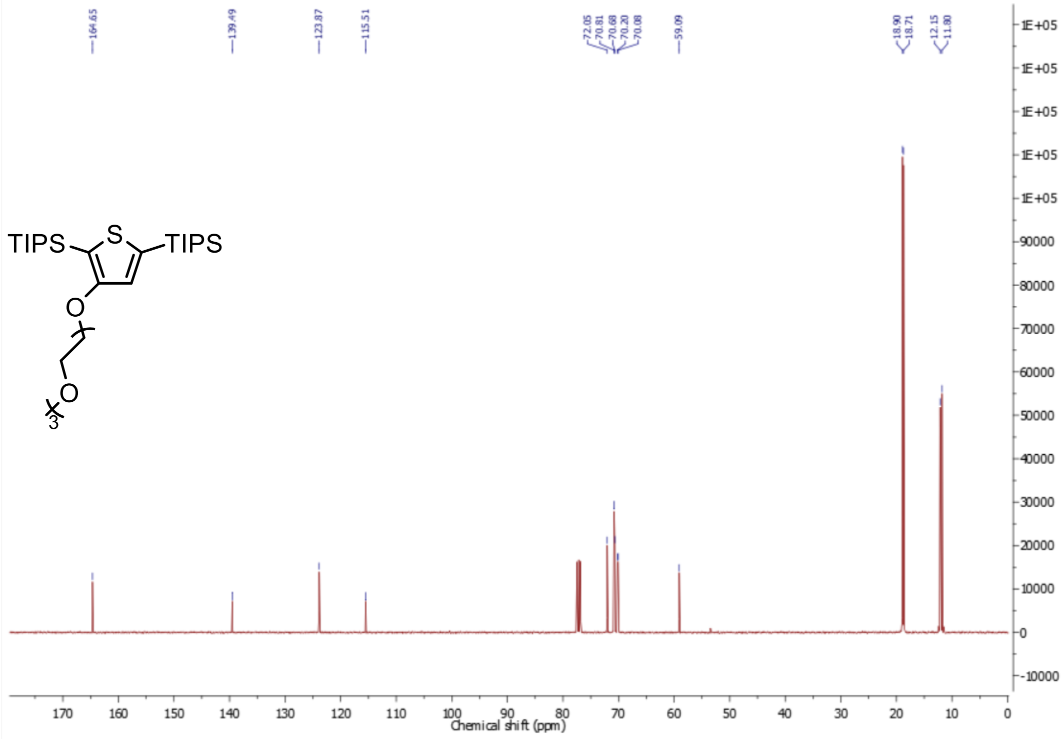

Figure S2: ^{13}C NMR spectrum of compound **6**

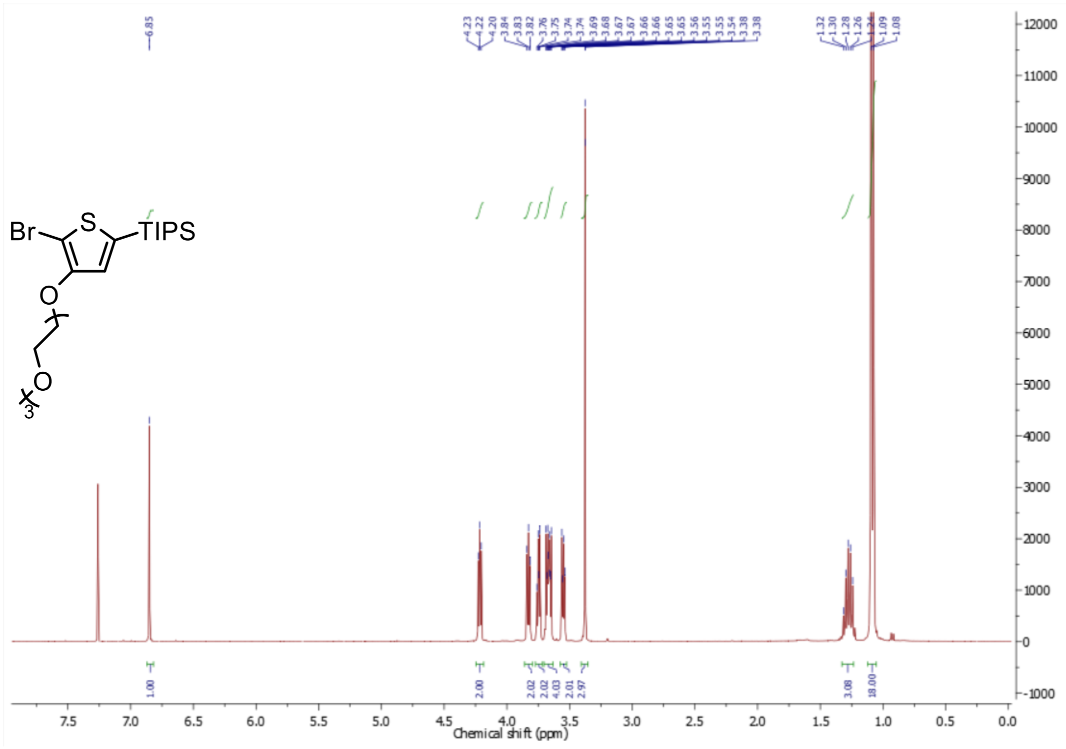

Figure S3: ^{1}H NMR spectrum of compound **7**

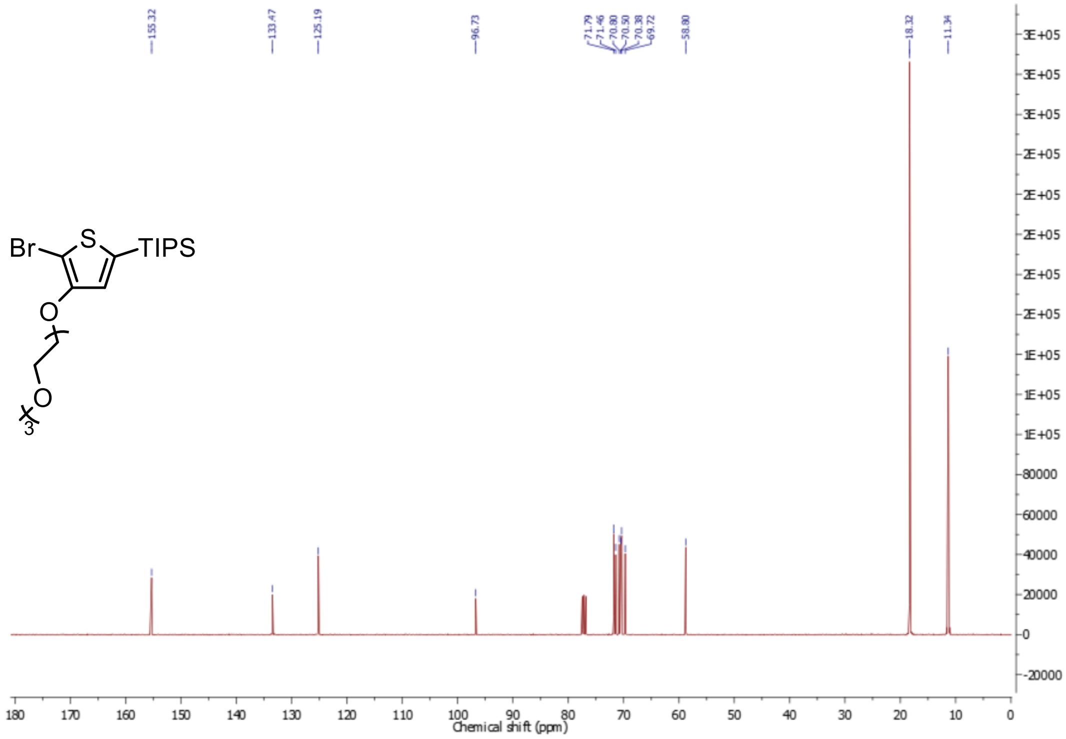

Figure S4: ^{13}C NMR spectrum of compound **7**

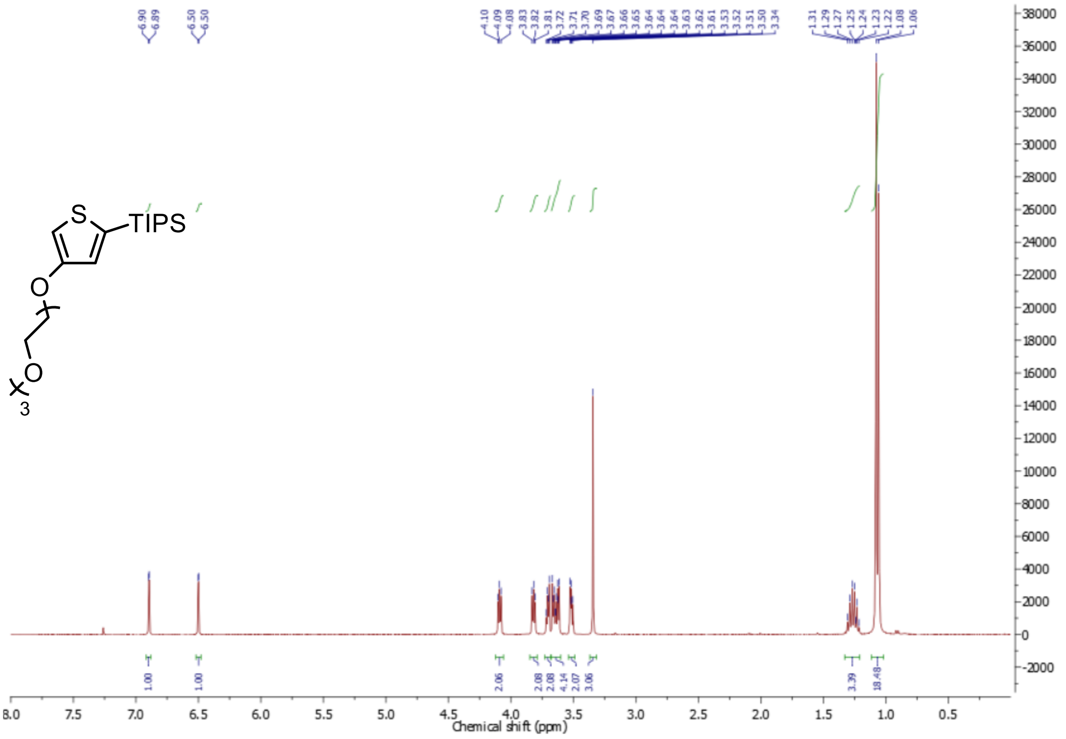

Figure S5: ^{1}H NMR spectrum of compound **8**

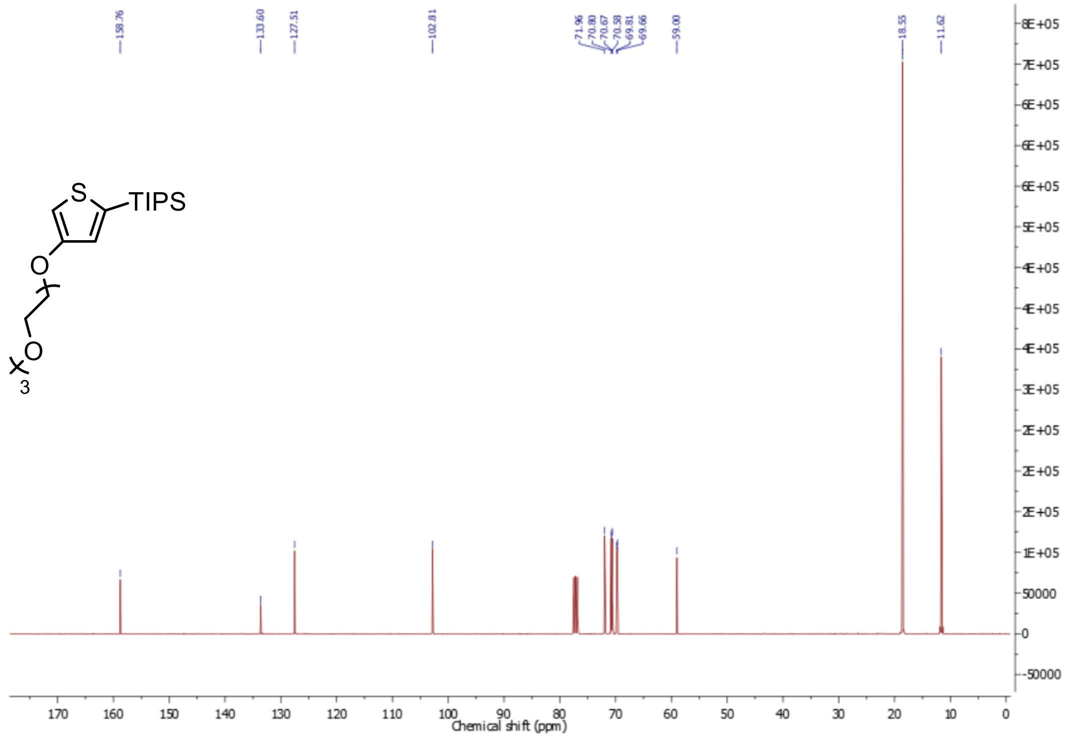

Figure S6: ^{13}C NMR spectrum of compound **8**

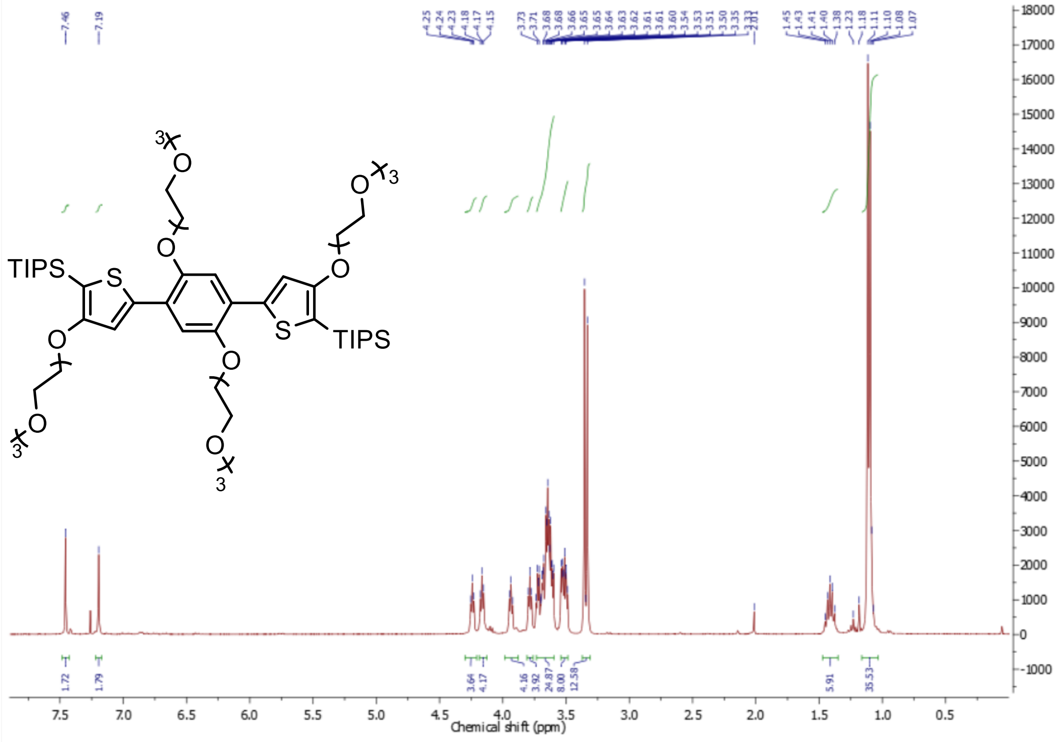

Figure S7: ^{1}H NMR spectrum of compound **9**

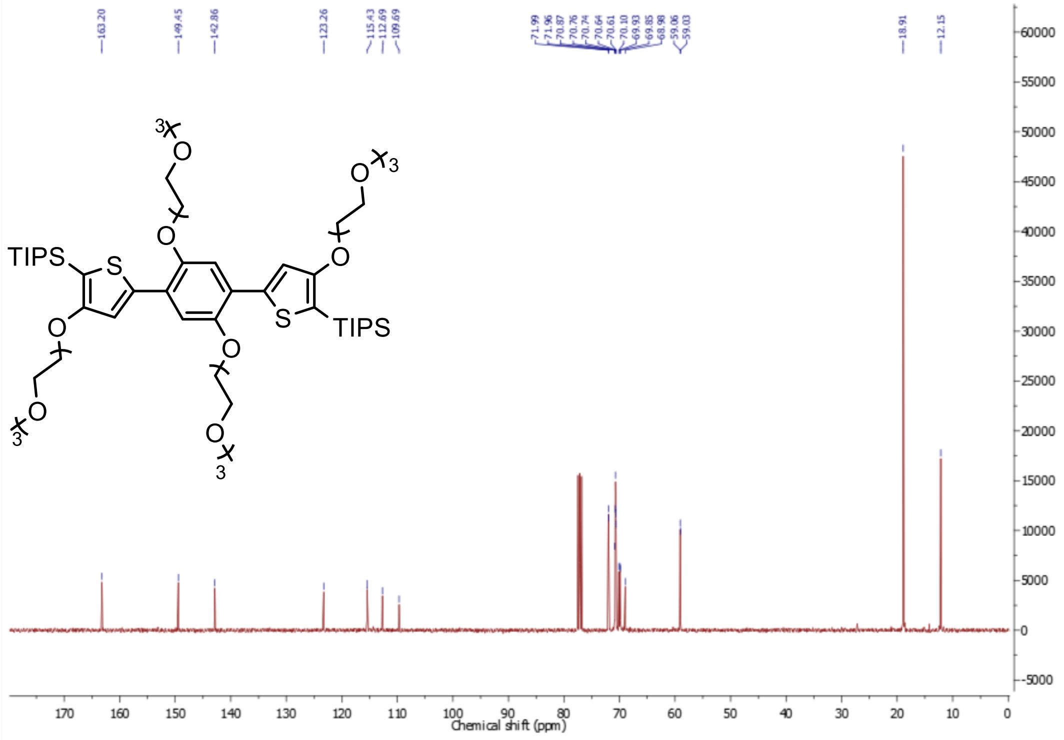

Figure S8: ^{13}C NMR spectrum of compound **9**

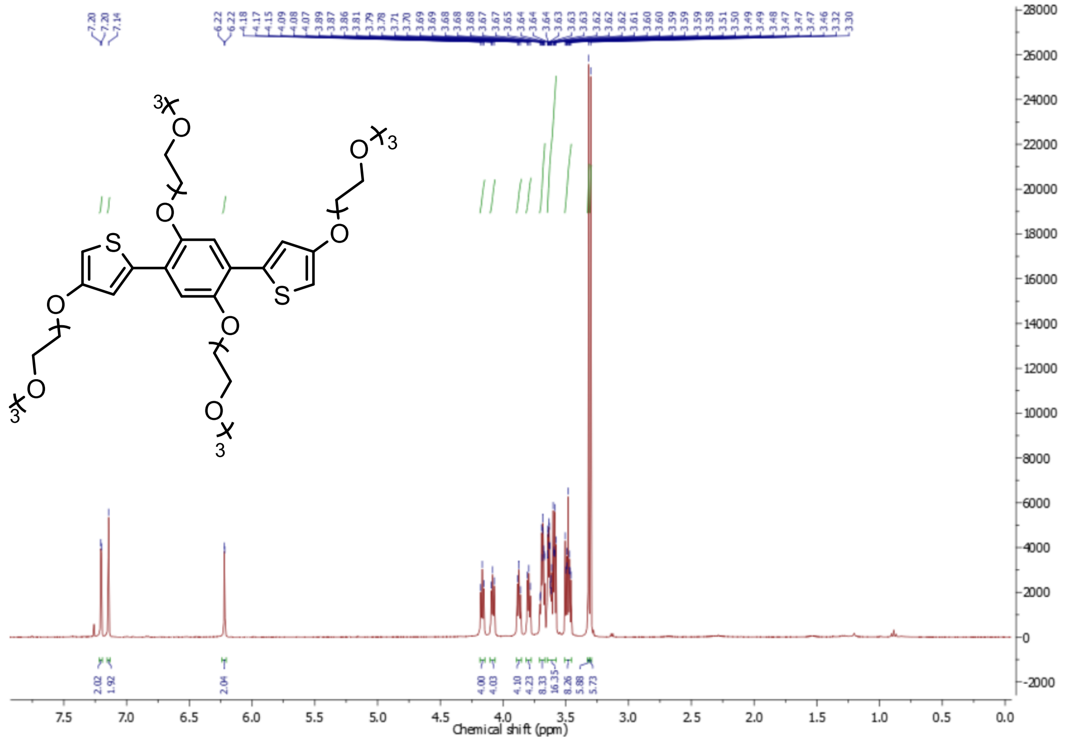

Figure S9: ^{1}H NMR spectrum of compound **11**

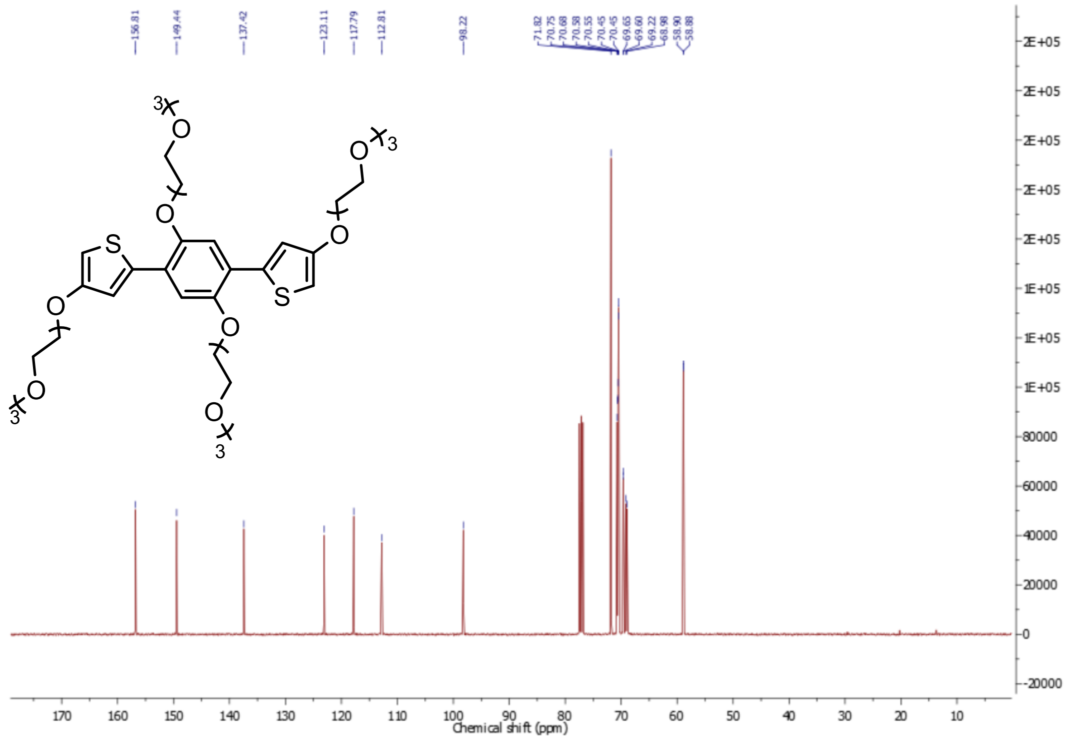

Figure S10: ^{13}C NMR spectrum of compound **11**

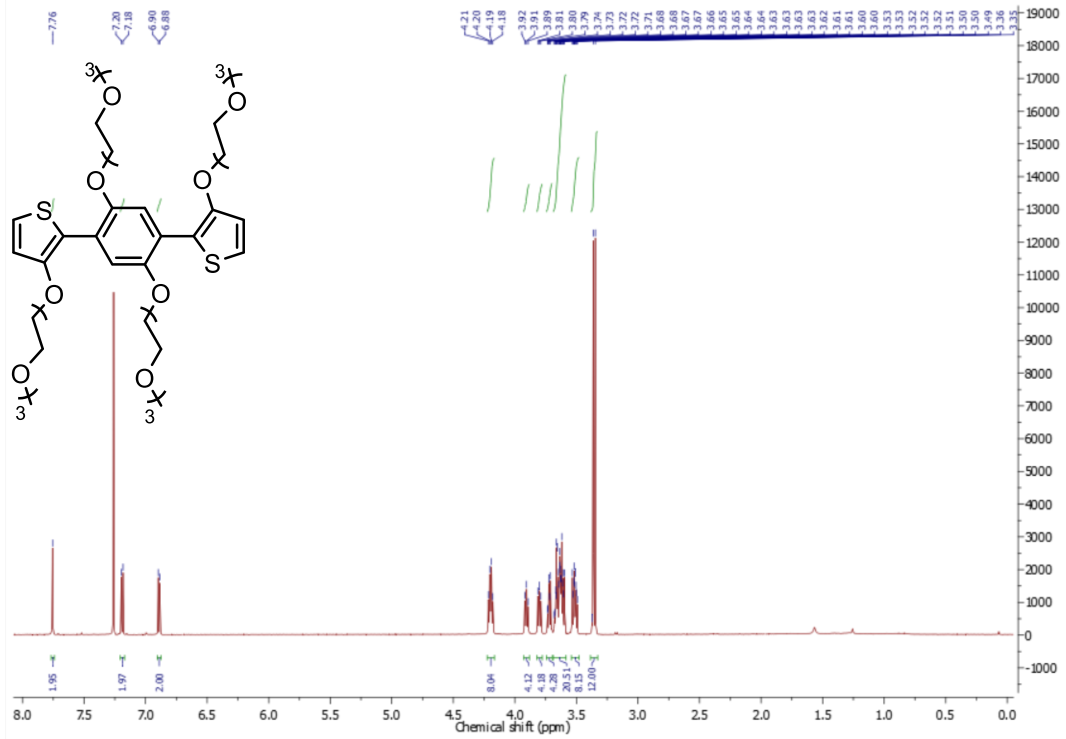

Figure S11: ^{1}H NMR spectrum of compound **12**

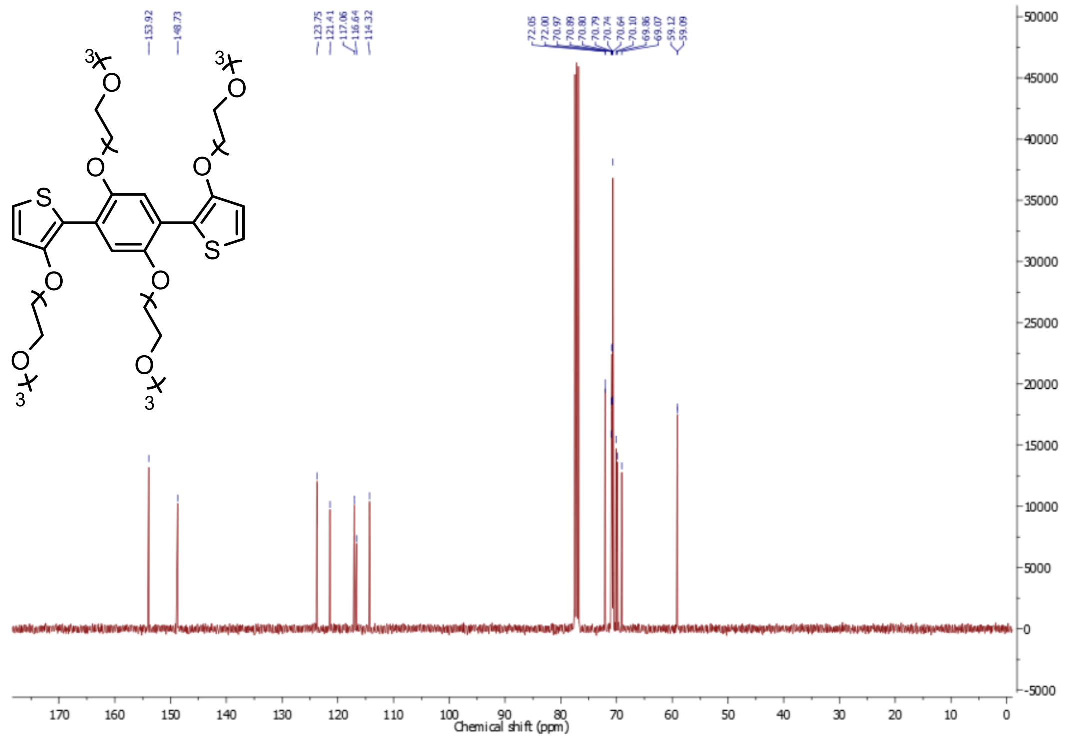

Figure S12: ^{13}C NMR spectrum of compound **12**

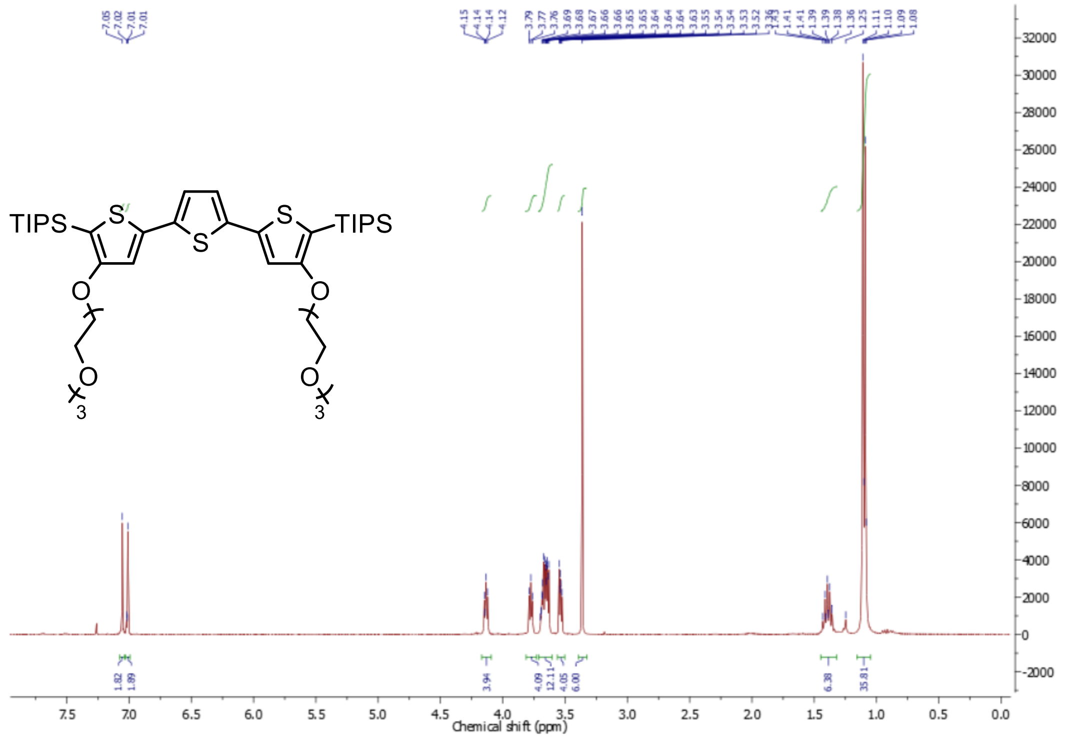

Figure S13: ^{1}H NMR spectrum of compound **13**

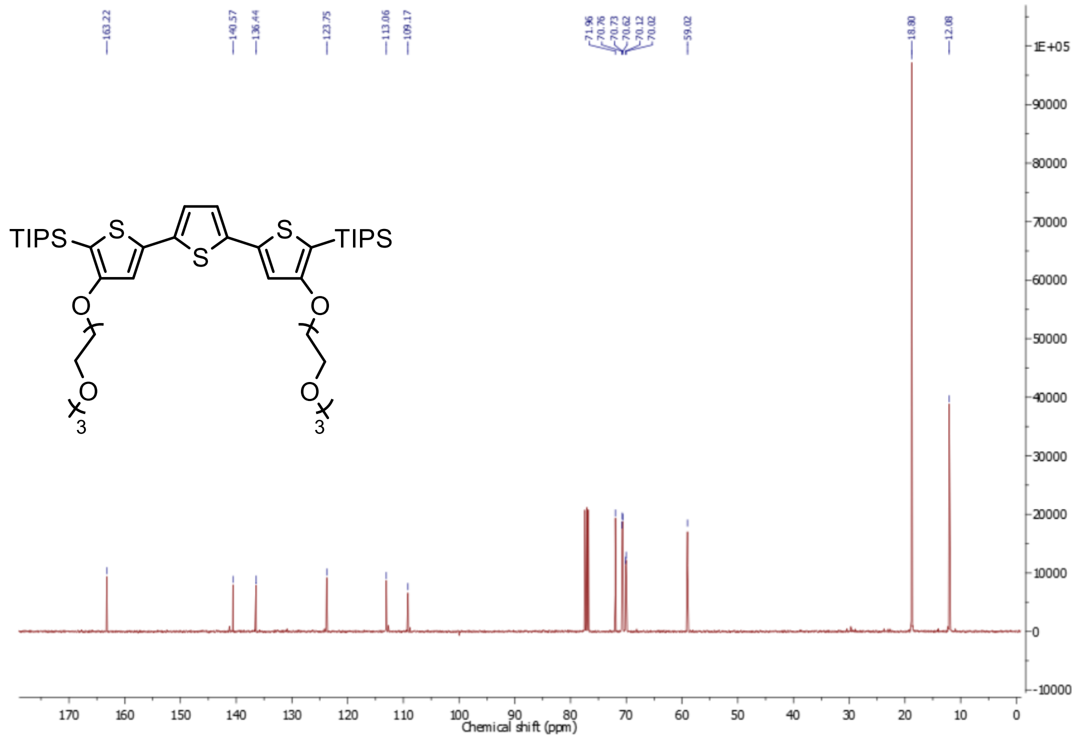

Figure S14: ^{13}C NMR spectrum of compound **13**

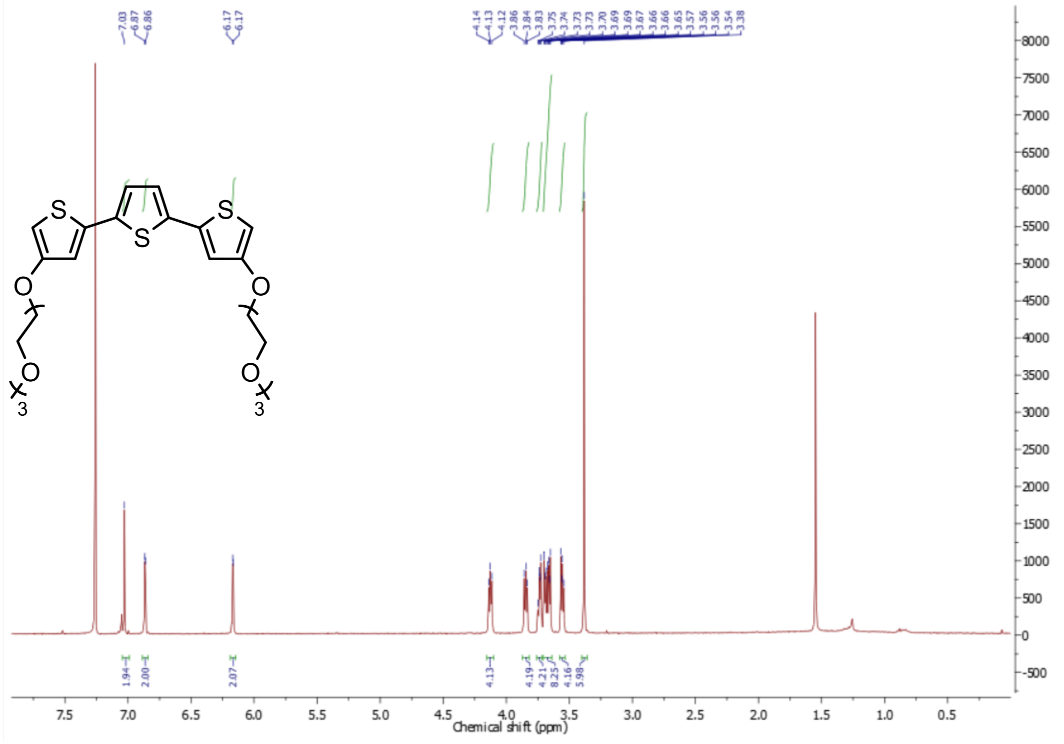

Figure S15: ^{1}H NMR spectrum of compound **15**

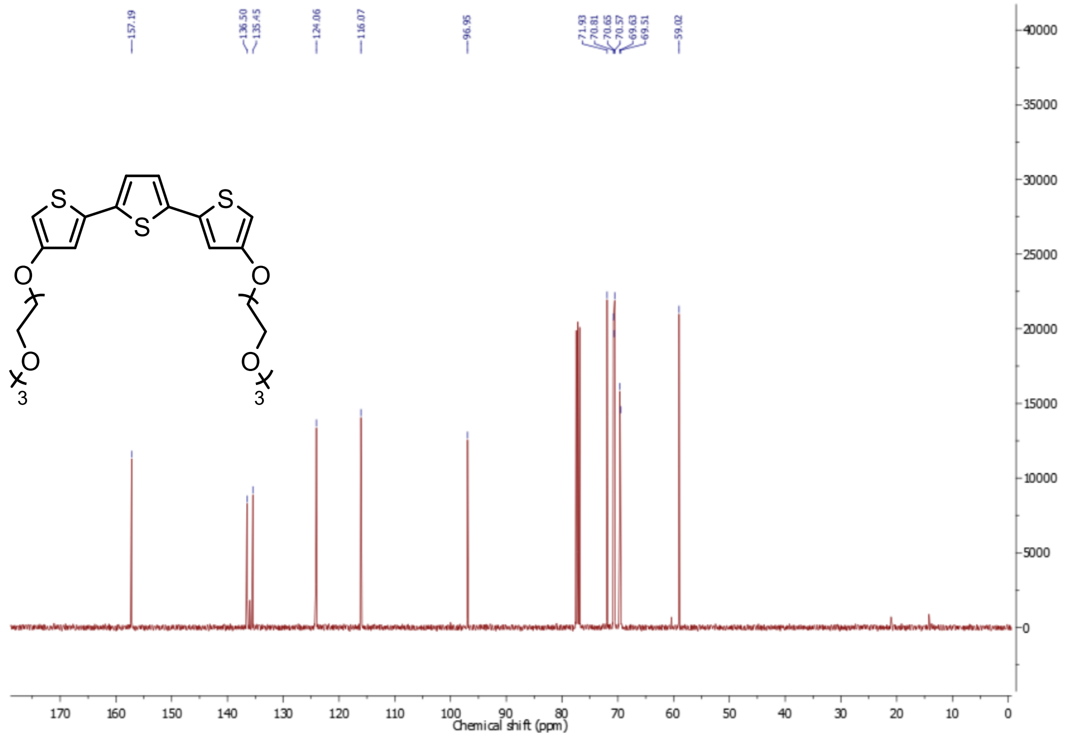

Figure S16: ^{13}C NMR spectrum of compound **15**

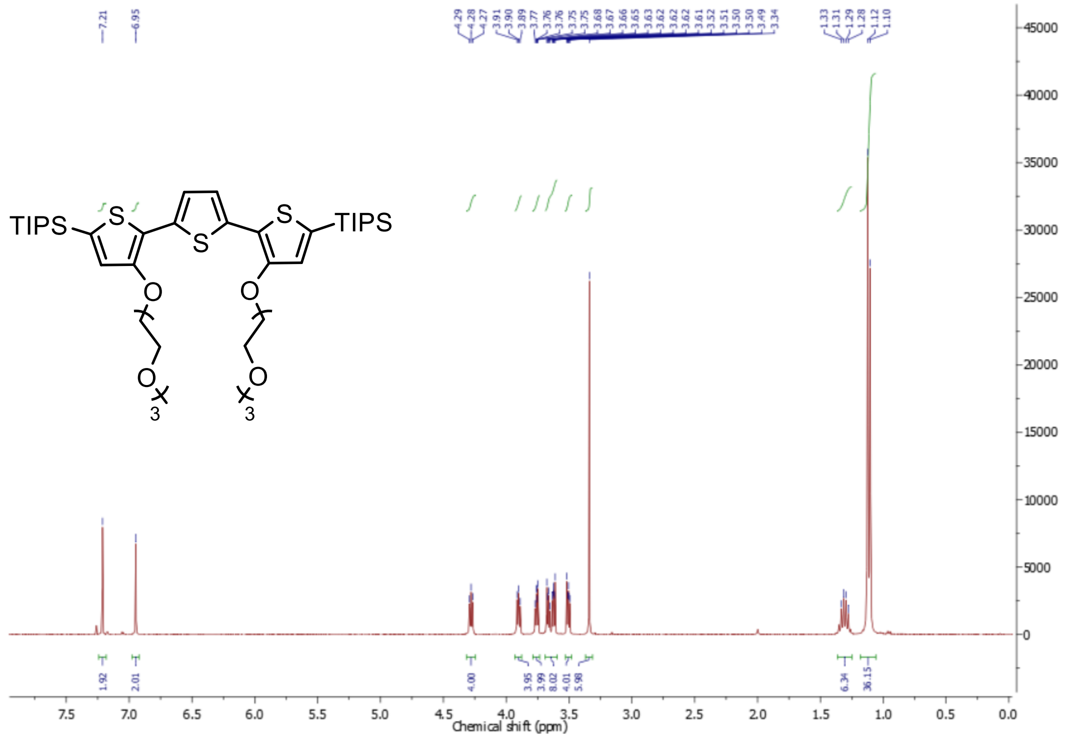

Figure S17: ^{1}H NMR spectrum of compound **14**

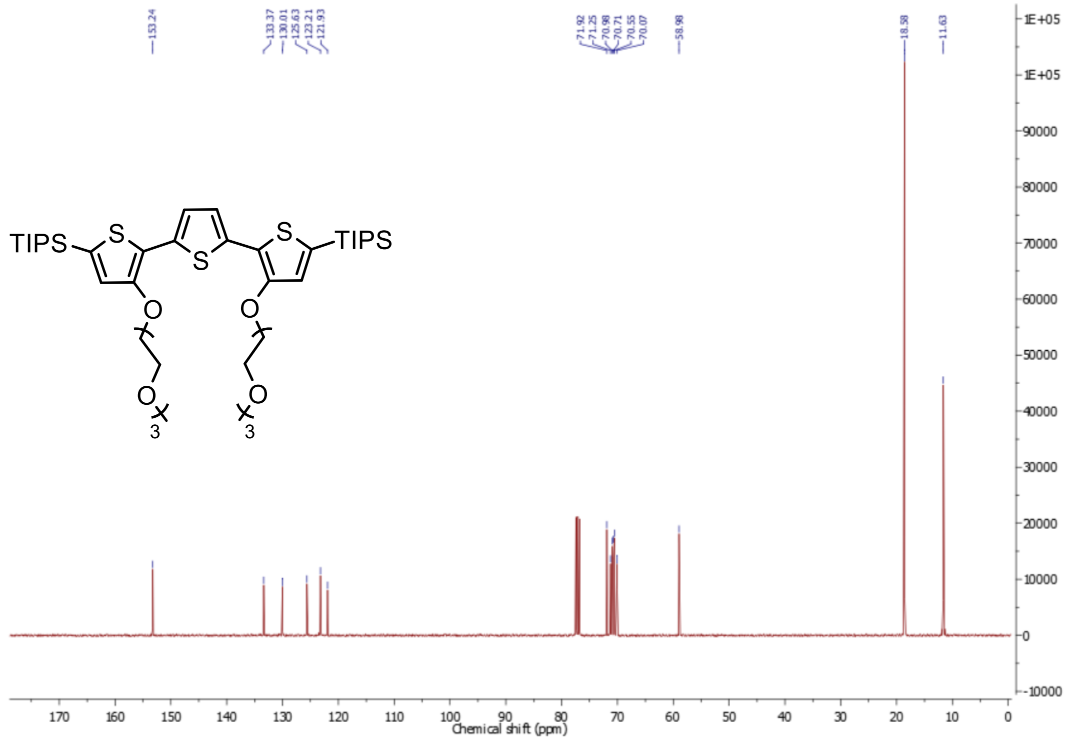

Figure S18: ^{13}C NMR spectrum of compound **14**

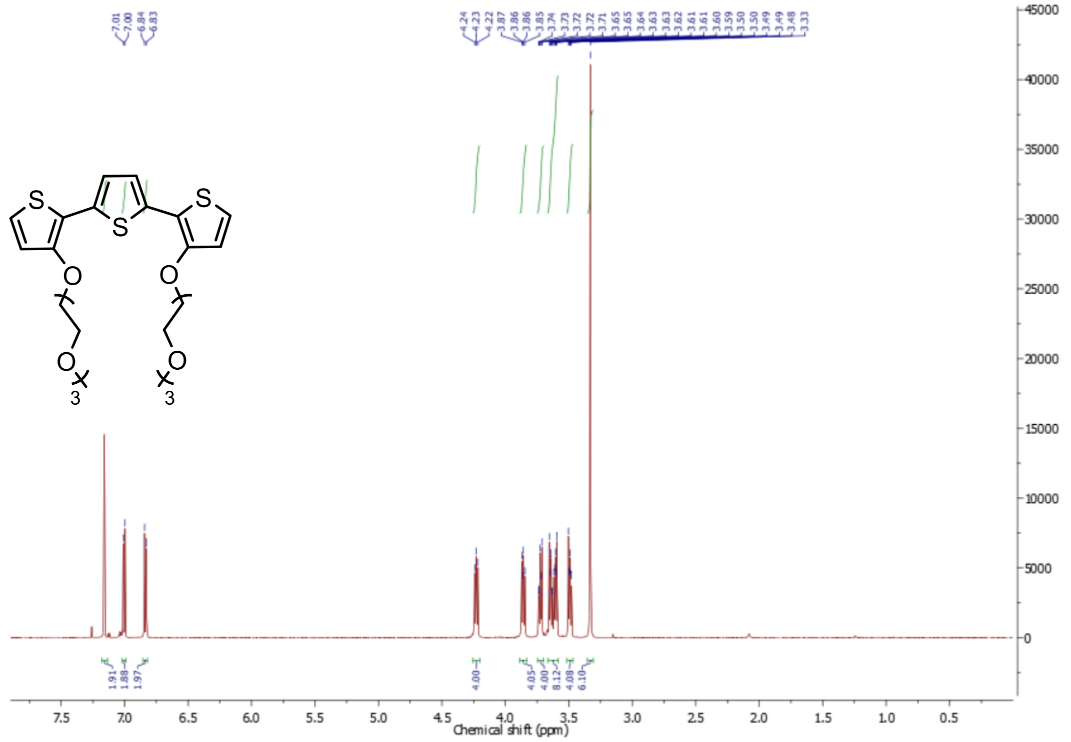

Figure S19: ^{1}H NMR spectrum of compound **16**

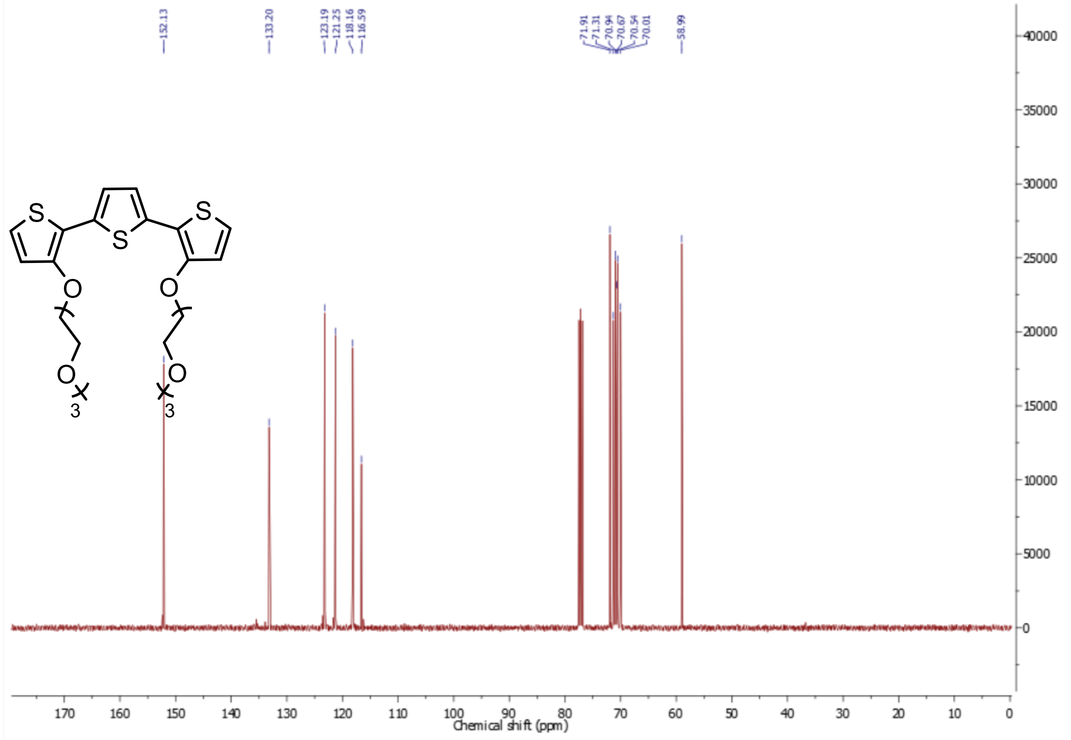

Figure S20: ^{13}C NMR spectrum of compound **16**

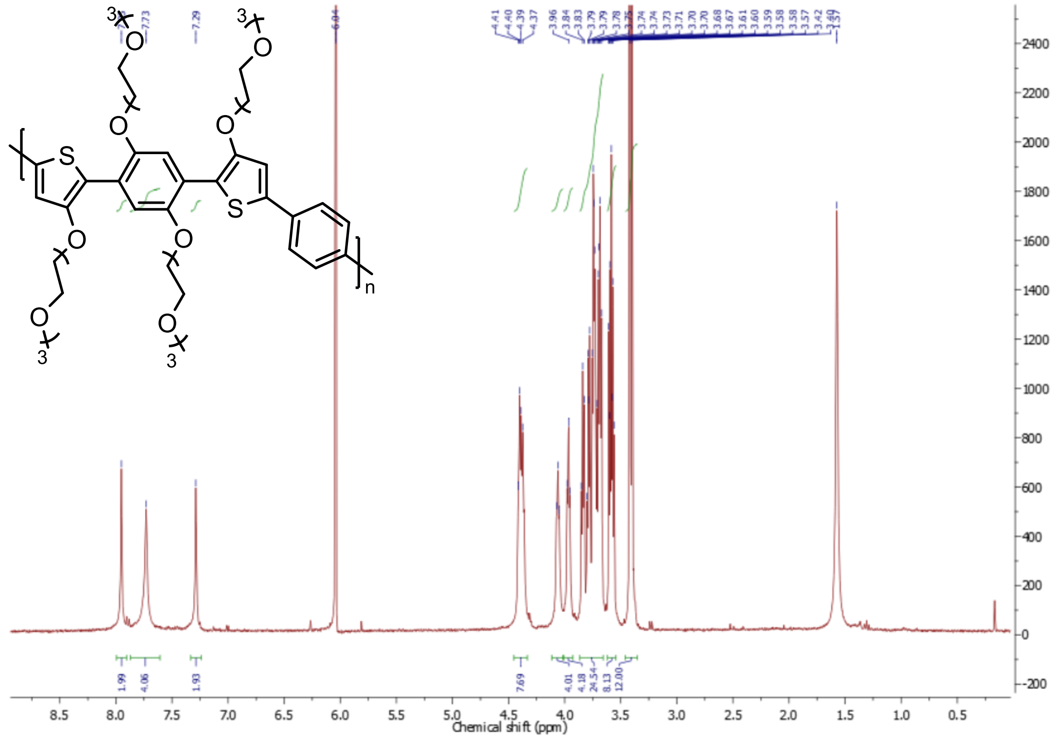

Figure S21: ^{1}H NMR spectrum of compound **inDTP-P**

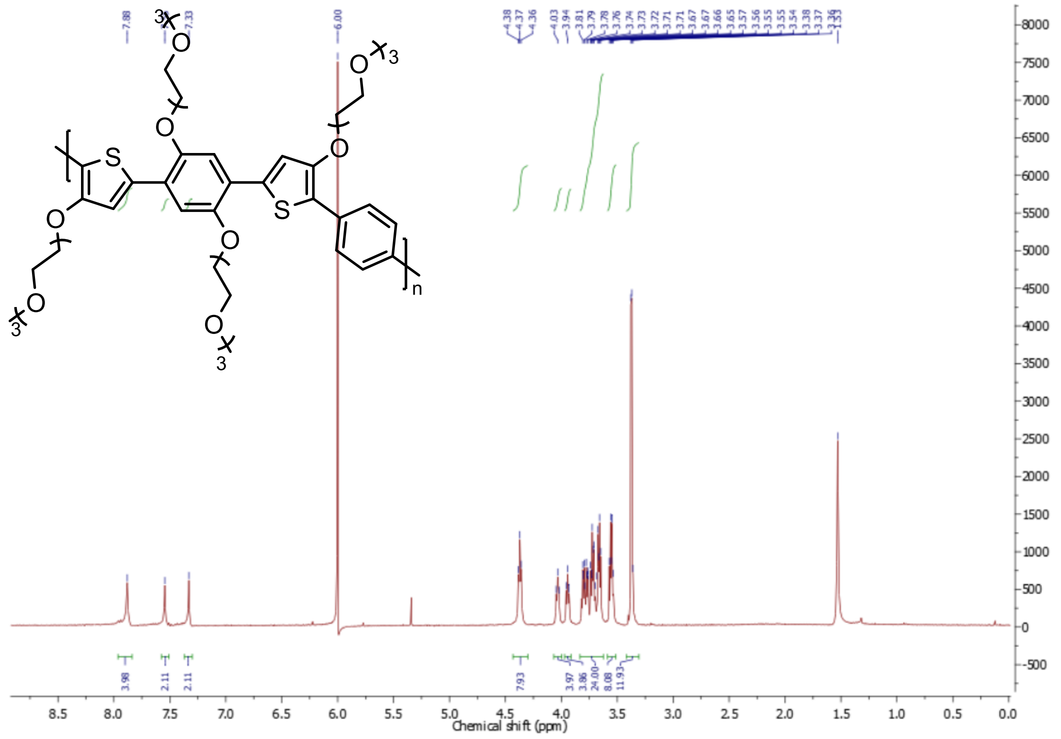

Figure S22: ^{1}H NMR spectrum of compound **outDTP-P**

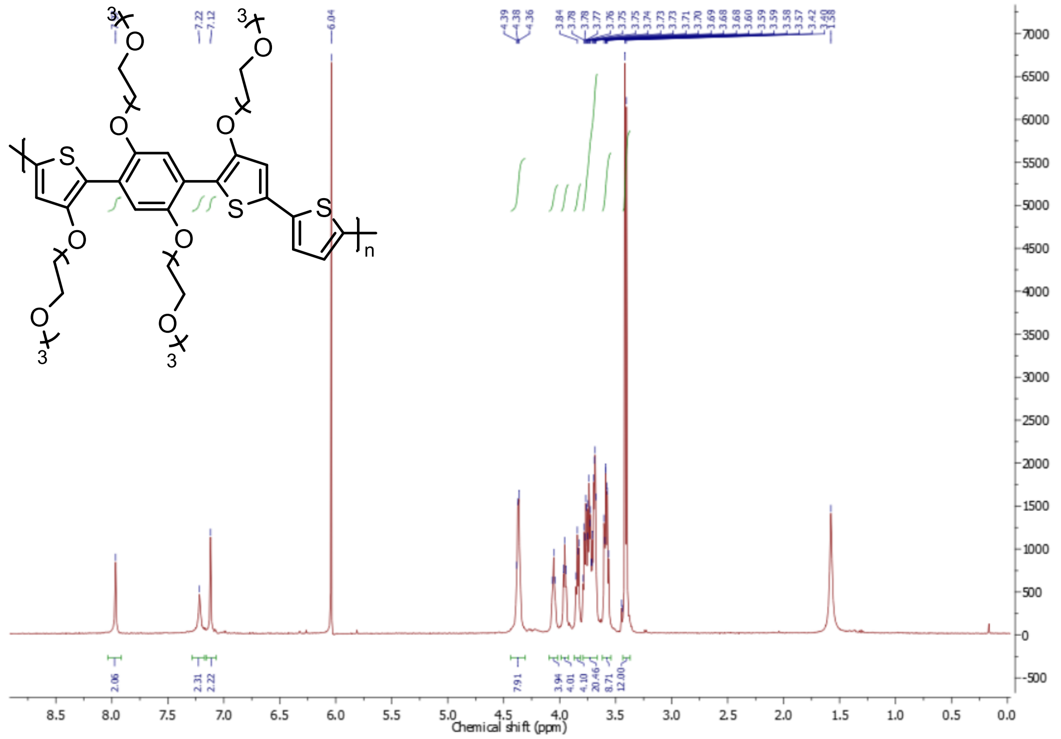

Figure S23: ^{1}H NMR spectrum of compound **inDTP-T**

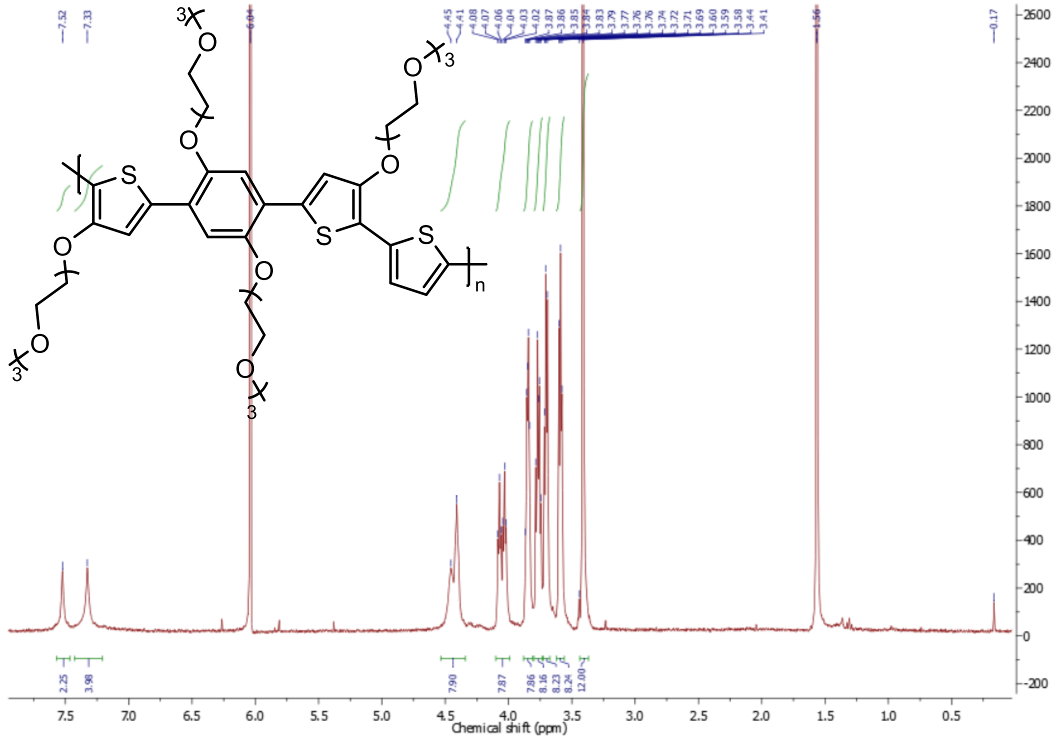

Figure S24: ^{1}H NMR spectrum of compound **outDTP-T**

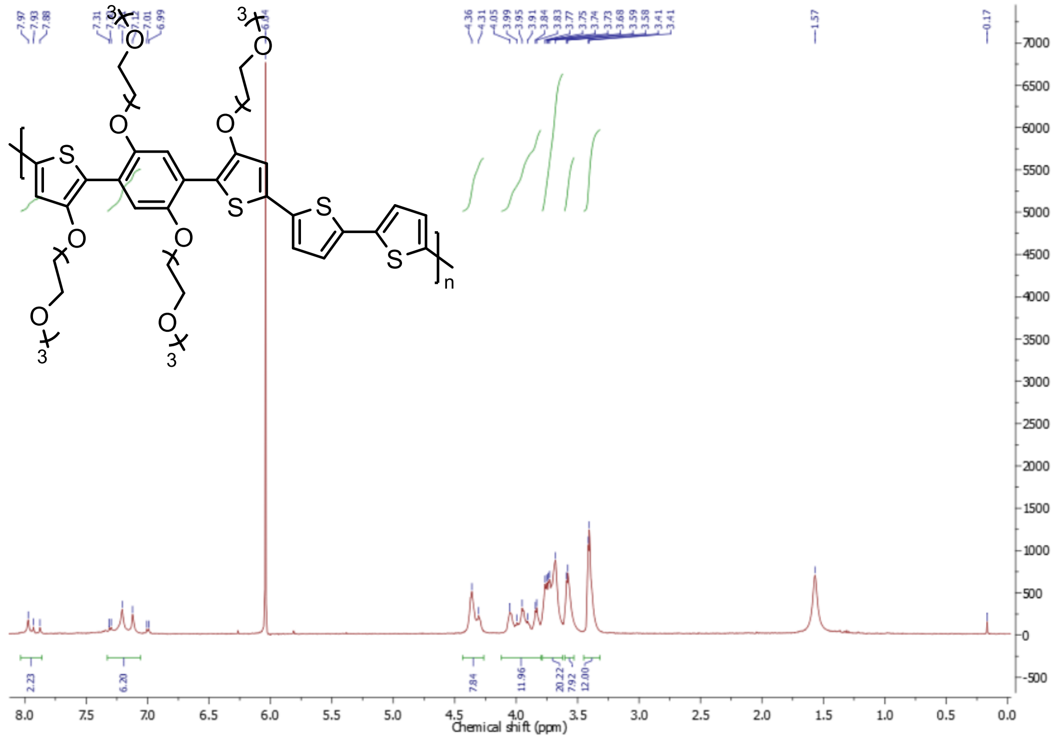

Figure S25: ^{1}H NMR spectrum of compound **inDTP-2T**

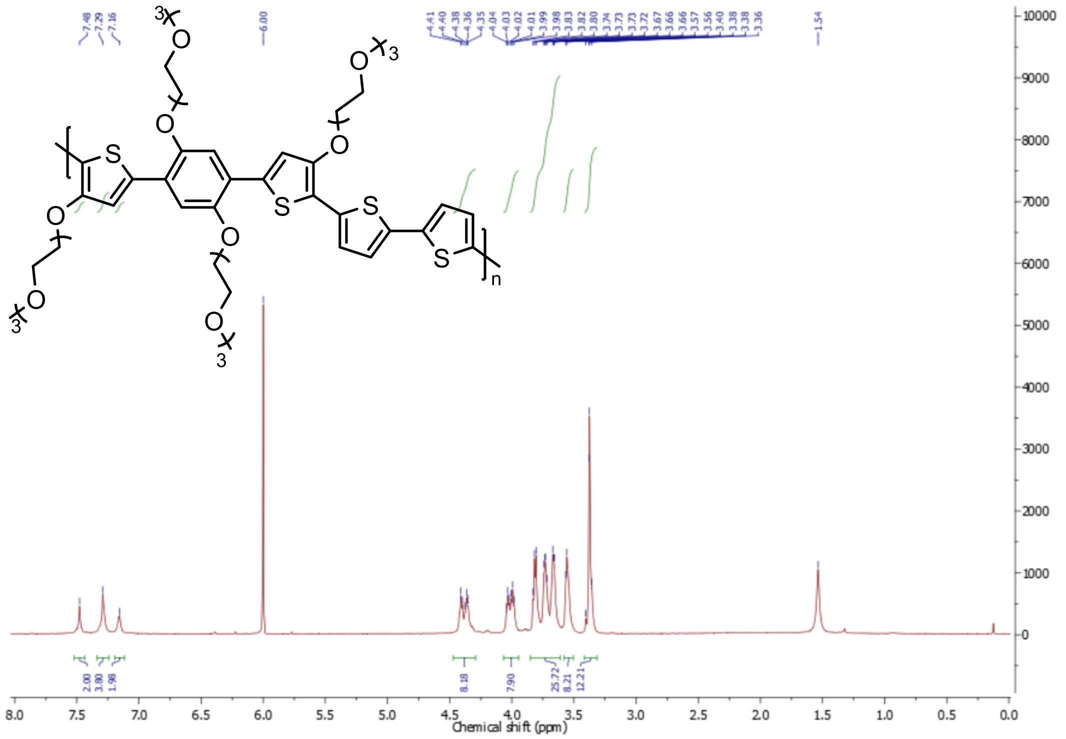

Figure S26: ^{1}H NMR spectrum of compound **outDTP-2T**

4 Gel permeation chromatography

Table S1a: Measured values of molecular weights in chlorobenzene

Polymer	M_n [g mol^{-1}]	M_W [g mol^{-1}]	PDI [-]
**inDTP-P**	12,000	17,000	1.36
**outDTP-P**	14,000	18,000	1.27
**inDTP-T**	8,500	9,900	1.16
**outDTP-T**	4,200	4,900	1.14
**inDTP-2T**	5,400	8,900	1.66
**outDTP-2T**	8,300	10,000	1.24

Table S2b: Measured values of molecular weights in N,N-dimethylformamide

Polymer	M_n [g mol^{-1}]	M_W [g mol^{-1}]	PDI [-]	DP
**inDTP-P**	46,000	158,000	3.43	48
**outDTP-P**	21,900	41,700	1.90	23
**inDTP-T**	27,800	62,900	2.26	29
**outDTP-T**	19,000	54,500	2.87	20
**inDTP-2T**	13,400	37,100	2.77	13
**outDTP-2T**	16,000	44,900	2.81	15

Degree of polymerisation (DP) values calculated from the M_n values and repeat unit molecular weights of 964 g/mol (DTP-P), 970 g/mol (DTP-T) and 1052 g/mol (DTP-2T).

5 Thermogravimetric analysis

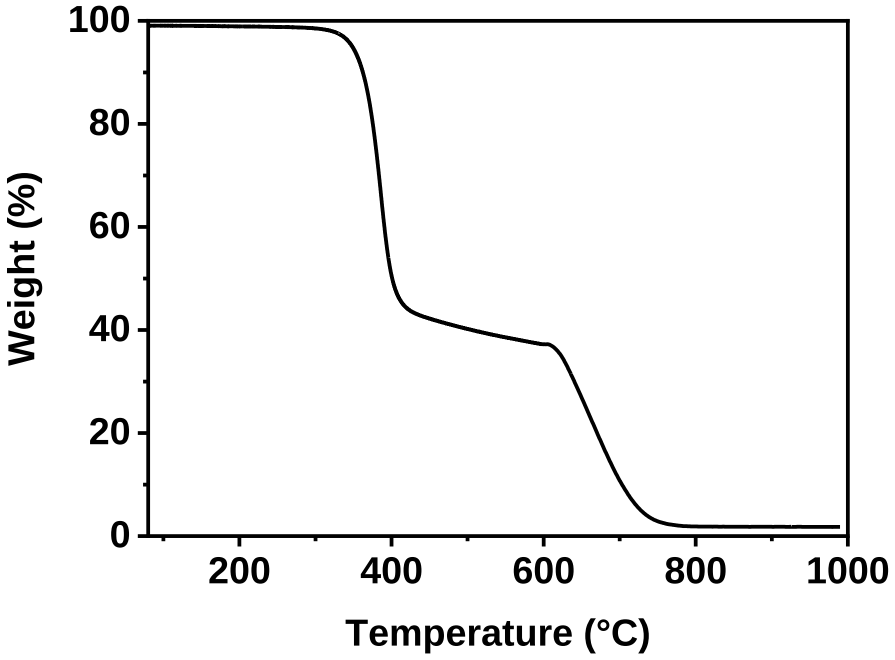

Figure S27: Thermogravimetric analysis of **inDTP-P** measured at heating rate 10 °C·min^{-1}

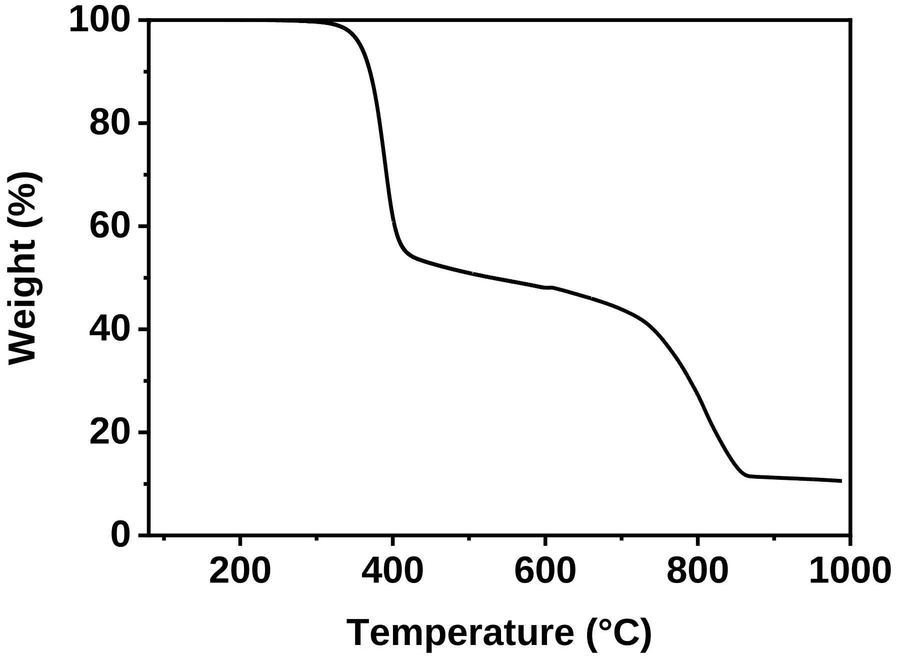

Figure S28: Thermogravimetric analysis of **outDTP-P** measured at heating rate 10 °C·min^{-1}

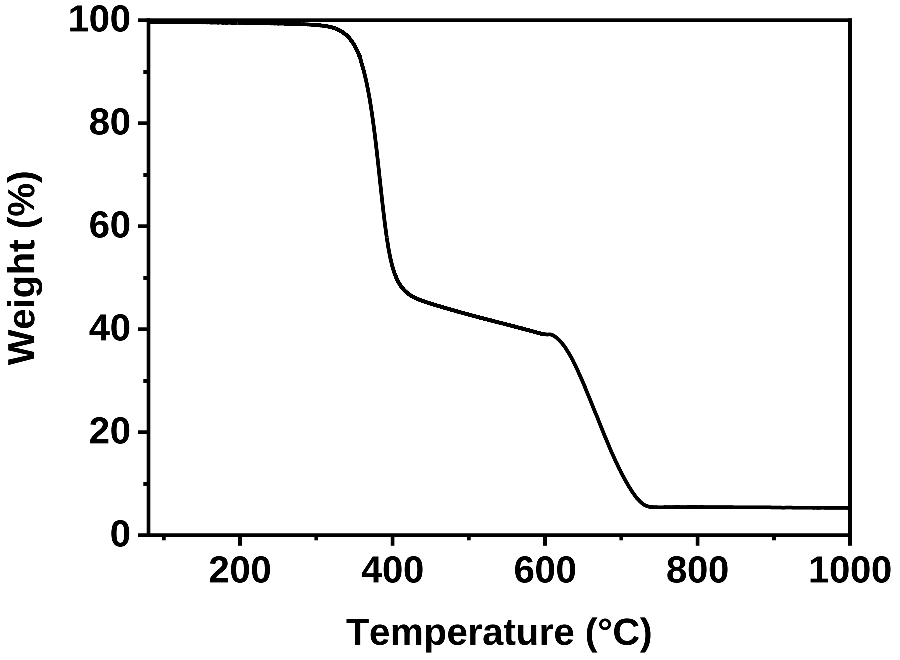

Figure S29: Thermogravimetric analysis of **inDTP-T** measured at heating rate 10 °C·min^{-1}

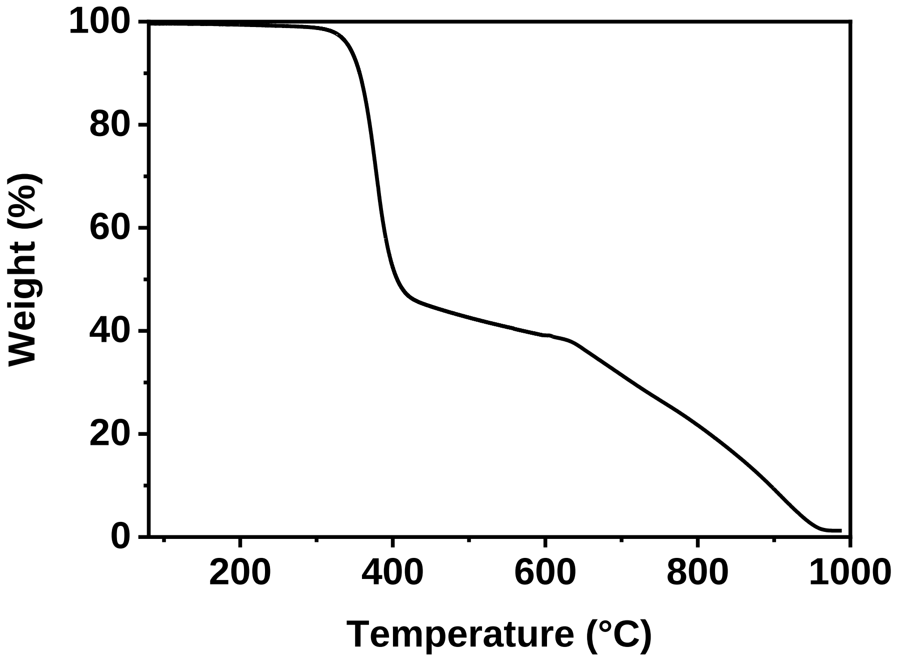

Figure S30: Thermogravimetric analysis of **outDTP-T** measured at heating rate 10 °C·min^{-1}

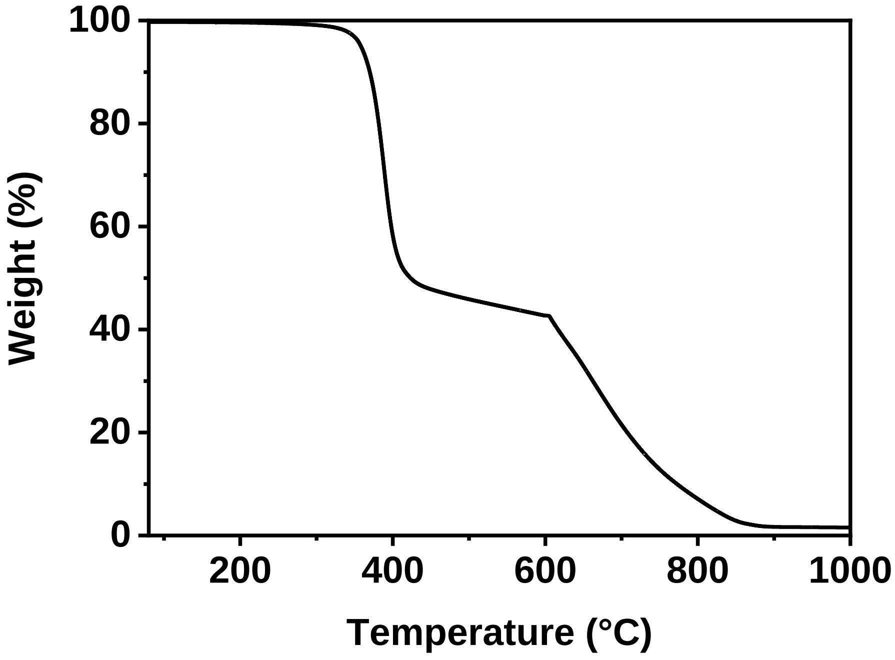

Figure S31: Thermogravimetric analysis of **inDTP-2T** measured at heating rate 10 °C·min^{-1}

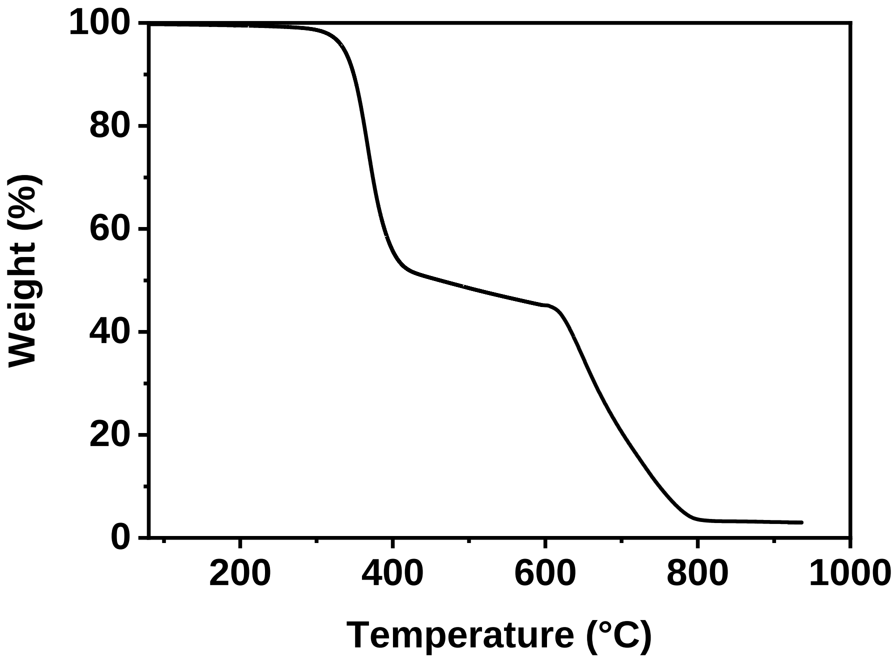

Figure S32: Thermogravimetric analysis of **outDTP-2T** measured at heating rate 10 °C·min^{-1}

Table S3: Summary of thermogravimetric analysis

Polymer	5% mass loss [°C]
**inDTP-P**	348
**outDTP-P**	358
**inDTP-T**	350
**outDTP-T**	343
**inDTP-2T**	358
**outDTP-2T**	335

6 Differential scanning calorimetry

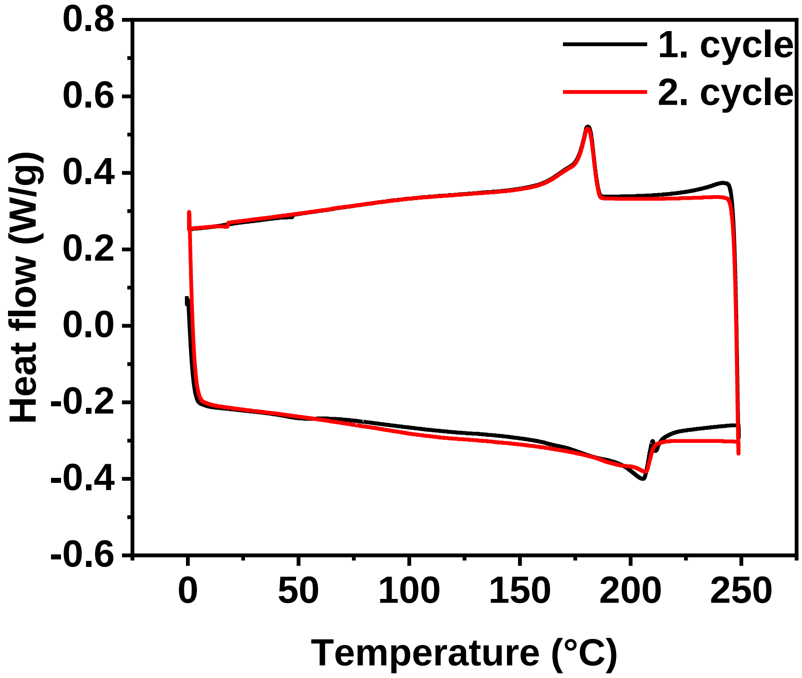

Figure S33: Differential scanning calorimetry of **inDTP-P** measured at heating rate 10 °C·min^{-1}

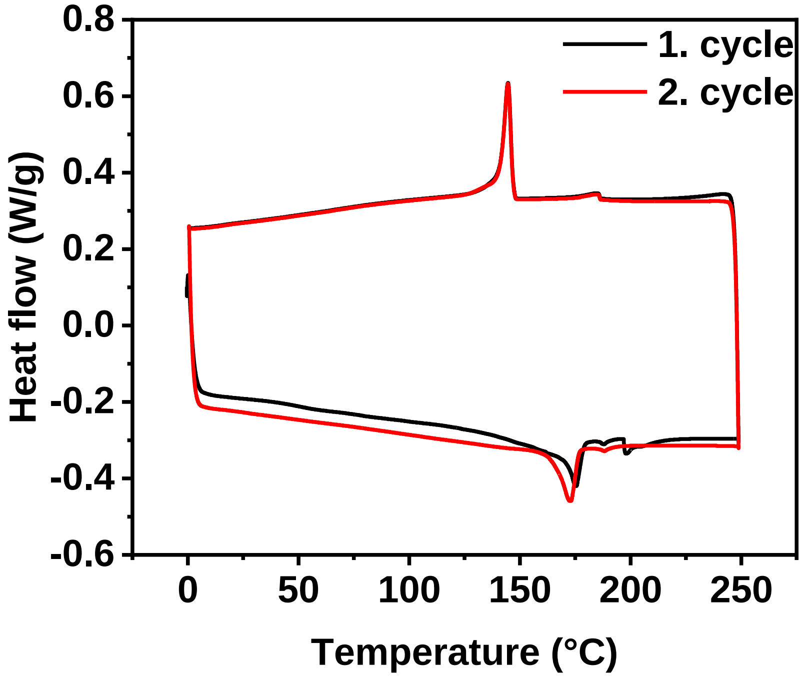

Figure S34: Differential scanning calorimetry of **outDTP-P** measured at heating rate 10 °C·min^{-1}

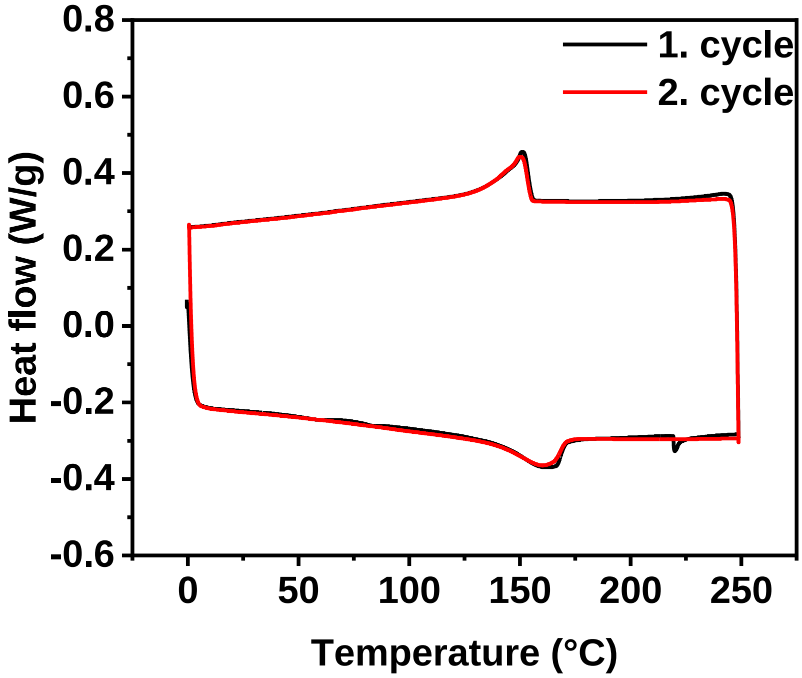

Figure S35: Differential scanning calorimetry of **inDTP-T** measured at heating rate 10 °C·min^{-1}

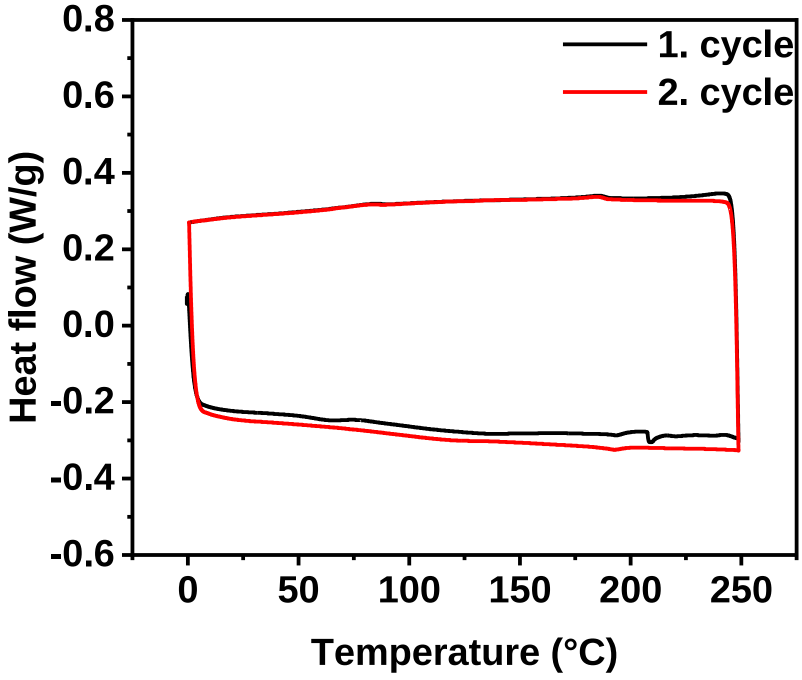

Figure S36: Differential scanning calorimetry of **outDTP-T** measured at heating rate 10 °C·min^{-1}

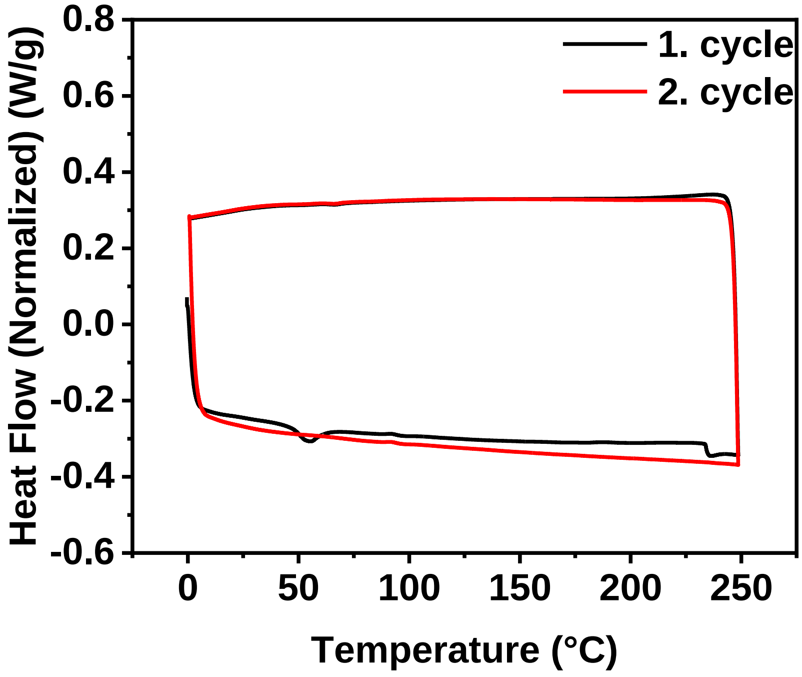

Figure S37: Differential scanning calorimetry of **inDTP-2T** measured at heating rate 10 °C·min^{-1}

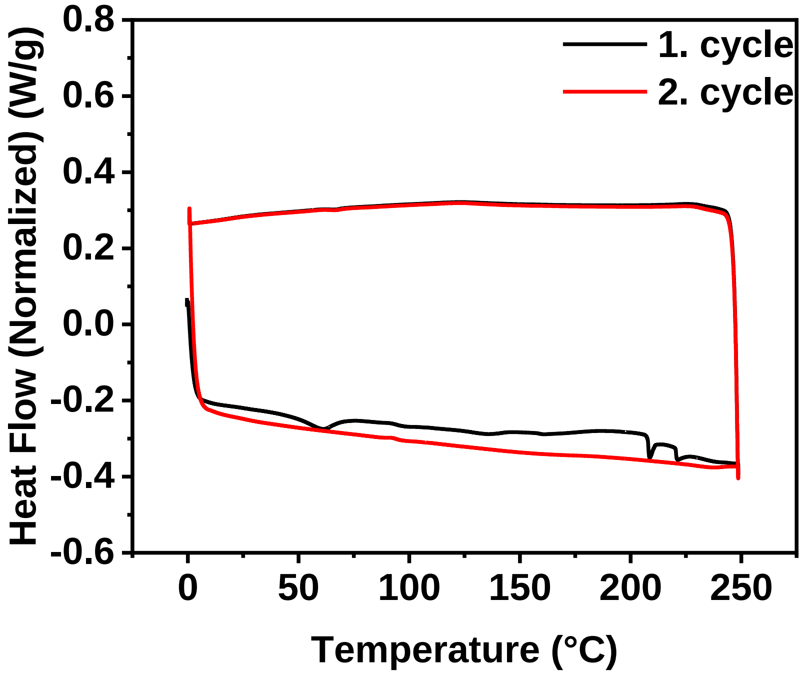

Figure S38: Differential scanning calorimetry of **outDTP-2T** measured at heating rate 10 °C·min^{-1}

7 Cyclic voltammetry

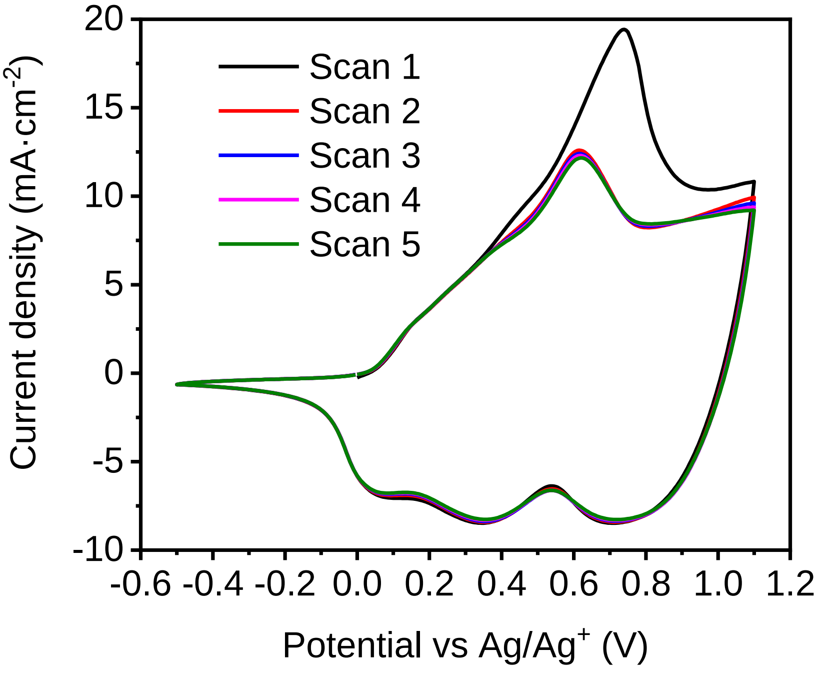

Figure S39: Cyclic voltammetry of the thin film of **inDTP-P** drop-cast from 5mg·ml^{-1} chloroform solution versus 0.1M (ACN) $TBAPF_6$ solution measured at 50 mV·s^{-1}, 5 mV step

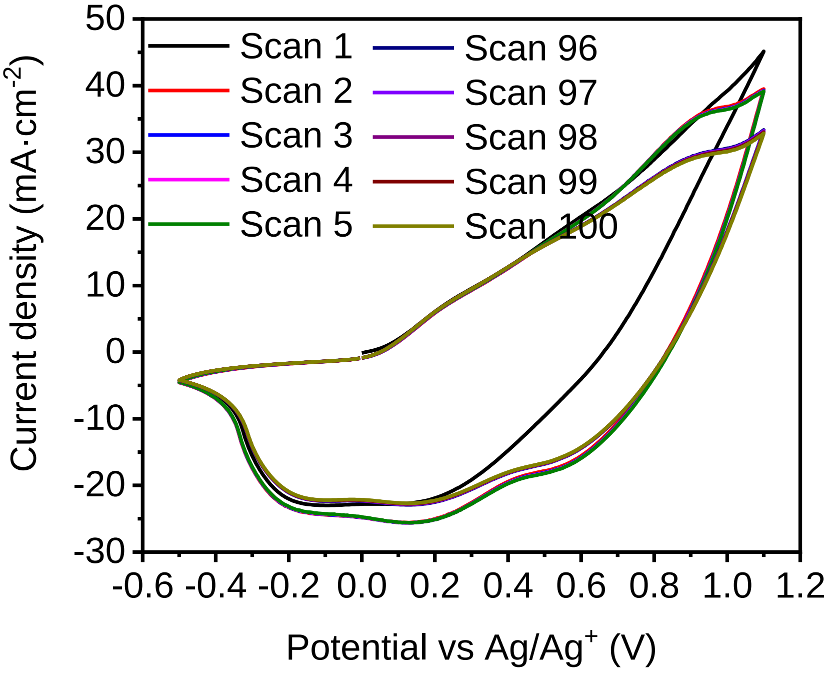

Figure S40: Cyclic voltammetry of the thin film of **inDTP-P** drop-cast from 5mg·ml^{-1} chloroform solution versus 0.1M (ACN) $TBAPF_6$ solution measured at 300 mV·s^{-1}, 5 mV step

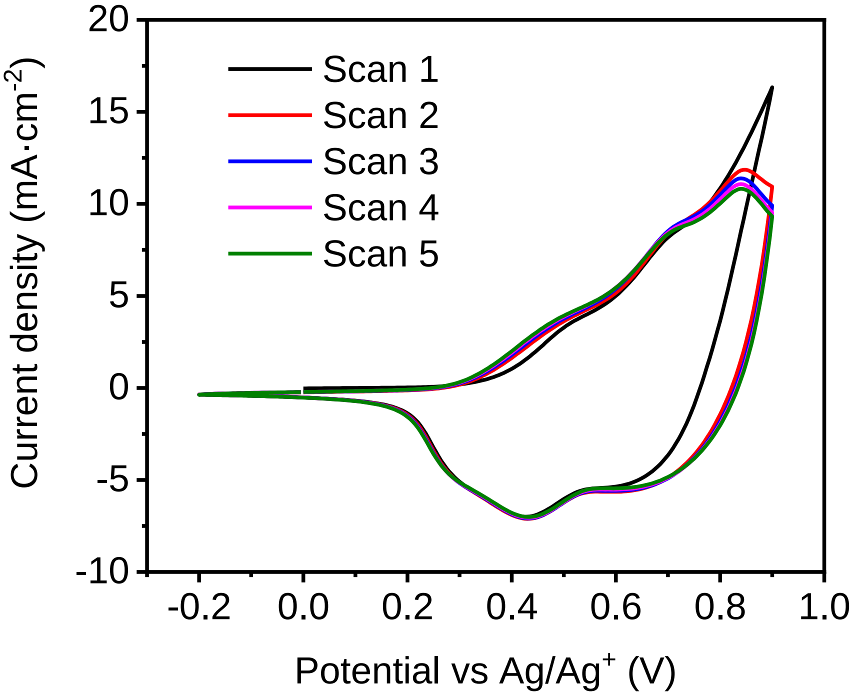

Figure S41: Cyclic voltammetry of the thin film of **inDTP-P** drop-cast from 5mg·ml^{-1} chloroform solution versus 0.1M (aq) NaCl solution measured at 50 mV·s^{-1}, 5 mV step

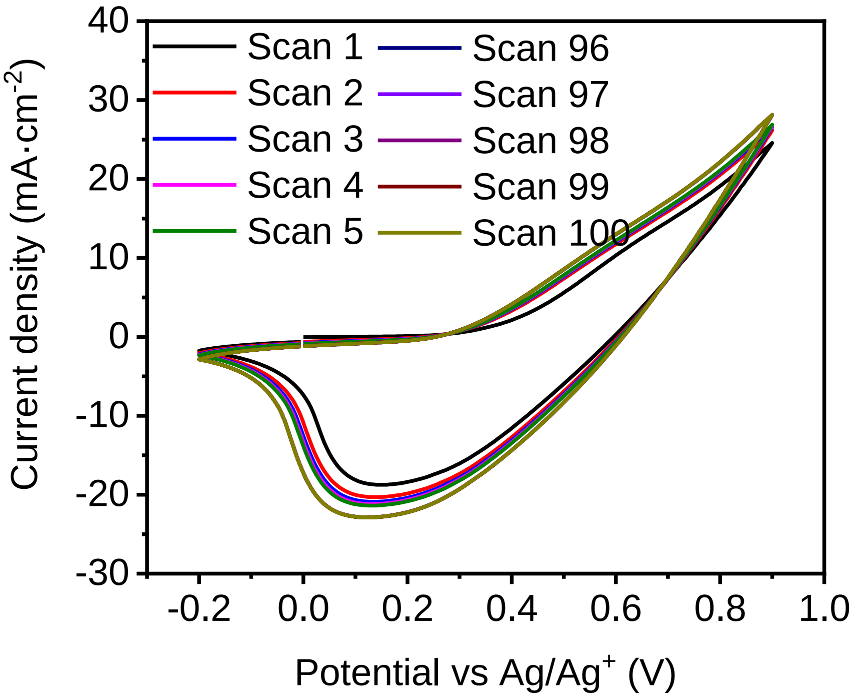

Figure S42: Cyclic voltammetry of the thin film of **inDTP-P** drop-cast from 5mg·ml^{-1} chloroform solution versus 0.1M (aq) NaCl solution measured at 300 mV·s^{-1}, 5 mV step

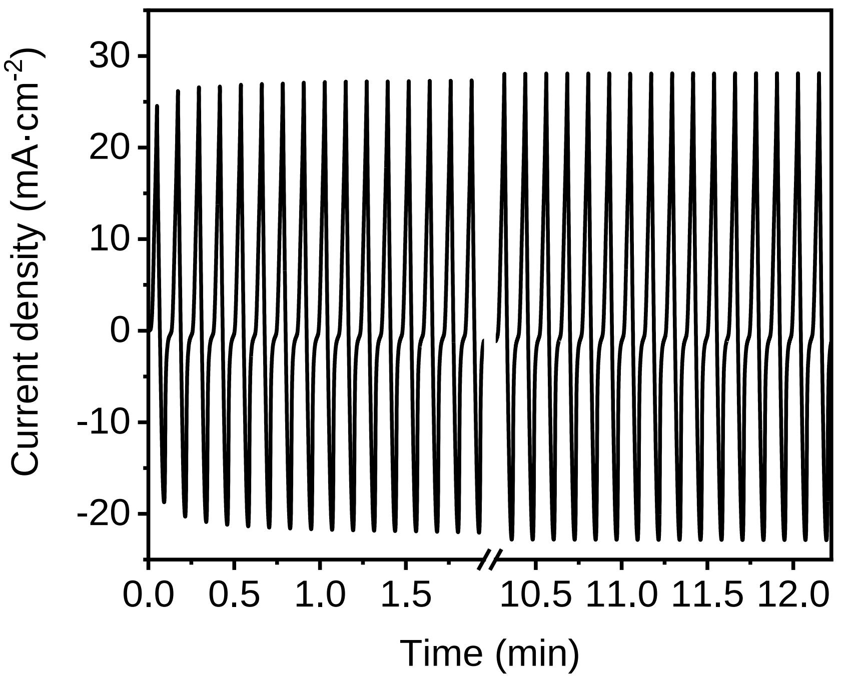

Figure S43: Stability measurement of **inDTP-P** using cyclic voltammetry of thin polymer film on the glassy carbon electrode drop-cast from chloroform solution (5 mg·ml^{-1}) with a scan rate of 300 mV·s^{-1} with 0.1 M NaCl as supporting electrolyte in water (100 scans), measured in the range of -0.2 – 0.9 V

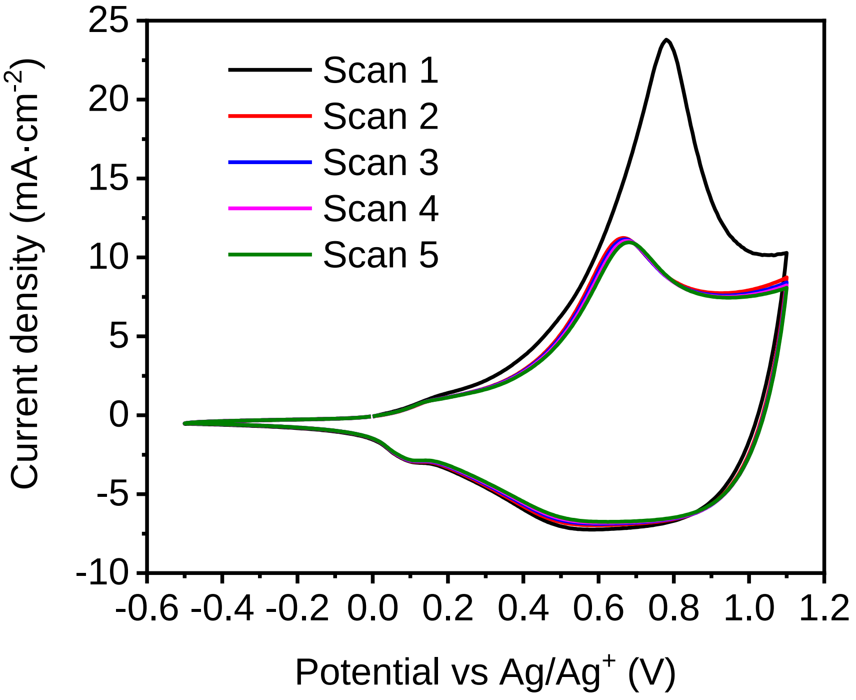

Figure S44: Cyclic voltammetry of the thin film of **outDTP-P** drop-cast from 5mg·ml^{-1} chloroform solution versus 0.1M (ACN) $TBAPF_6$ solution measured at 50 mV·s^{-1}, 5 mV step

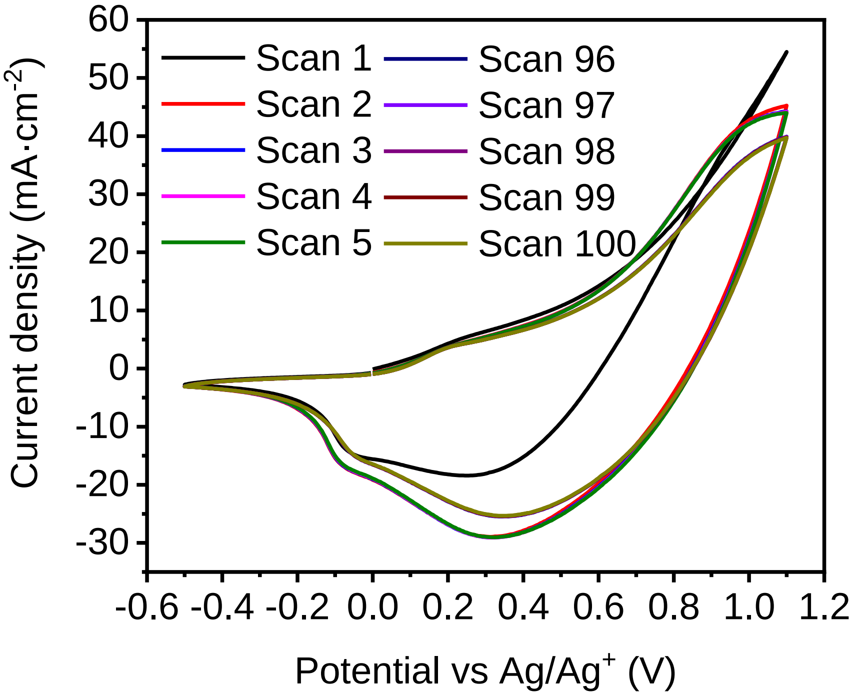

Figure S45: Cyclic voltammetry of the thin film of **outDTP-P** drop-cast from 5mg·ml^{-1} chloroform solution versus 0.1M (ACN) $TBAPF_6$ solution measured at 300 mV·s^{-1}, 5 mV step

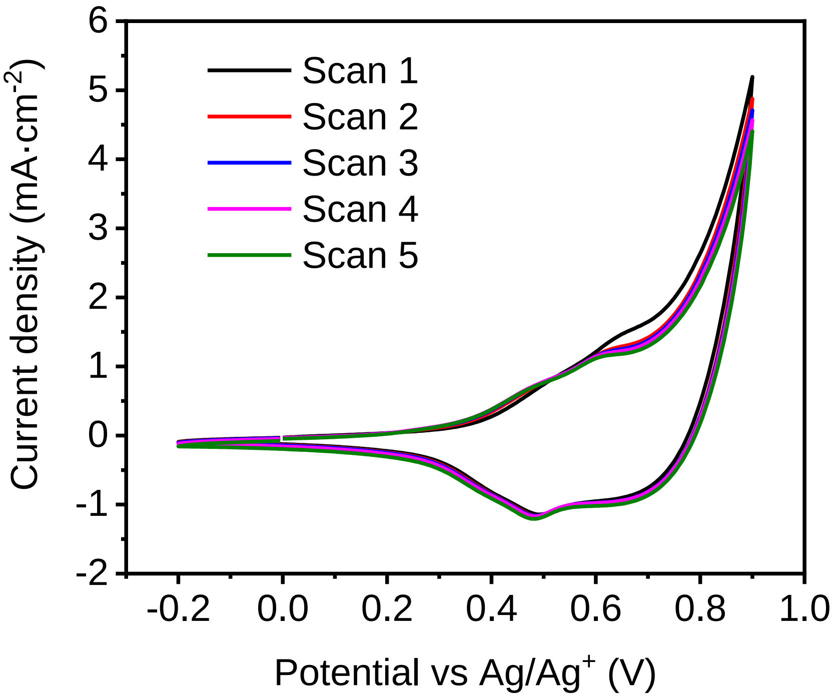

Figure S46: Cyclic voltammetry of the thin film of **outDTP-P** drop-cast from 5mg·ml^{-1} chloroform solution versus 0.1M (aq) NaCl solution measured at 50 mV·s^{-1}, 5 mV step

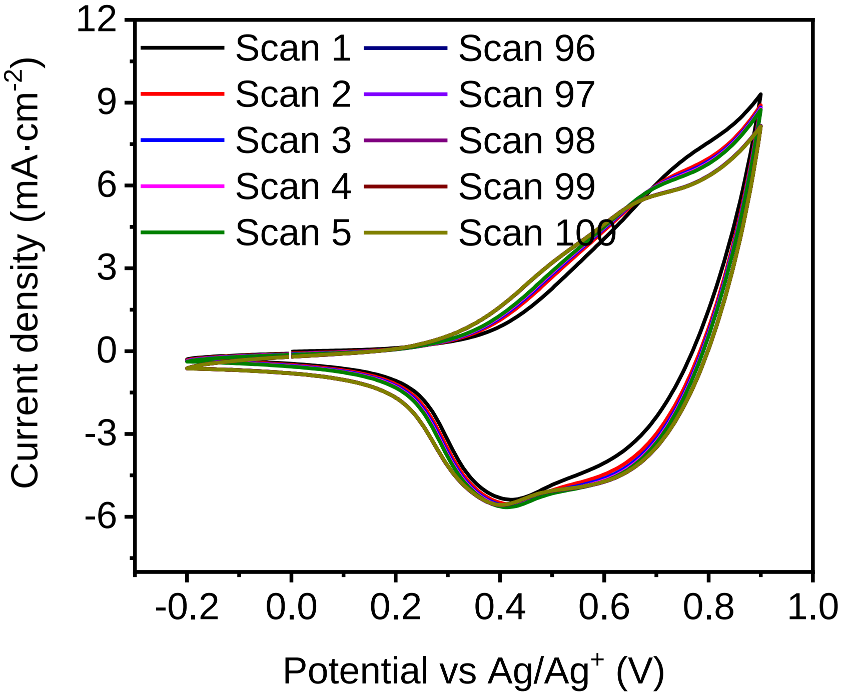

Figure S47: Cyclic voltammetry of the thin film of **outDTP-P** drop-cast from 5mg·ml^{-1} chloroform solution versus 0.1M (aq) NaCl solution measured at 300 mV·s^{-1}, 5 mV step

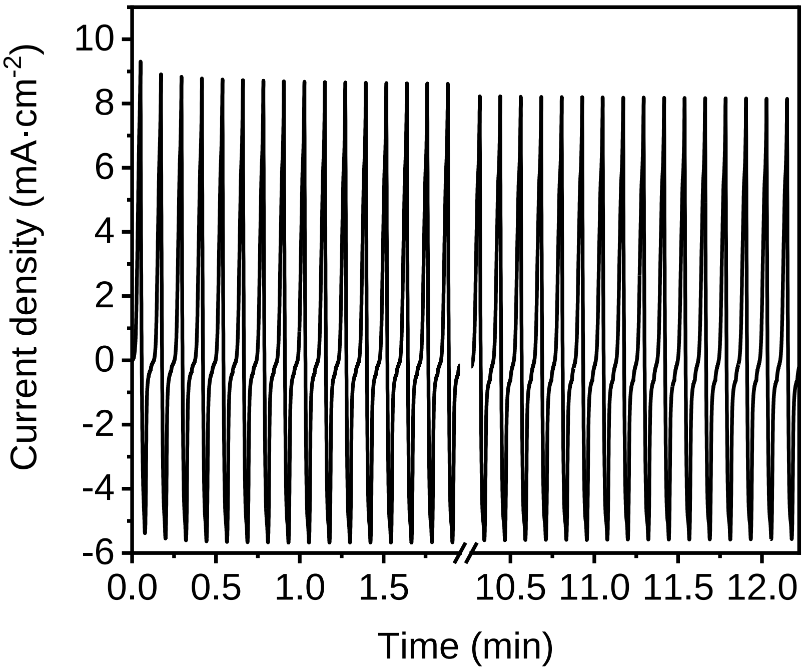

Figure S48: Stability measurement of **outDTP-P** using cyclic voltammetry of thin polymer film on the glassy carbon electrode drop-cast from chloroform solution (5 mg·ml^{-1}) with a scan rate of 300 mV·s^{-1} with 0.1 M NaCl as supporting electrolyte in water (100 scans), measured in the range of -0.2 – 0.9 V

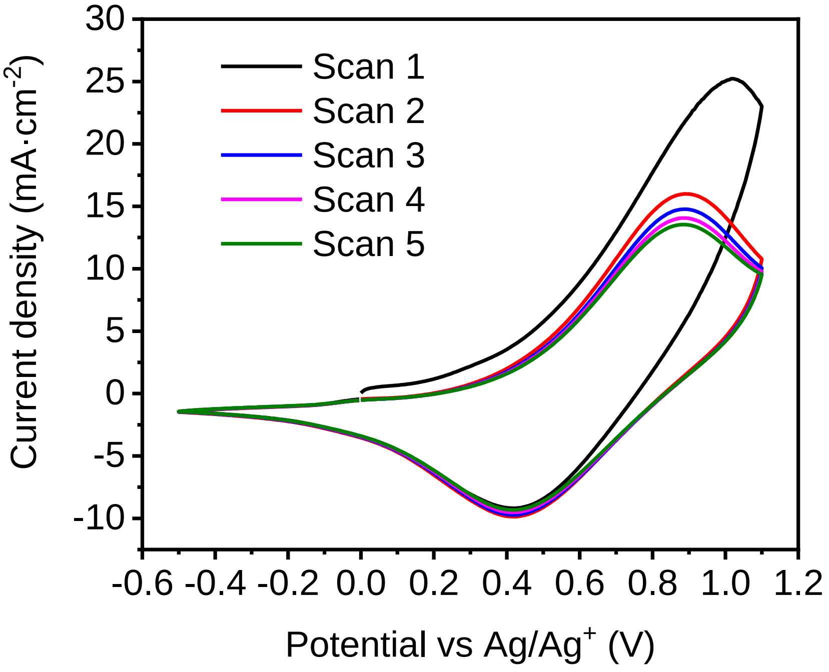

Figure S49: Cyclic voltammetry of the thin film of **inDTP-2T** drop-cast from 5mg·ml^{-1} chloroform solution versus 0.1M (ACN) $TBAPF_6$ solution measured at 50 mV·s^{-1}, 5 mV step

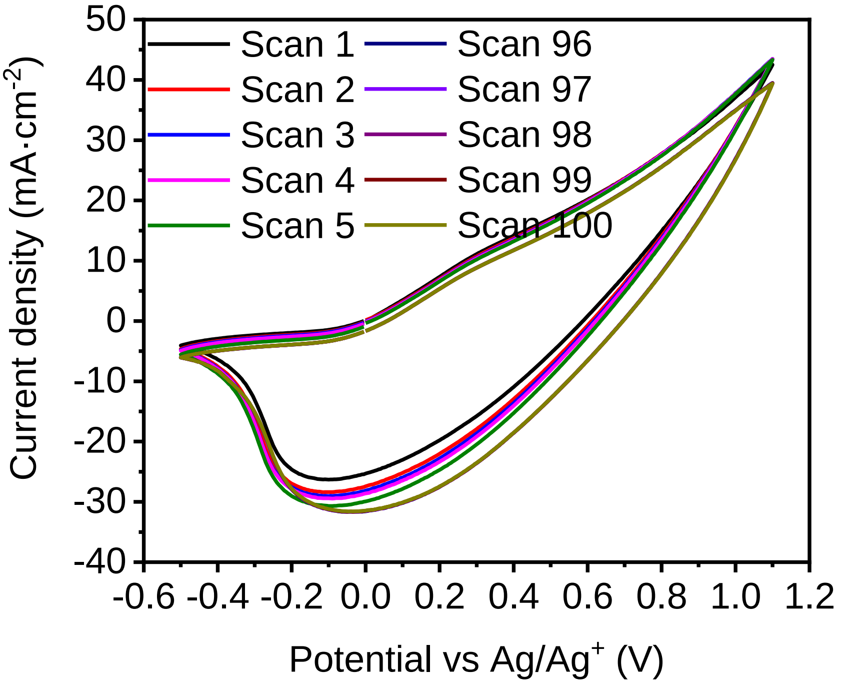

Figure S50: Cyclic voltammetry of the thin film of **inDTP-2T** drop-cast from 5mg·ml^{-1} chloroform solution versus 0.1M (ACN) $TBAPF_6$ solution measured at 300 mV·s^{-1}, 5 mV step

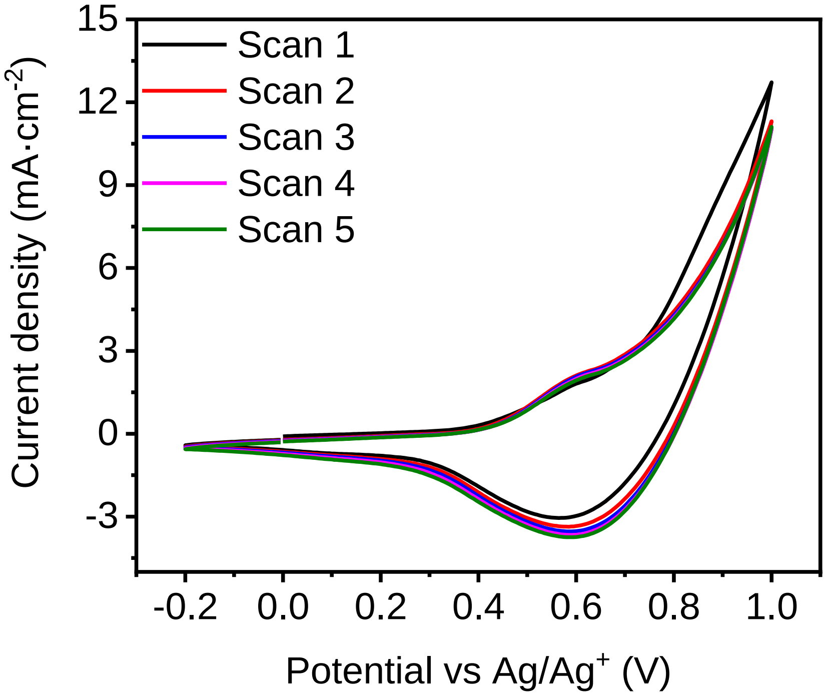

Figure S51: Cyclic voltammetry of the thin film of **inDTP-2T** drop-cast from 5mg·ml^{-1} chloroform solution versus 0.1M (aq) NaCl solution measured at 50 mV·s^{-1}, 5 mV step

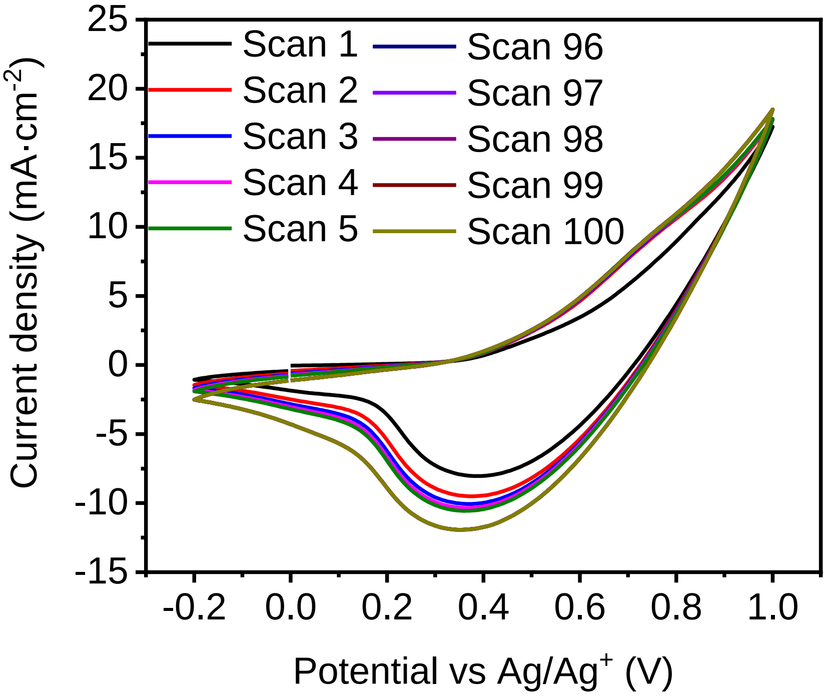

Figure S52: Cyclic voltammetry of the thin film of **inDTP-2T** drop-cast from 5mg·ml^{-1} chloroform solution versus 0.1M (aq) NaCl solution measured at 300 mV·s^{-1}, 5 mV step

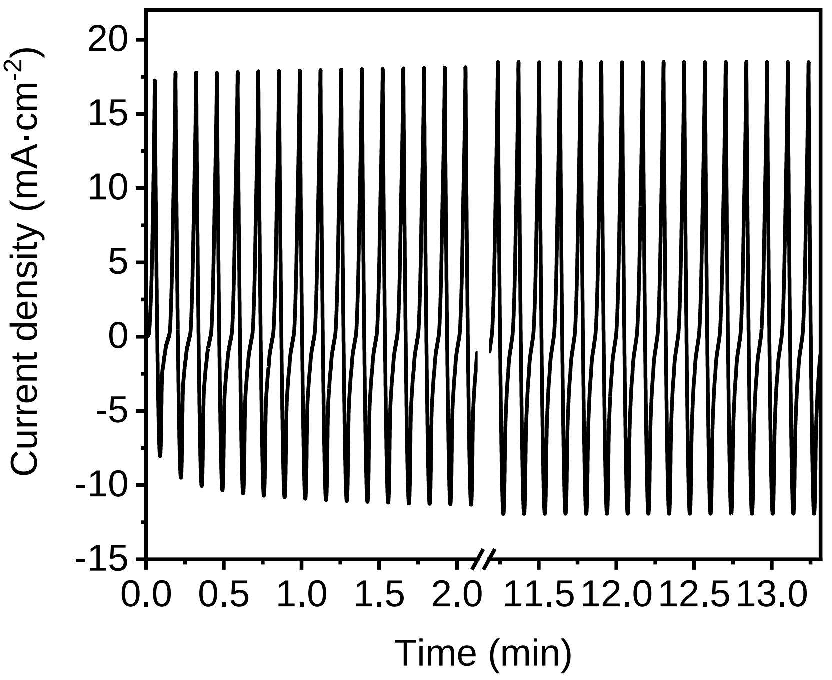

Figure S53: Stability measurement of **inDTP-2T** using cyclic voltammetry of thin polymer film on the glassy carbon electrode drop-cast from chloroform solution (5 mg·ml^{-1}) with a scan rate of 300 mV·s^{-1} with 0.1 M NaCl as supporting electrolyte in water (100 scans), measured in the range of -0.2 – 1.0 V

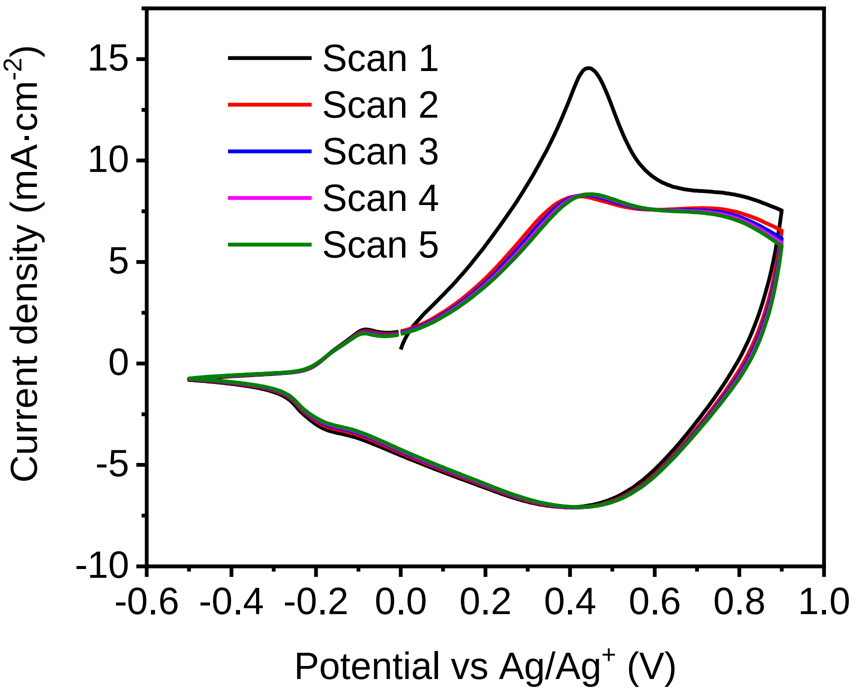

Figure S54: Cyclic voltammetry of the thin film of **outDTP-2T** drop-cast from 5mg·ml^{-1} chloroform solution versus 0.1M (ACN) $TBAPF_6$ solution measured at 50 mV·s^{-1}, 5 mV step

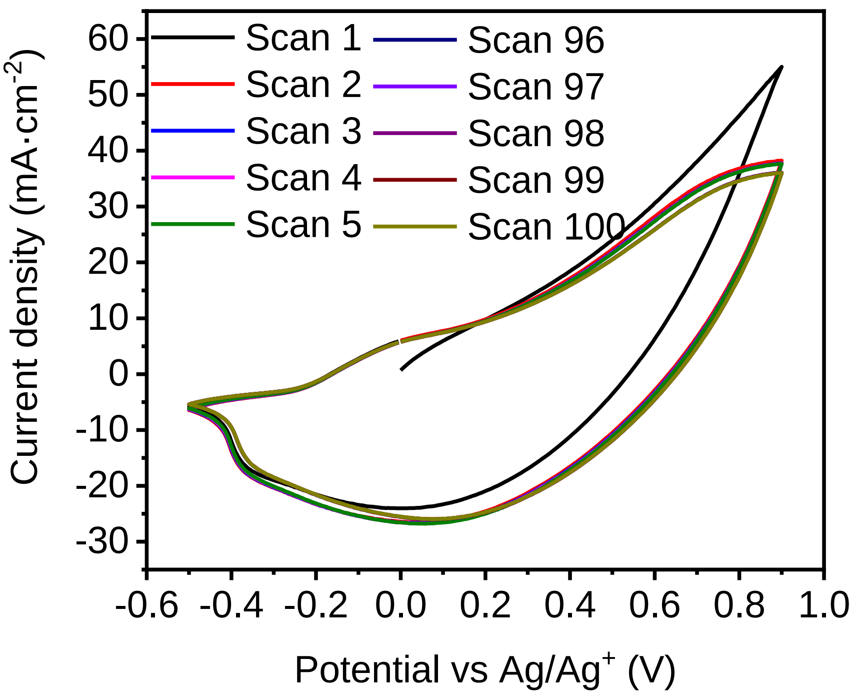

Figure S55: Cyclic voltammetry of the thin film of **outDTP-2T** drop-cast from 5mg·ml^{-1} chloroform solution versus 0.1M (ACN) $TBAPF_6$ solution measured at 300 mV·s^{-1}, 5 mV step

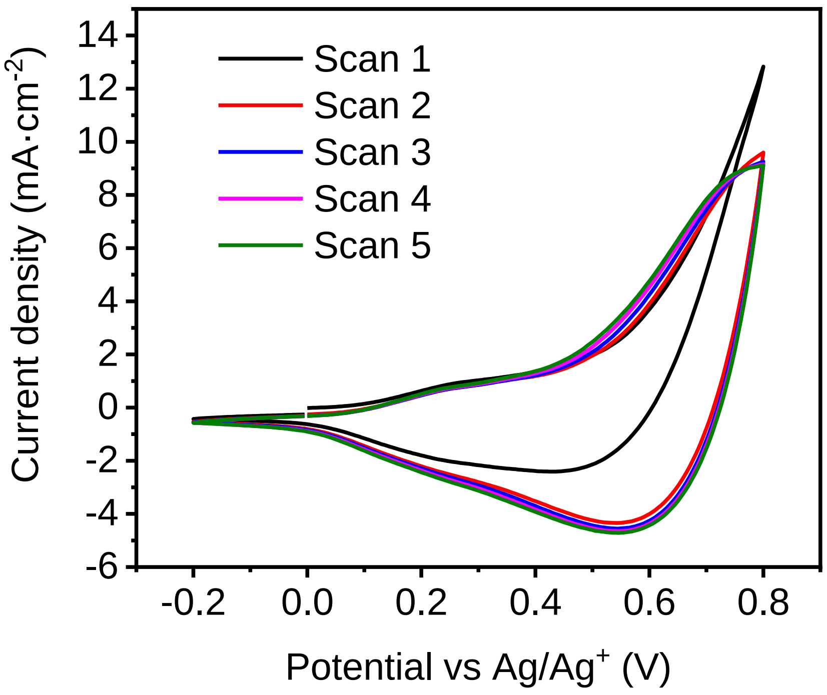

Figure S56: Cyclic voltammetry of the thin film of **outDTP-2T** drop-cast from 5mg·ml^{-1} chloroform solution versus 0.1M (aq) NaCl solution measured at 50 mV·s^{-1}, 5 mV step

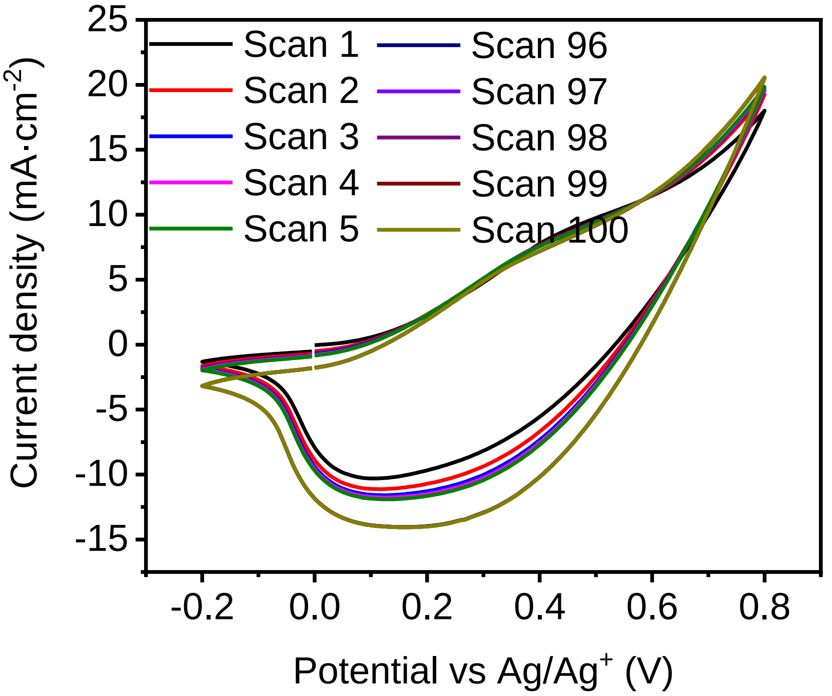

Figure S57: Cyclic voltammetry of the thin film of **outDTP-2T** drop-cast from 5mg·ml^{-1} chloroform solution versus 0.1M (aq) NaCl solution measured at 300 mV·s^{-1}, 5 mV step

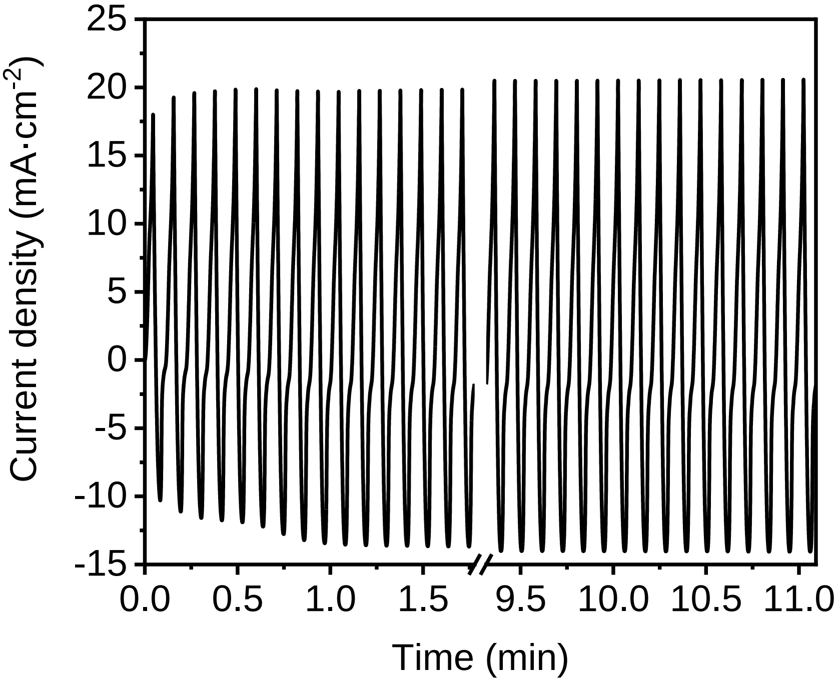

Figure S58: Stability measurement of **outDTP-2T** using cyclic voltammetry of thin polymer film on the glassy carbon electrode drop-cast from chloroform solution (5 mg·ml^{-1}) with a scan rate of 300 mV·s^{-1} with 0.1 M NaCl as supporting electrolyte in water (100 scans), measured in the range of -0.2 – 0.8 V

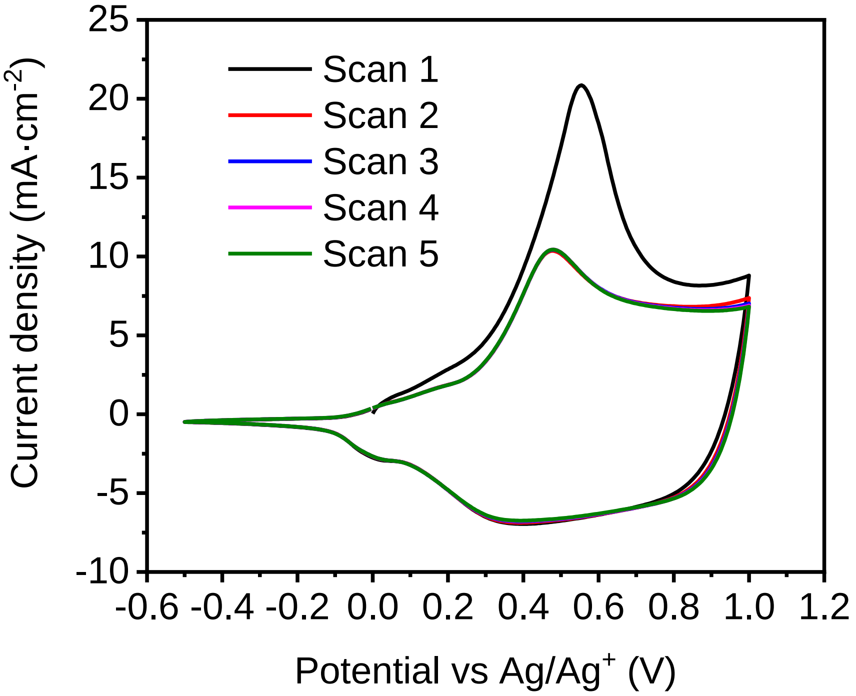

Figure S59: Cyclic voltammetry of the thin film of **inDTP-T** drop-cast from 5mg·ml^{-1} chloroform solution versus 0.1M (ACN) $TBAPF_6$ solution measured at 50 mV·s^{-1}, 5 mV step

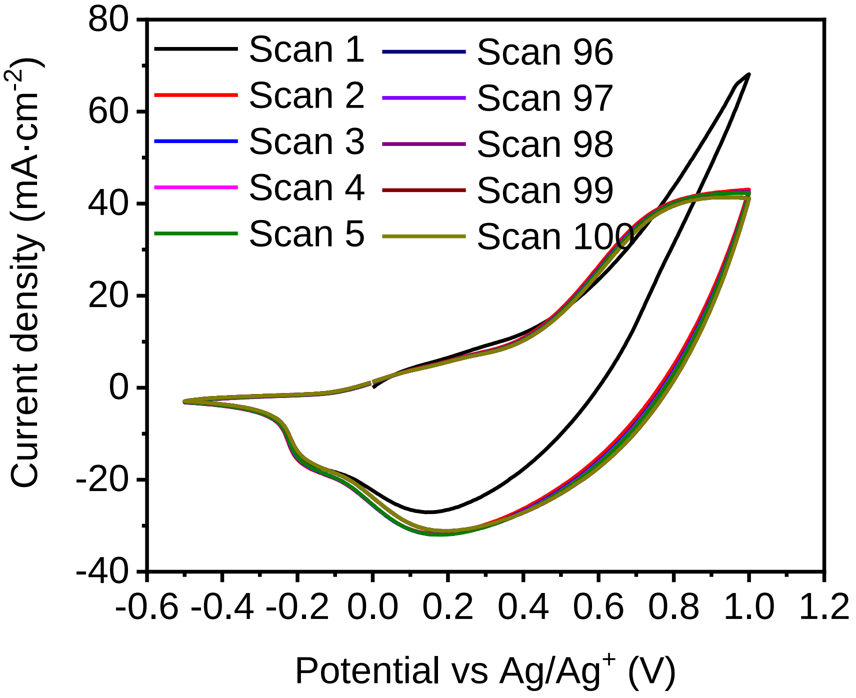

Figure S60: Cyclic voltammetry of the thin film of **inDTP-T** drop-cast from 5mg·ml^{-1} chloroform solution versus 0.1M (ACN) $TBAPF_6$ solution measured at 300 mV·s^{-1}, 5 mV step

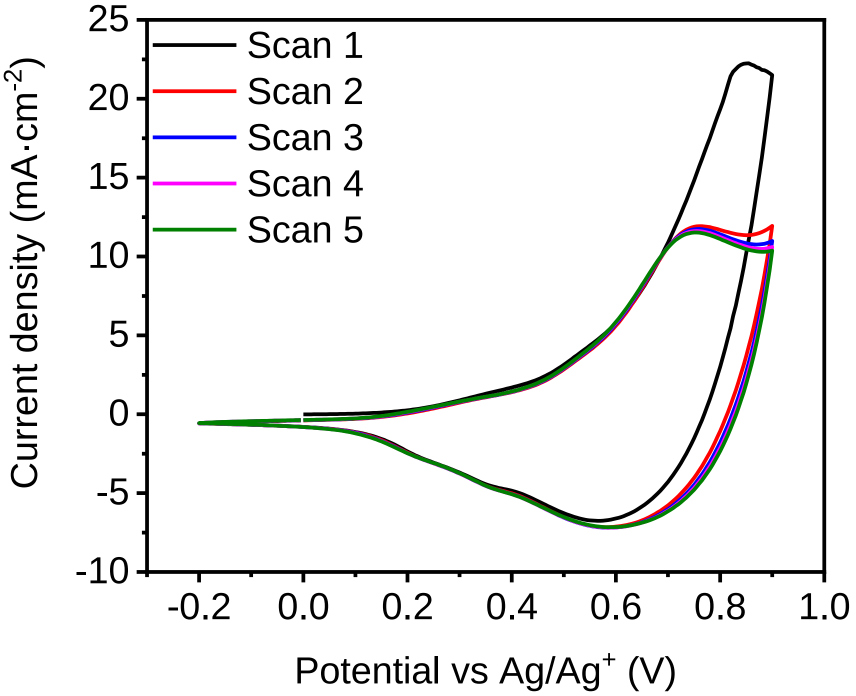

Figure S61: Cyclic voltammetry of the thin film of **inDTP-T** drop-cast from 5mg·ml^{-1} chloroform solution versus 0.1M (aq) NaCl solution measured at 50 mV·s^{-1}, 5 mV step

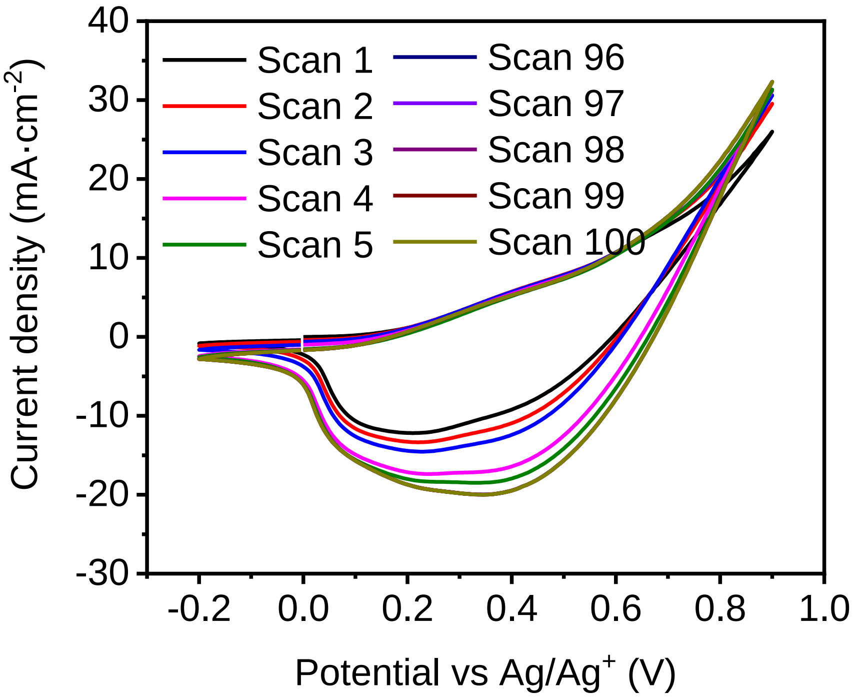

Figure S62: Cyclic voltammetry of the thin film of **inDTP-T** drop-cast from 5mg·ml^{-1} chloroform solution versus 0.1M (aq) NaCl solution measured at 300 mV·s^{-1}, 5 mV step

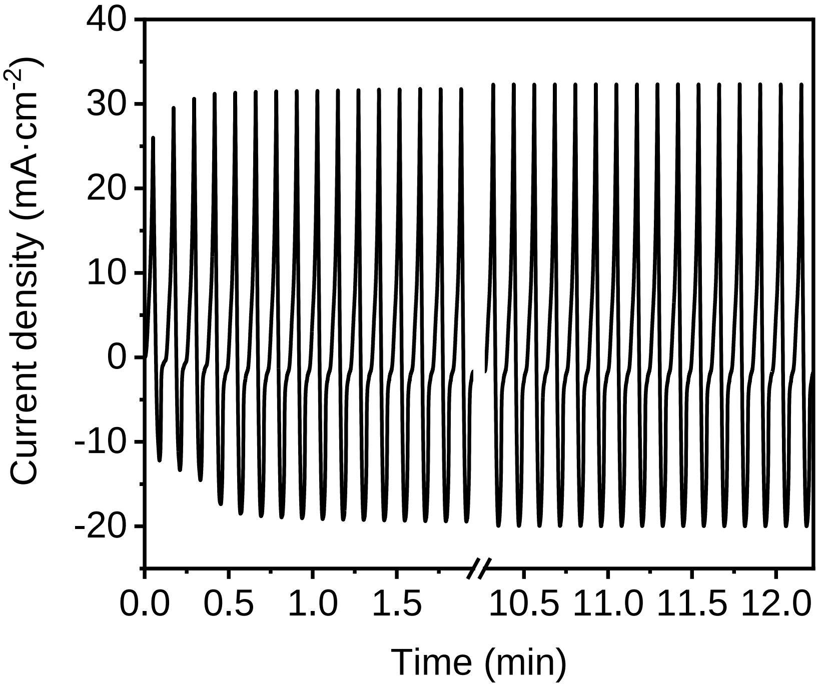

Figure S63: Stability measurement of **inDTP-T** using cyclic voltammetry of thin polymer film on the glassy carbon electrode drop-cast from chloroform solution (5 mg·ml^{-1}) with a scan rate of 300 mV·s^{-1} with 0.1 M NaCl as supporting electrolyte in water (100 scans), measured in the range of -0.2 – 0.9 V

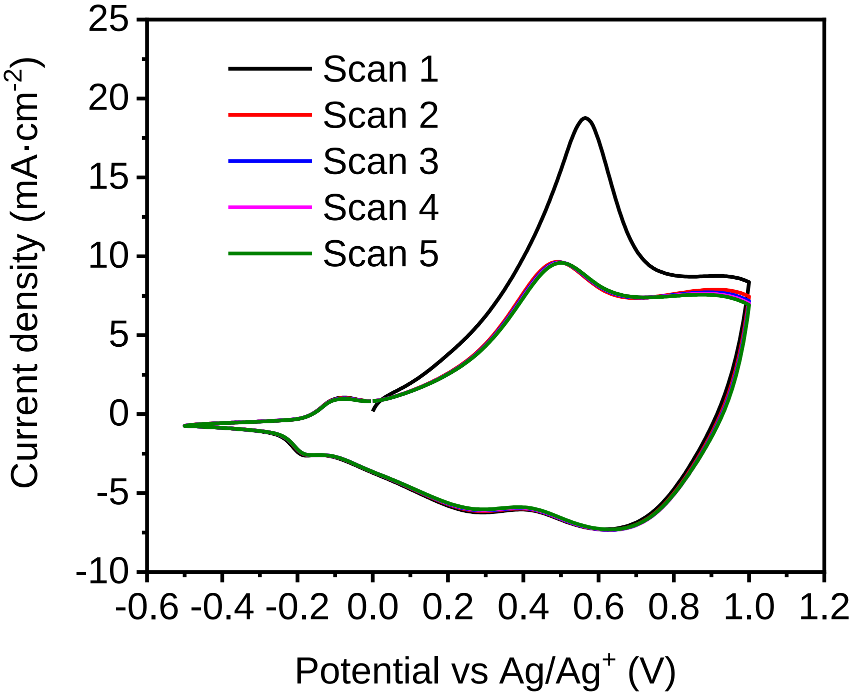

Figure S64: Cyclic voltammetry of the thin film of **outDTP-T** drop-cast from 5mg·ml^{-1} chloroform solution versus 0.1M (ACN) $TBAPF_6$ solution measured at 50 mV·s^{-1}, 5 mV step

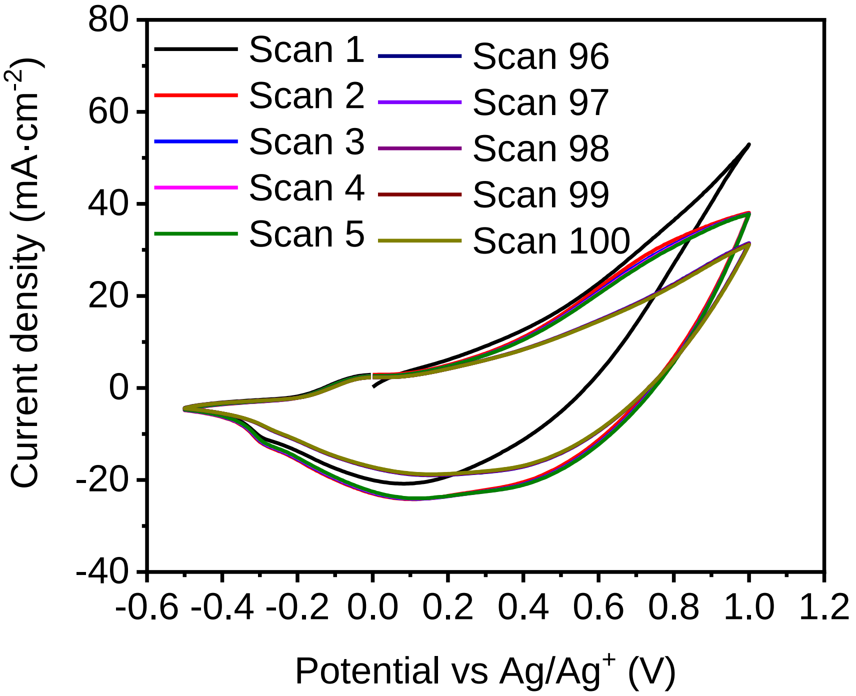

Figure S65: Cyclic voltammetry of the thin film of **outDTP-T** drop-cast from 5mg·ml^{-1} chloroform solution versus 0.1M (ACN) $TBAPF_6$ solution measured at 300 mV·s^{-1}, 5 mV step

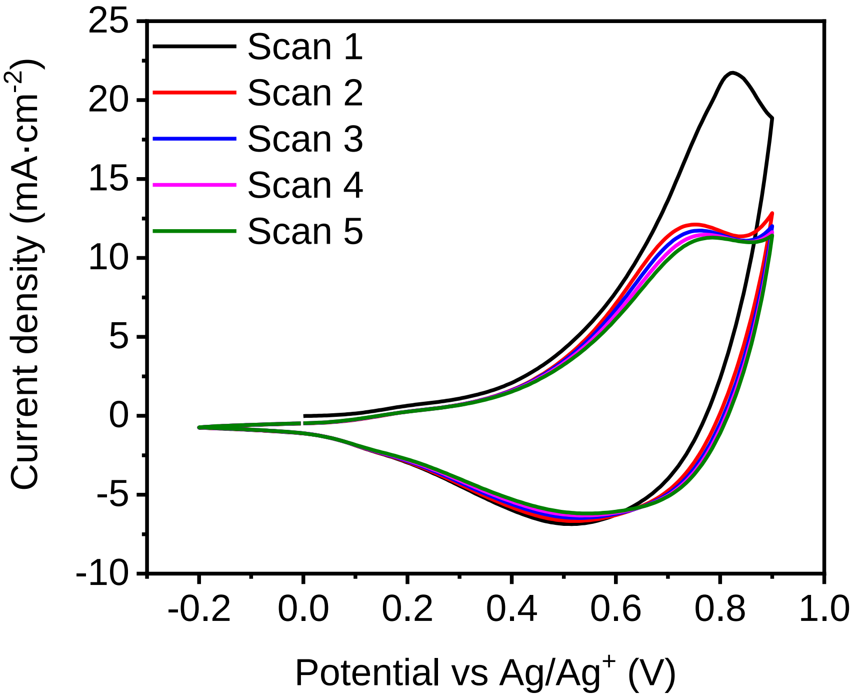

Figure S66: Cyclic voltammetry of the thin film of **outDTP-T** drop-cast from 5mg·ml^{-1} chloroform solution versus 0.1M (aq) NaCl solution measured at 50 mV·s^{-1}, 5 mV step

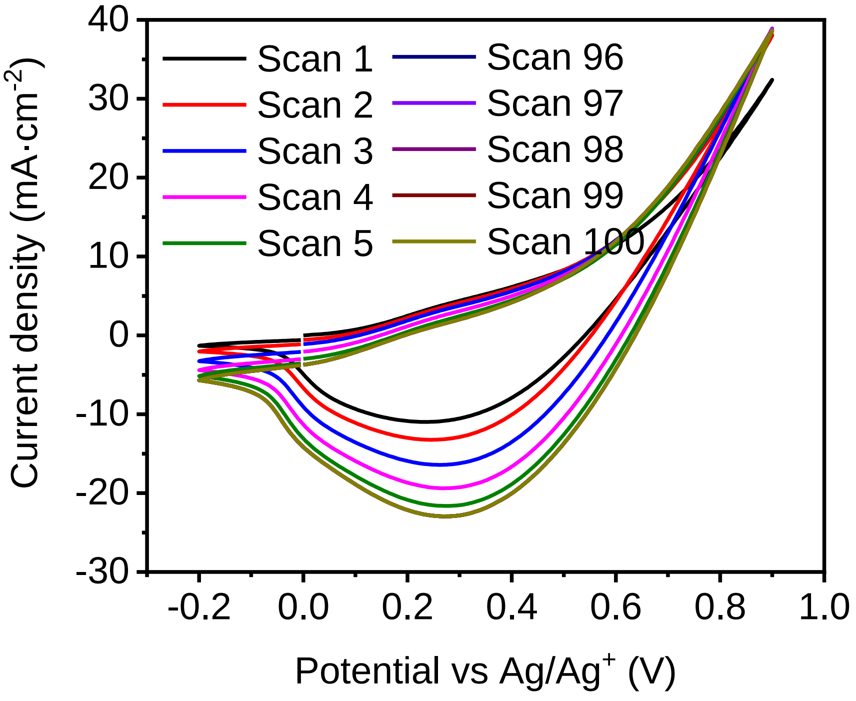

Figure S67: Cyclic voltammetry of the thin film of **outDTP-T** drop-cast from 5mg·ml^{-1} chloroform solution versus 0.1M (aq) NaCl solution measured at 300 mV·s^{-1}, 5 mV step

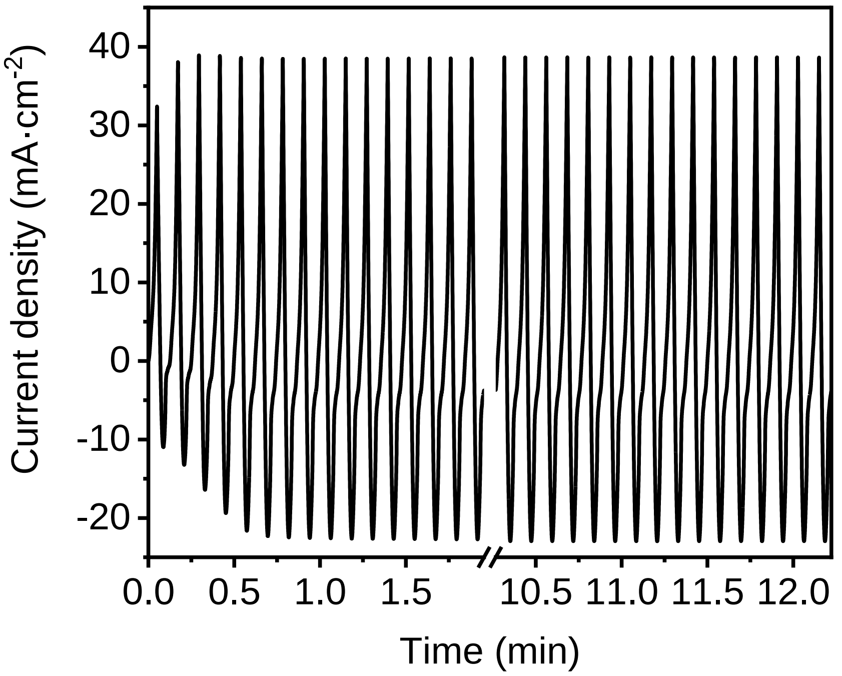

Figure S68: Stability measurement of **outDTP-T** using cyclic voltammetry of thin polymer film on the glassy carbon electrode drop-cast from chloroform solution (5 mg·ml^{-1}) with a scan rate of 300 mV·s^{-1} with 0.1 M NaCl as supporting electrolyte in water (100 scans), measured in the range of -0.2 – 0.9 V

8 Spectroelectrochemistry

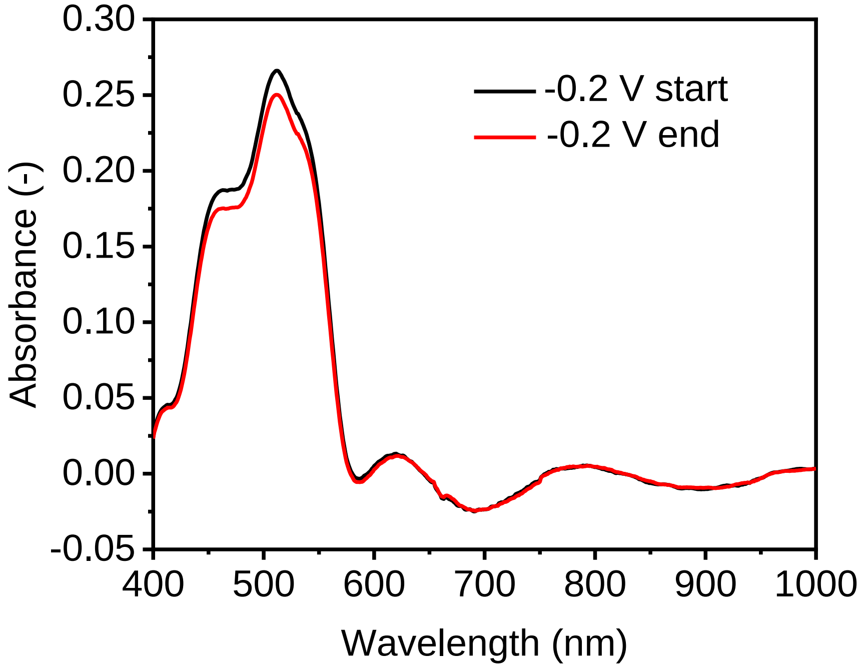

Figure S69: Spectroelectrochemistry of **inDTP-P** spin-cast from 5mg·ml^{-1} chloroform solution on ITO glass slides in 0.1M NaCl water solution (UV-vis of the beginning and the end of the cycle)

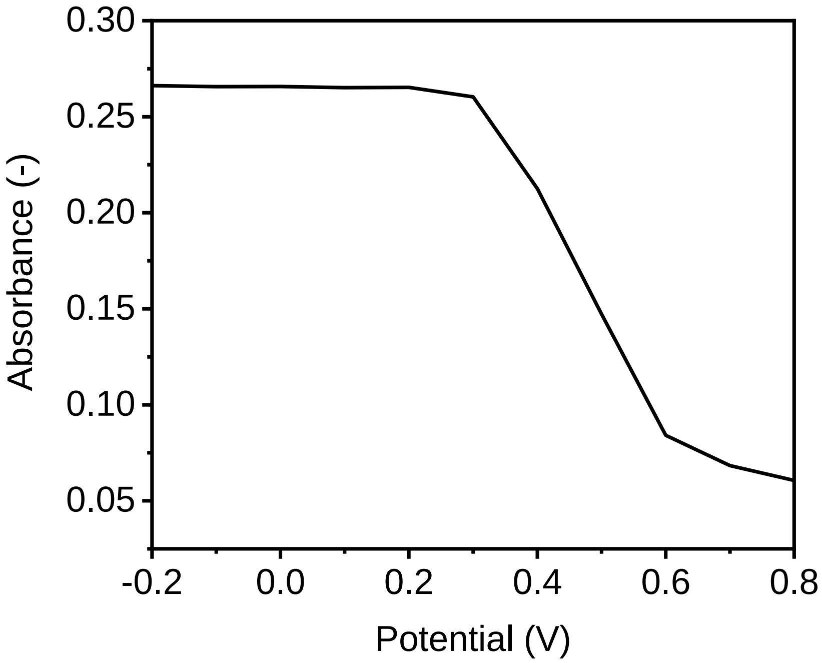

Figure S70: Spectroelectrochemistry of **inDTP-P** spin-cast from 5mg·ml^{-1} chloroform solution on ITO glass slides in 0.1M NaCl water solution plotted as dependency of absorption at 512 nm on applied bias

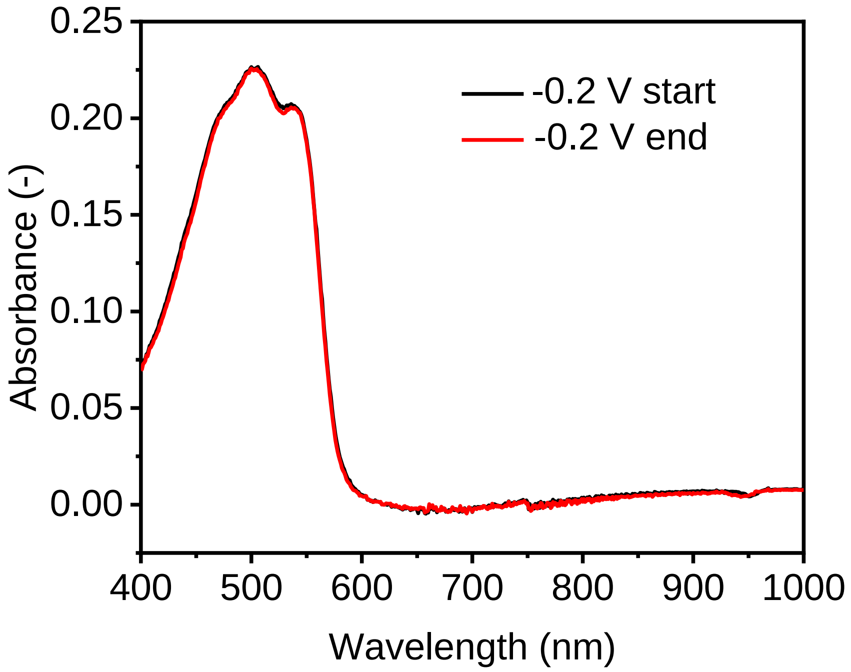

Figure S71: Spectroelectrochemistry of **outDTP-P** spin-cast from 5mg·ml^{-1} chloroform solution on ITO glass slides in 0.1M NaCl water solution (UV-vis of the beginning and the end of the cycle)

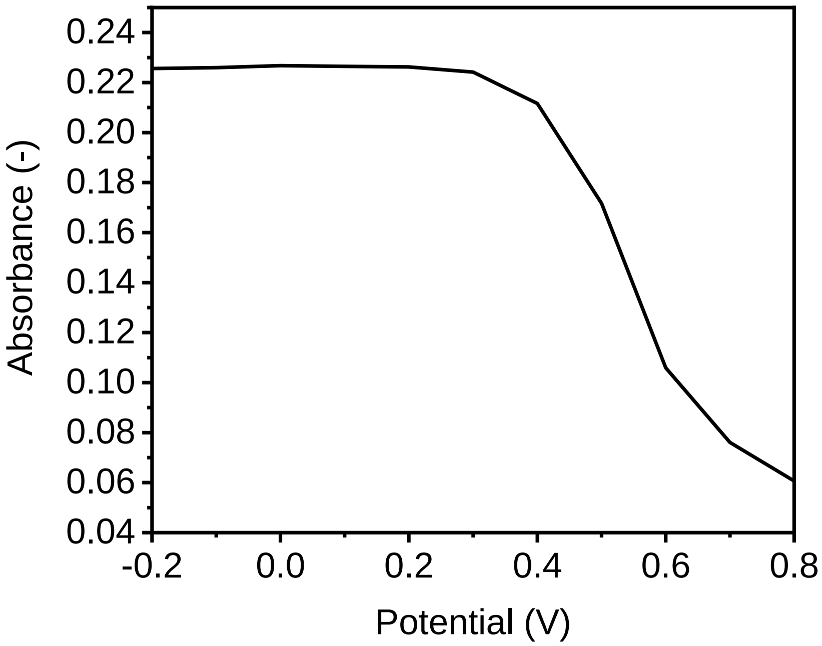

Figure S72: Spectroelectrochemistry of **outDTP-P** spin-cast from 5mg·ml^{-1} chloroform solution on ITO glass slides in 0.1M NaCl water solution plotted as dependency of absorption at 503 nm on applied bias

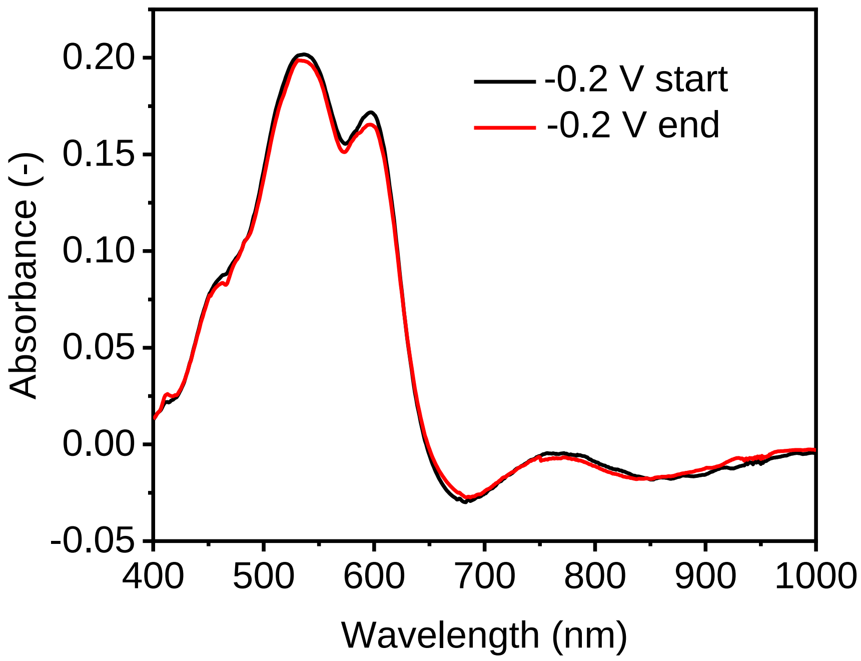

Figure S73: Spectroelectrochemistry of **inDTP-T** spin-cast from 5mg·ml^{-1} chloroform solution on ITO glass slides in 0.1M NaCl water solution (UV-vis of the beginning and the end of the cycle)

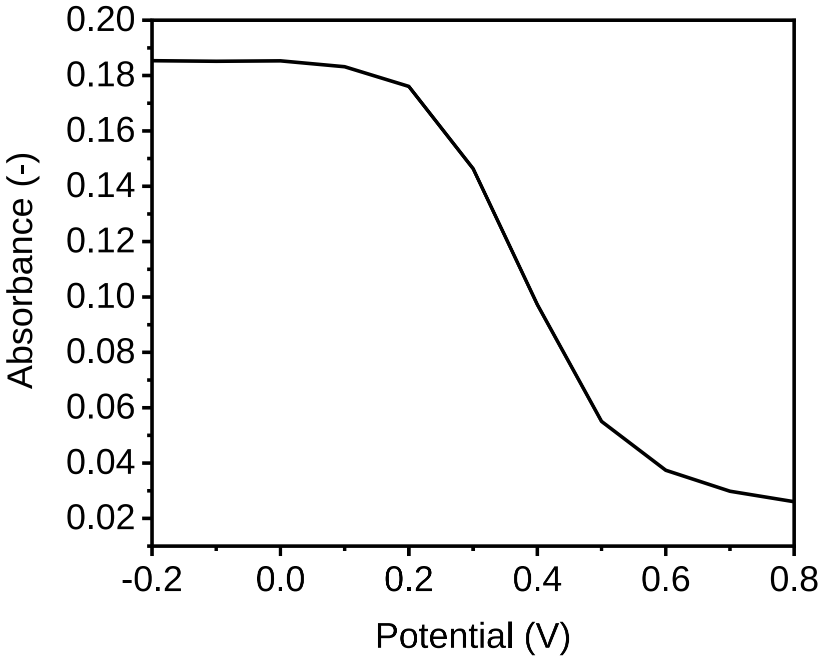

Figure S74: Spectroelectrochemistry of **inDTP-T** spin-cast from 5mg·ml^{-1} chloroform solution on ITO glass slides in 0.1M NaCl water solution plotted as dependency of absorption at 555 nm on applied bias

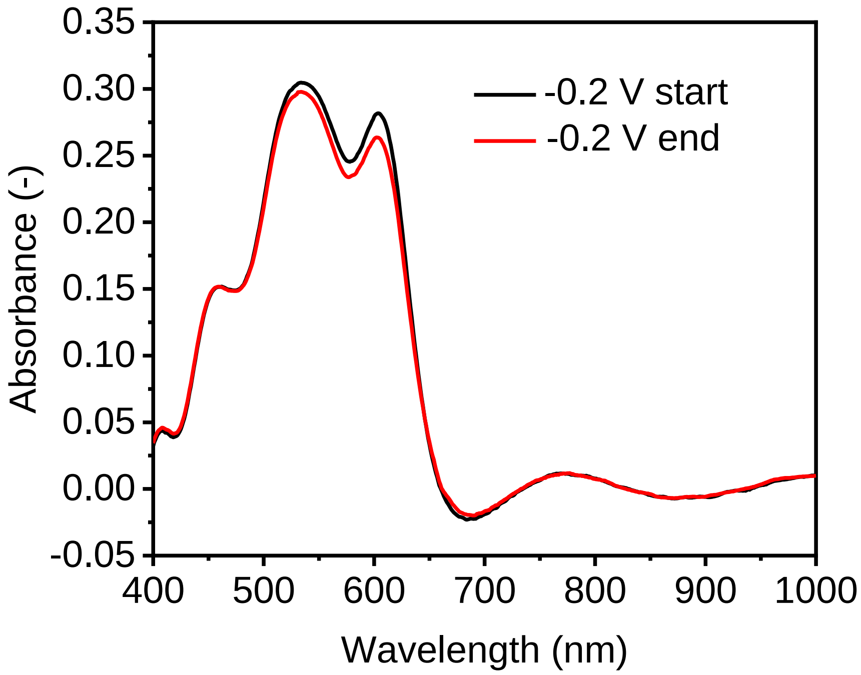

Figure S75: Spectroelectrochemistry of **outDTP-T** spin-cast from 5mg·ml^{-1} chloroform solution on ITO glass slides in 0.1M NaCl water solution (UV-vis of the beginning and the end of the cycle)

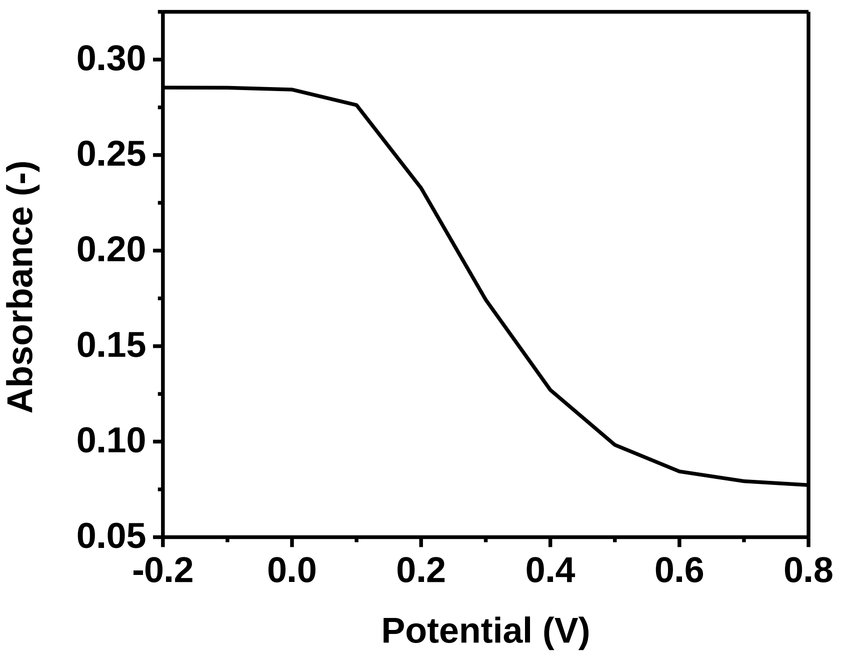

Figure S76: Spectroelectrochemistry of **outDTP-T** spin-cast from 5mg·ml^{-1} chloroform solution on ITO glass slides in 0.1M NaCl water solution plotted as dependency of absorption at 555 nm on applied bias

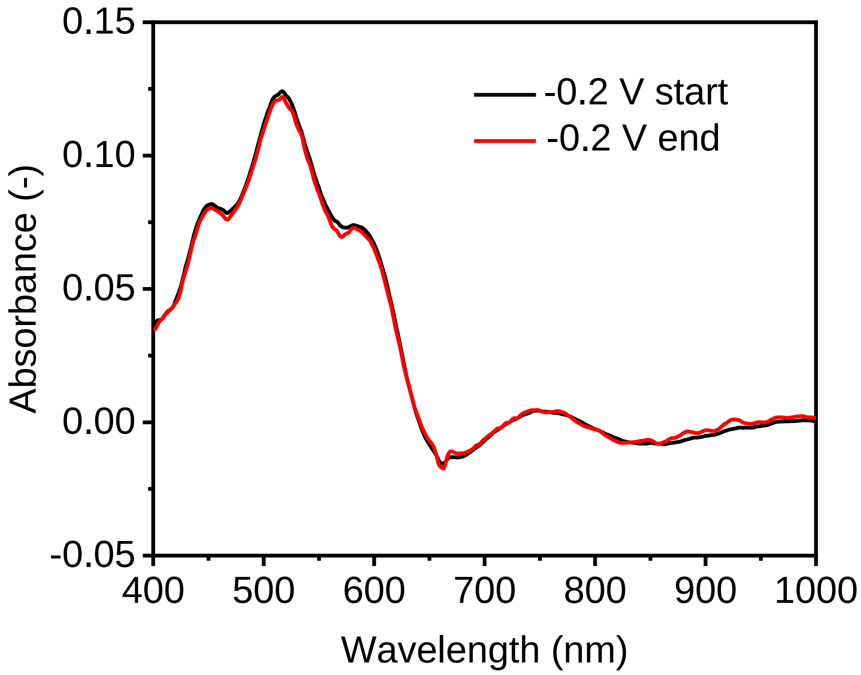

Figure S77: Spectroelectrochemistry of **inDTP-2T** spin-cast from 5mg·ml^{-1} chloroform solution on ITO glass slides in 0.1M NaCl water solution (UV-vis of the beginning and the end of the cycle)

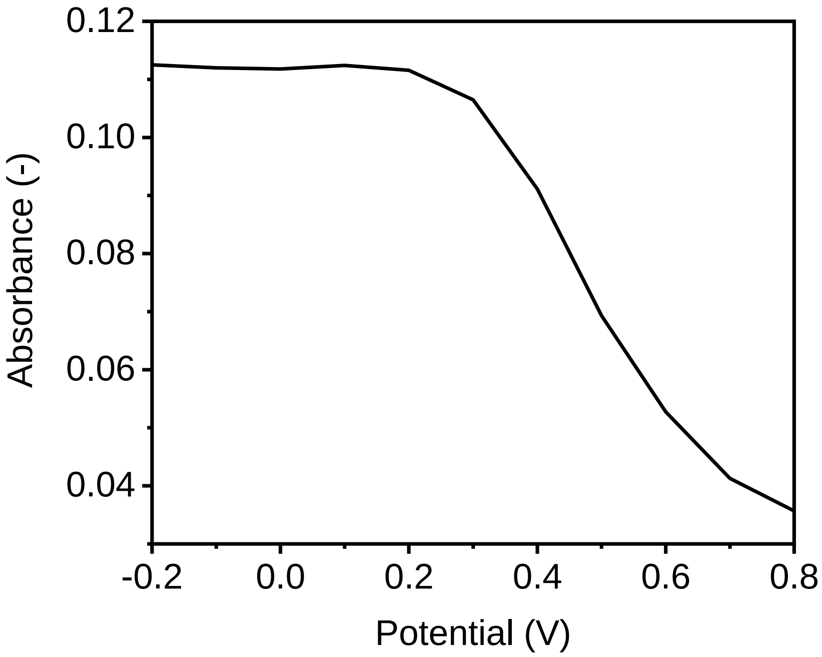

Figure S78: Spectroelectrochemistry of **inDTP-2T** spin-cast from 5mg·ml^{-1} chloroform solution on ITO glass slides in 0.1M NaCl water solution plotted as dependency of absorption at 531 nm on applied bias

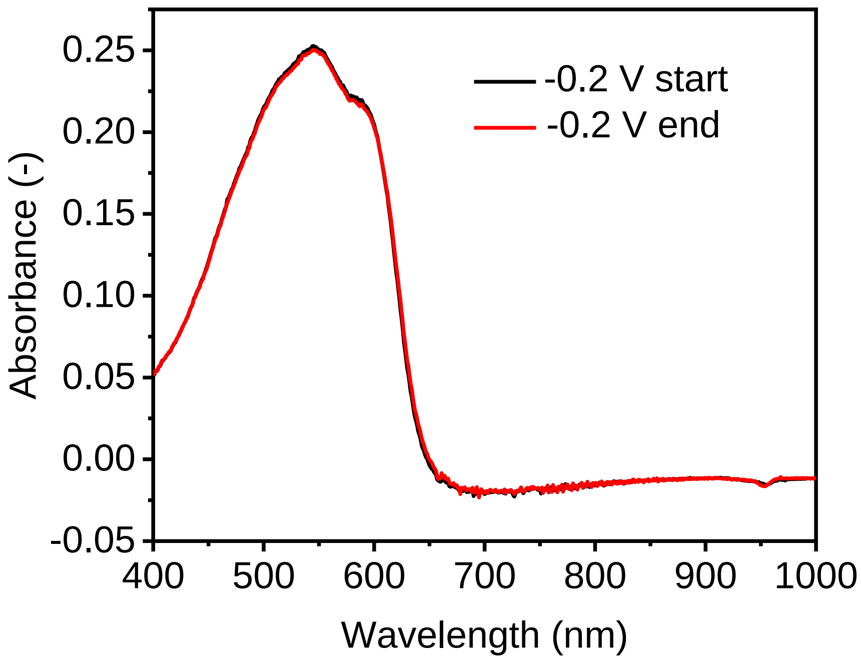

Figure S79: Spectroelectrochemistry of **outDTP-2T** spin-cast from 5mg·ml^{-1} chloroform solution on ITO glass slides in 0.1M NaCl water solution (UV-vis of the beginning and the end of the cycle)

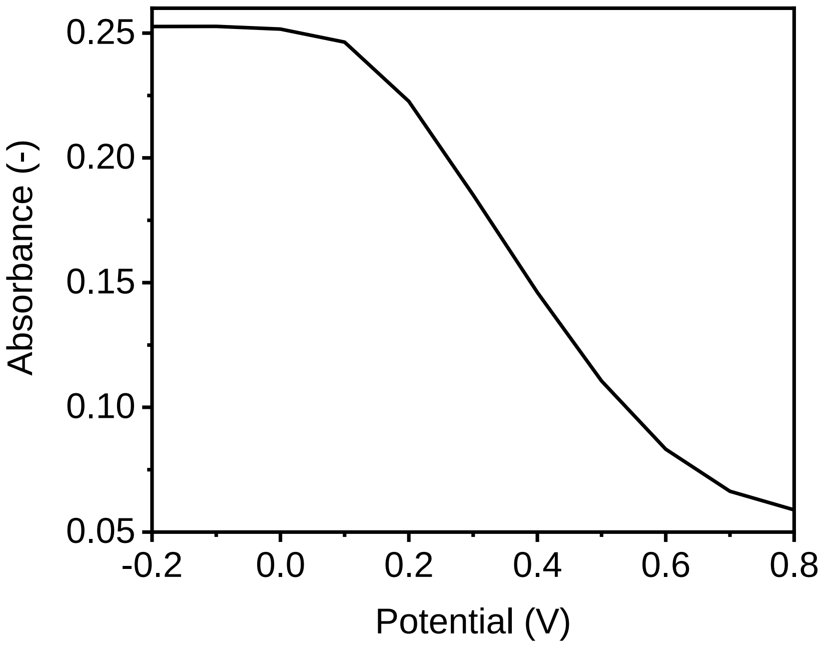

Figure S80: Spectroelectrochemistry of **outDTP-2T** spin-cast from 5mg·ml^{-1} chloroform solution on ITO glass slides in 0.1M NaCl water solution plotted as dependency of absorption at 545 nm on applied bias

9 OECT fabrication

Device fabrication procedure is as follows. After sequential ultrasonic cleaning of p-Si++/SiO2 (300 nm) substrates by placing them in a bath of acetone and isopropanol for 5 min each, source and drain contact electrodes [thermally evaporated Cr (5 nm)/Au (40 nm)] were defined via conventional photolithography. Active polymer films were fabricated by spin-casting on the substrate with patterned contact electrode. To protect the polymer film during patterning process, CYTOP thin film was fabricated on the active polymer layer by spin-casting. Prior to the photolithography process, O2 plasma treatment (5 sccm, 100 W, 10 s) was conducted to improve the adhesion of photoresist to the CYTOP layer. The positive photoresist (GXR-601, Microchemical GmbH) pattern was prepared on the active polymer layer, and the non-channel area was removed by dry-etching. After the residual photoresist removal, SU-8 photoresist (Microchemicals GmbH) was used to passivate the outer side of electrode from the electrolyte. Finally, CYTOP layer is removed by dipping the devices into fluorosolvent (Novec 7300, 3M) for 3 h with stirring. The channel width was 80 μm defined by positive photoresist pattern, and the length of channel was varied from 20 μm to 80 μm by the contact electrode pattern. All devices were measured under N2 condition using two Keithley 2400 source meters controlled by Matlab software, while an Ag/AgCl electrode and the solution of 0.1 M NaCl were employed as non-polarizable gate electrode and electrolyte, respectively.

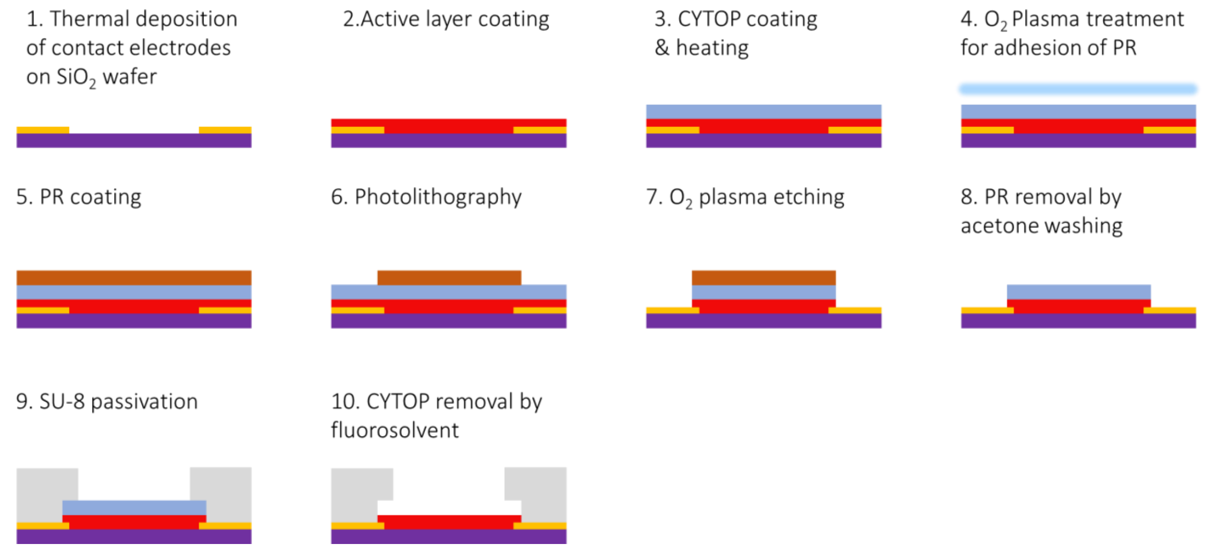

Figure S81: The process of fabrication of organic electrochemical transistors

10 OECT performance

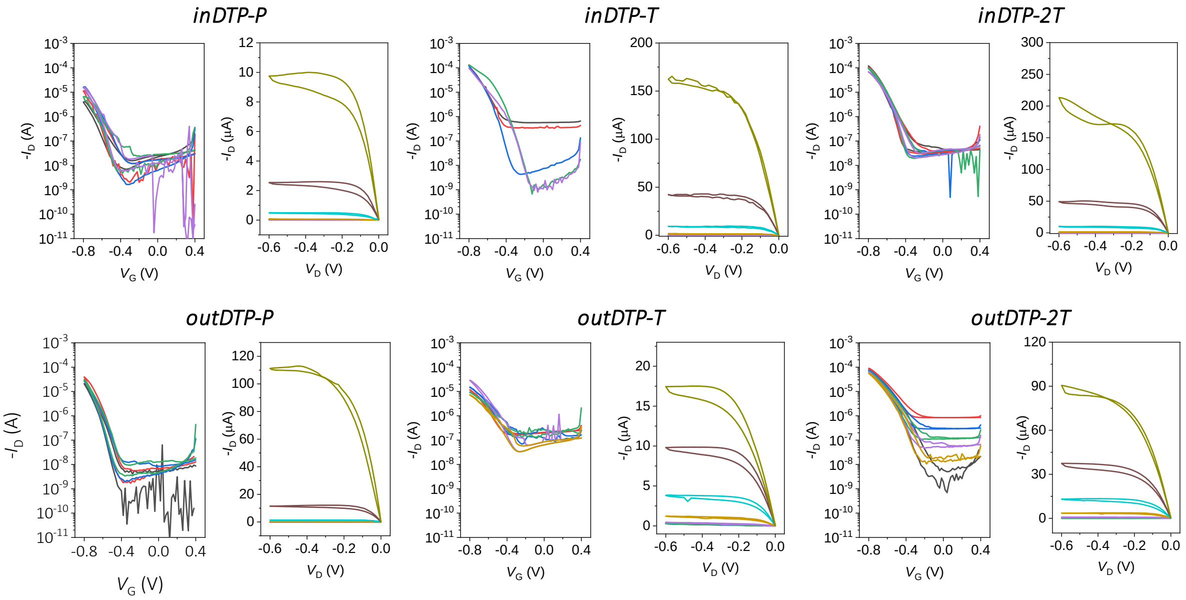

Figure S82: Representative transfer and output characteristics of OECT devices for all six polymers

11 Electrochemical impedance spectroscopy

For EIS measurements, all polymer films were prepared on indium tin oxide (ITO)-coated glass substrates as working electrodes, while the electrode surface other than the active electrode area was passivated using epoxy glue and in contact with an electrolyte solution of 0.1 M NaCl in water. Electrochemical impedance spectra were obtained using PGSTAT304N (Metrohm) equipped with a conventional three-electrode system composed of a working electrode, an Ag/AgCl reference electrode, and a Pt counter electrode, at the frequency range between 0.1 and 105 Hz with a single sinusoidal signal of Eac = 10 mV.

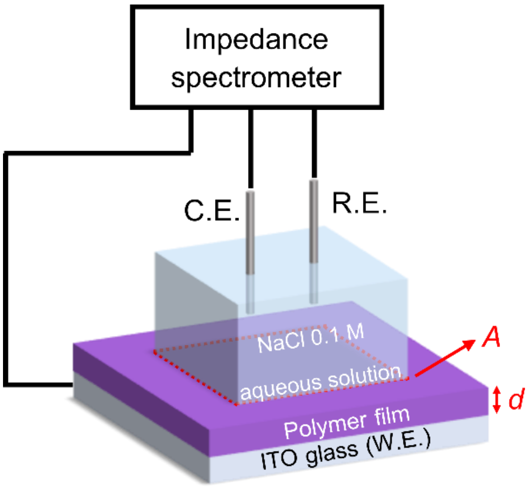

Figure S83: Electrochemical impedance spectroscopy set-up

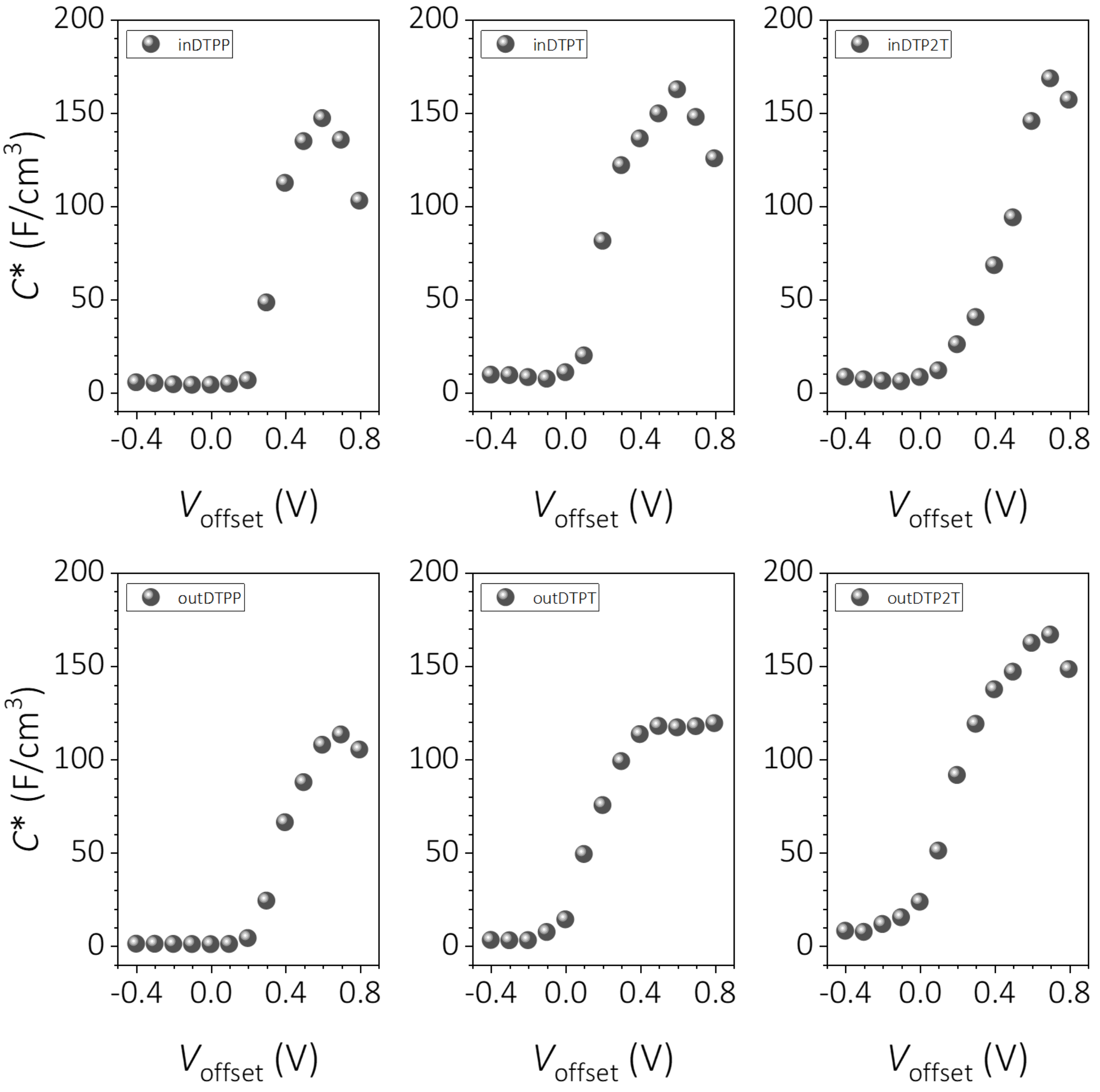

Figure S84: Electrochemical impedance spectroscopy of all six polymers

12 Density Functional Theory calculations

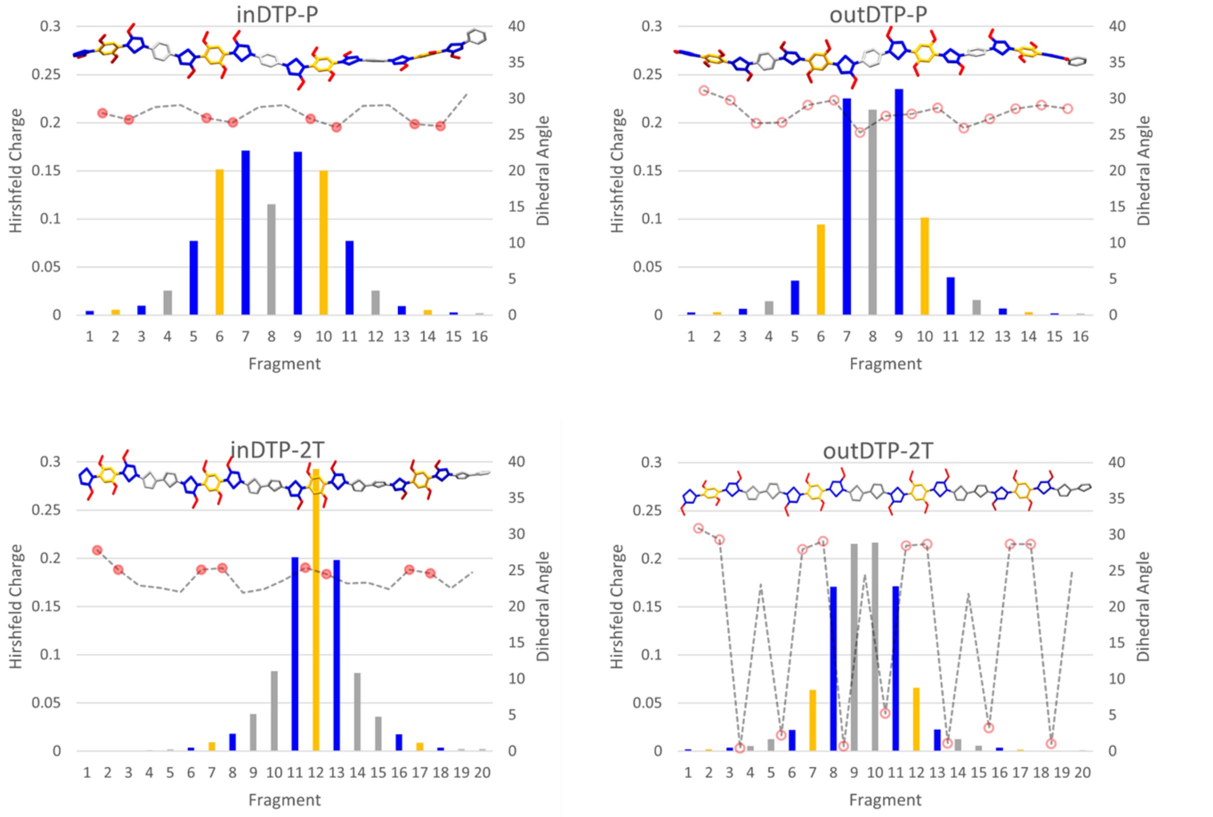

Figure S85: Hirshfeld charge distribution (left axis and bar chart) by fragment (blue = glycolated thiophene; orange = glycolated phenylene; grey = comonomer unit) and dihedral angles along optimised polymer chains (right axis, line plot) with two intramolecular O-X interactions supporting the dihedral configuration denoted by filled circles, one O-X interaction denoted by open circles and no O-X interactions denoted by no circle

13 Grazing-Incidence Wide-Angle X-ray Scattering

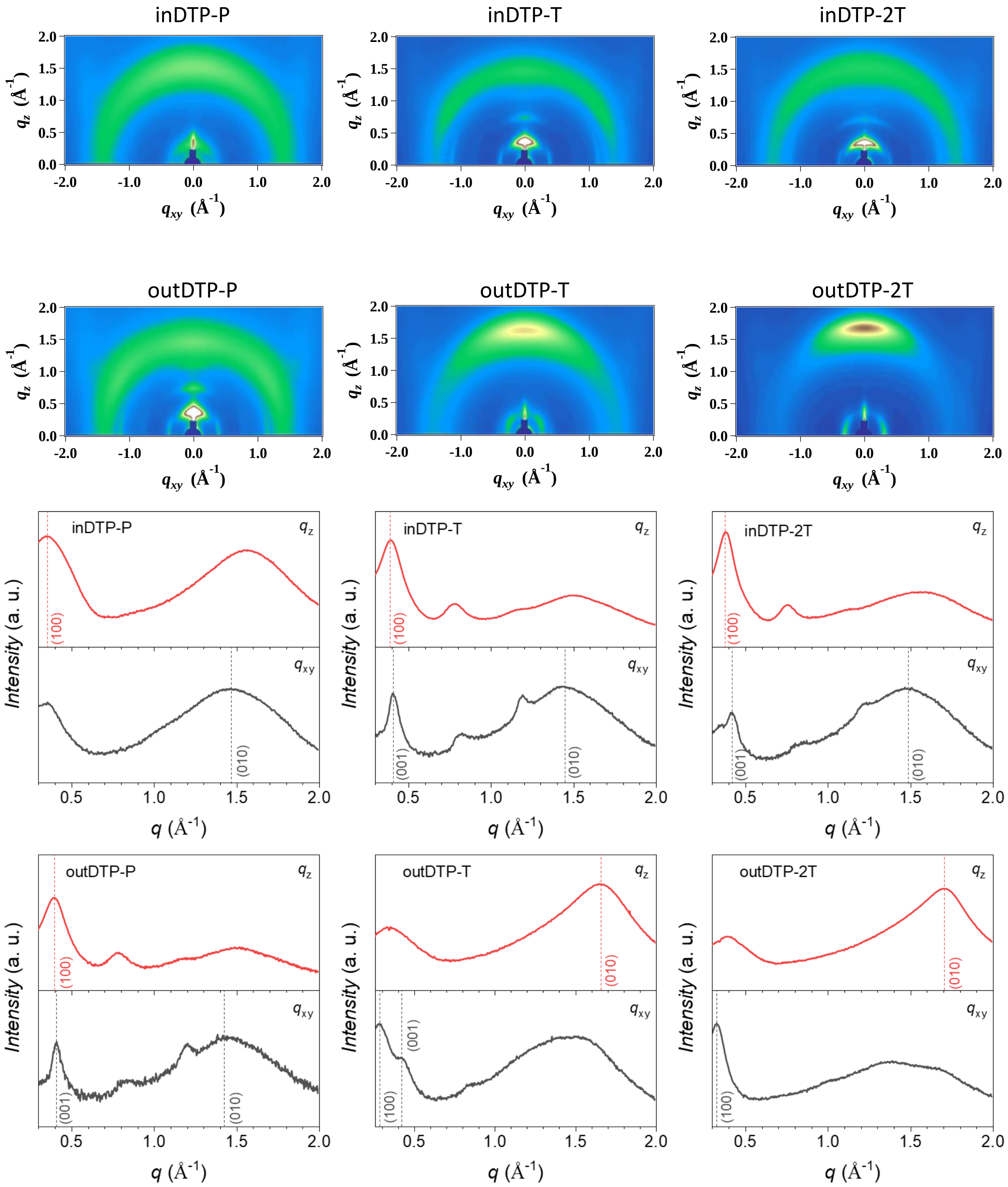

Figure S86: 2D GIWAXS patterns (top) and corresponding line cuts (bottom) of all polymers obtained from thin films spin-cast from chloroform (5 mg.ml^{-1}) onto ITO-coated glass. The X-ray wavelength was 1.11794 Å (E = 11.09 keV), and the incidence angle of the beam light was ~0.1°. The sample-to-detector distance was adjusted to be 220 mm

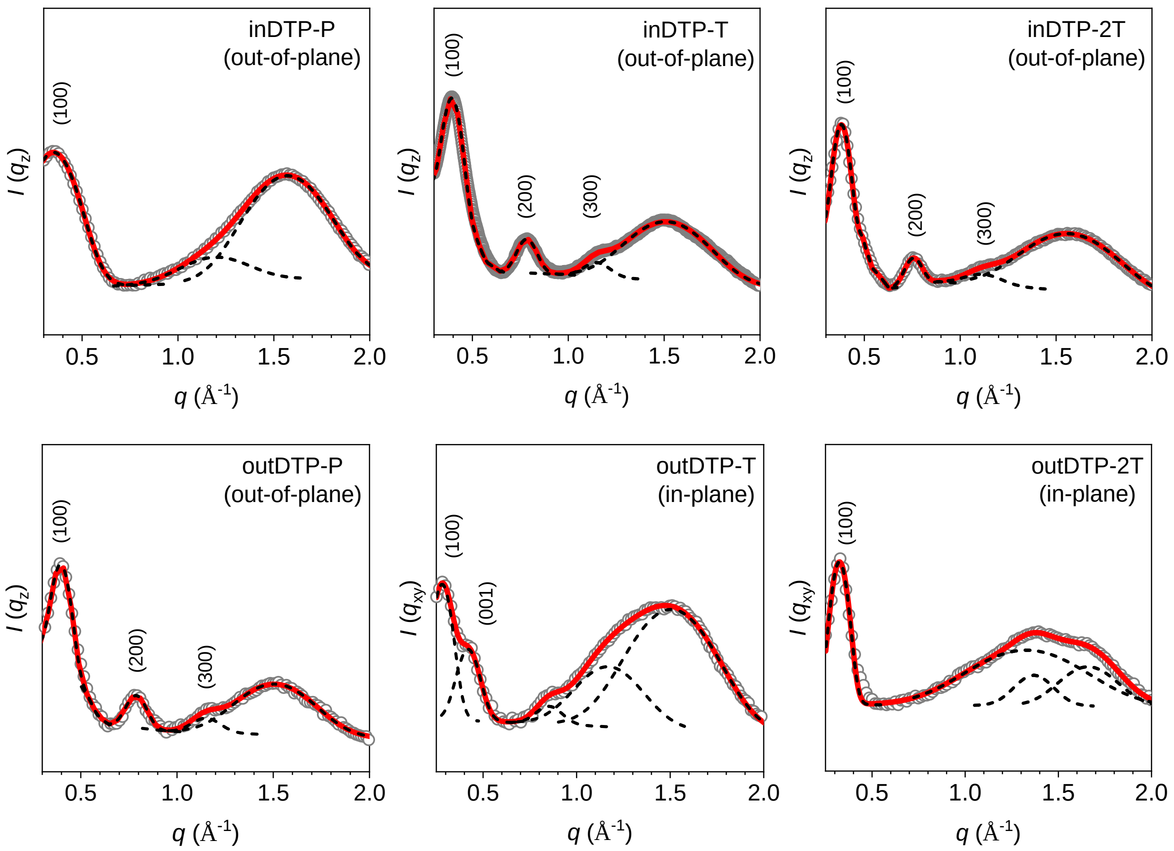

Figure S87: Fittings for GIWAXS peak coherence length calculations using the equation $L_c = 2\pi K/\Delta_q$ where K is the shape factor (typically 0.8-1) and delta q is the full width at half-maximum (FWHM) of a diffraction peak. However, care must be taken when using the Scherrer formula as it assumes that only crystalline size contributes to peak width and ignores disorder

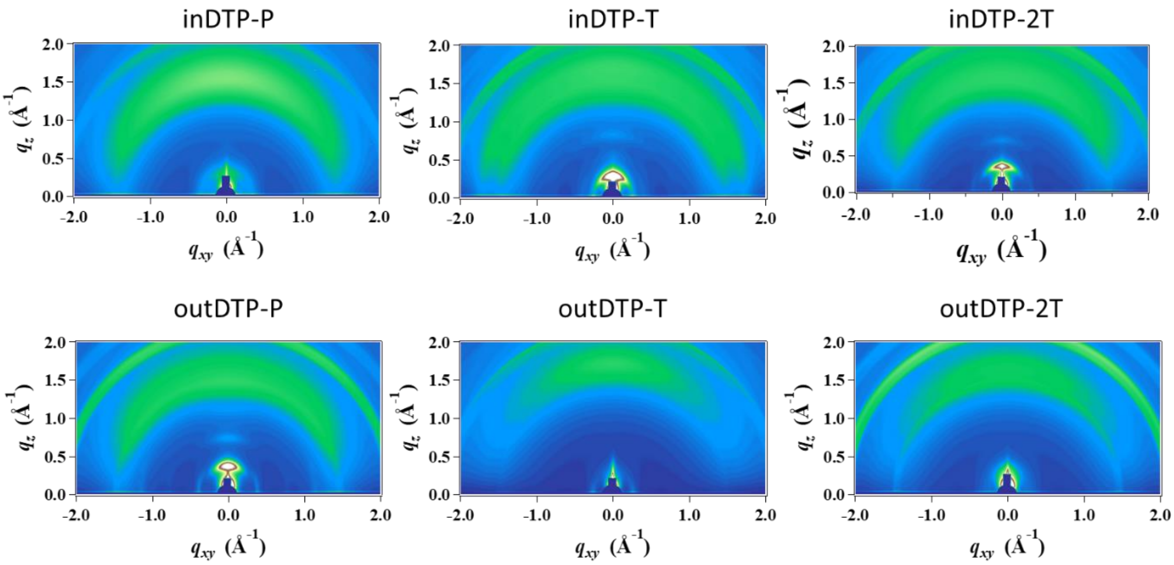

Figure S88: 2D GIWAXS patterns of all oxidized polymers obtained from thin films spin-cast from chloroform (5 mg.ml^{-1}) onto ITO-coated glass. The X-ray wavelength was 1.11794 Å (E = 11.09 keV), and the incidence angle of the beam light was ~0.1°. The sample-to-detector distance was adjusted to be 220 mm

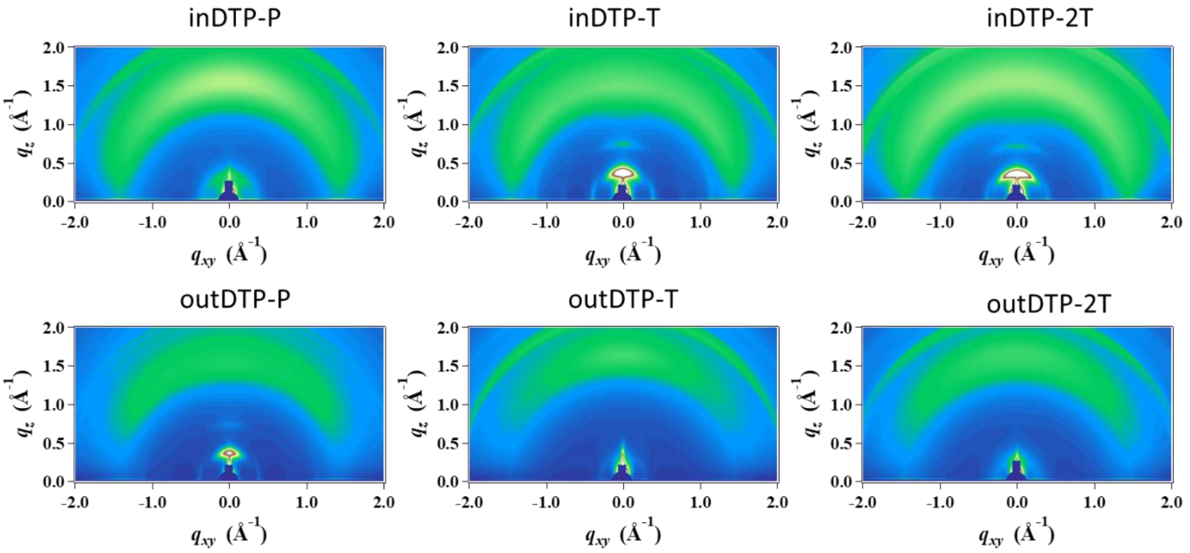

Figure S89: 2D GIWAXS patterns of all restored polymers (reduced back to neutral) obtained from thin films spin-cast from chloroform (5 mg.ml^{-1}) onto ITO-coated glass. The X-ray wavelength was 1.11794 Å (E = 11.09 keV), and the incidence angle of the beam light was ~0.1°. The sample-to-detector distance was adjusted to be 220 mm

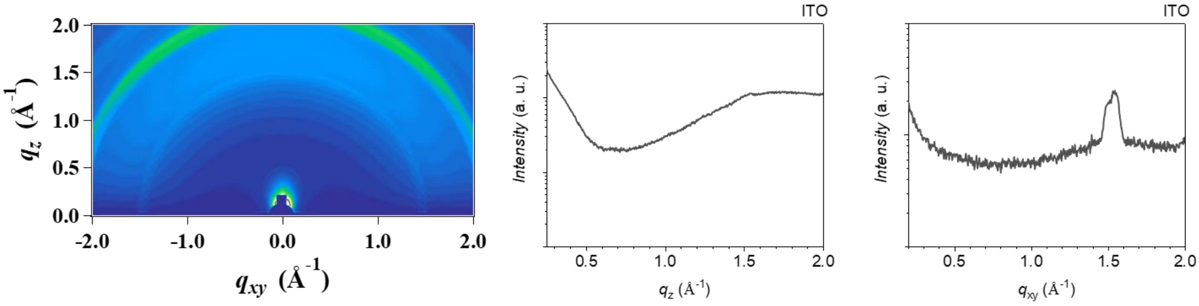

Figure S90: 2D GIWAXS pattern of ITO-coated glass as a reference sample. The X-ray wavelength was 1.11794 Å (E = 11.09 keV), and the incidence angle of the beam light was ~0.1°. The sample-to-detector distance was adjusted to be 220 mm

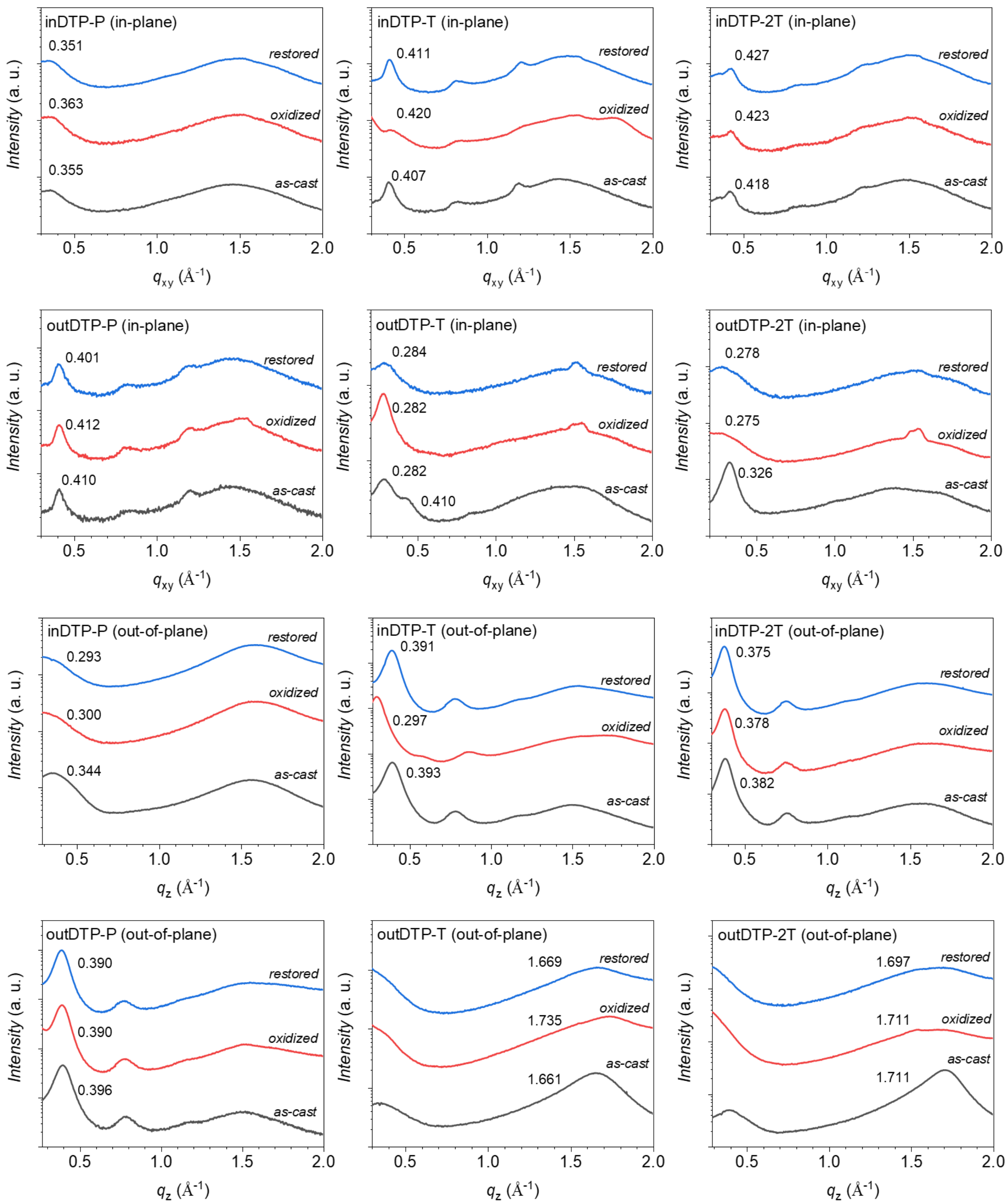

Figure S91: GIWAXS line cuts of all as-cast, oxidized, and restored polymers (reduced back to neutral) obtained from thin films spin-cast from chloroform (5 mg.ml^{-1}) onto ITO-coated glass. The X-ray wavelength was 1.11794 Å (E = 11.09 keV), and the incidence angle of the beam light was ~0.1°. The sample-to-detector distance was adjusted to be 220 mm

14 Electrochemical quartz crystal microbalance

We performed electrochemical quartz crystal microbalance with dissipation monitoring (EQCM-D) measurements using a Q-sense analyzer (QE401, Biolin Scientific). Swelling measurements were performed as follows. First, we recorded the QCM-D response of the bare Au sensors in the air, followed by the injection of the $NaCl_{(aq.)}$ 0.1 M solutions into the chamber. This resulted in large shifts in frequency (*f*) and dissipation of energy (*D*), due to the density differences between the two media. The measurements were then stopped, the sensors were removed, and polymer films were spin-cast directly on the same sensor from a 5 mg/mL chloroform solution at 1000 rpm. The absolute *f* value for each polymer coated sensor was obtained both in air and in $NaCl_{(aq.)}$ 0.1 M, after the *f* signal was perfectly flat (i.e., f <0.5 Hz) assuring that the system is in equilibrium. We then compared the absolute difference in *f* for multiple overtones between the bare sensor and the polymer coated sensors, both in air and in $NaCl_{(aq.)}$ 0.1 M by using the function "stitched data" of Q-soft software. This function compares the selected datasets based on the raw frequencies measured and excludes the effect of the different densities between the two media (Figure S92, Table S4). Thus, the difference of the *f* values of the stitched data is directly analogous to the thickness of the polymer in both media, which is calculated by using the Sauerbrey equation below (eq. 1). EQCM-D measurements were performed using a PalmSens potentiostat coupled with Q-sense electrochemistry module. The three-electrode setup was comprised of Ag/AgCl reference, Pt counter and Au/polymer EQCM-D sensor as the working electrode. The physical modelling of the two measured parameters, the *f* and *D*, is related to the viscoelastic properties of the film. On the one hand, a rigid film shows zero *D* as there are theoretically no energy losses (no viscoelasticity) and Sauerbrey equation can be used to quantify the mass (*m*), using only one overtone as described in equation 1:

eq. 1: $$\Delta m = \frac{-17.7}{n} \Delta f_n$$

On the other hand, a softer film does not follow the motion of the crystal and leads to energy losses during the oscillation. We approximate soft films to behave like a Kelvin-Voigt element, which means that they exhibit both viscous and elastic characteristics acting in parallel (viscoelastic). A Kelvin-Voigt element has a complex shear modulus as described in equation 2:

eq. 2: $$G^* = \mu + 2\pi i f \eta$$

where G^* is the complex shear modulus, μ is elasticity ($kgm^{-1}s^{-2}$), η is viscosity ($kgm^{-1}s^{-1}$) and *f* is the frequency. To calculate the mass changes of a thick, viscoelastic film, complex shear modulus was analyzed and fitted using three different overtones (3rd, 5th and 7th) – Figure S93 and Table S4. Q-Tools and D-find software were used for modeling and data analysis. Since the polymer films absorb a significant amount of water under doping potentials, we used the Kelvin-Voigt viscoelastic (VSE) model to fit the data.

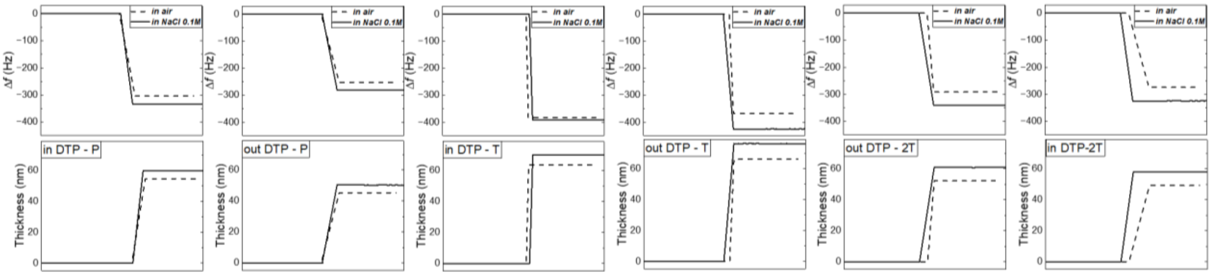

Figure S92: Frequency shifts (Δ*f*) and the corresponding thicknesses of the polymer films cast on Au coated sensors, recorded in air and when immersed in NaCl(aq.) 0.1 M. Here, we show the Δ*f* for the 5th overtone.

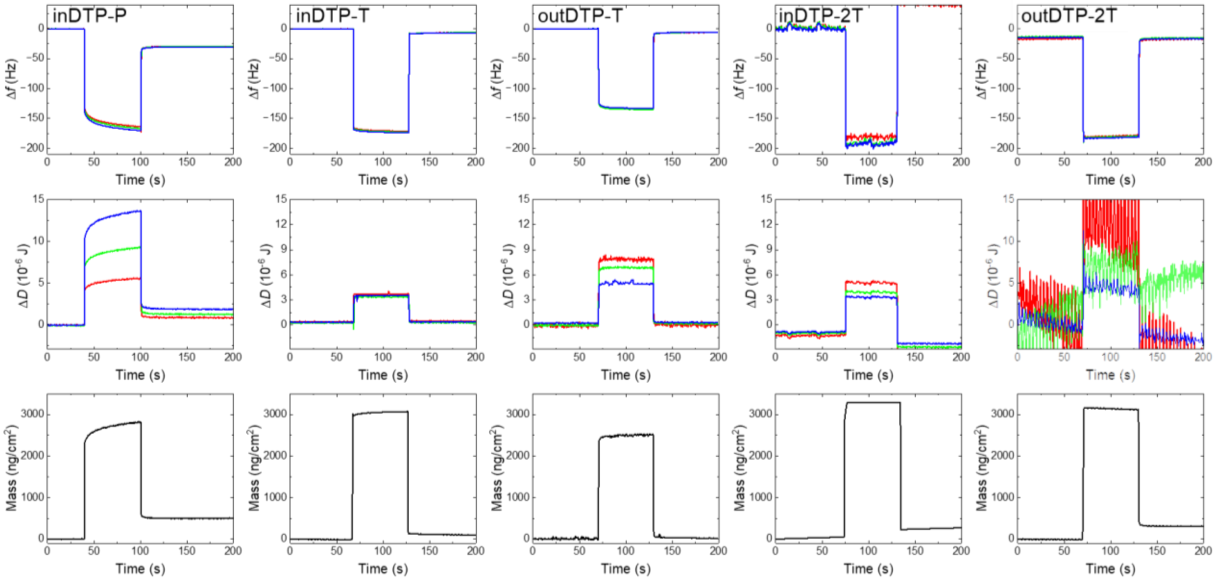

Figure S93: Frequency Shifts (Δf, top row), Dissipation of energy (ΔD, middle row), and calculated mass shifts (bottom row) for polymer films cast on Au-coated sensors, recorded in 0.1 M NaCl (aq.) when a 60 s doping pulse at V = 0.8 V vs V_{OC} is applied. The overtones shown and chosen for the analysis are the 3rd, 5th and 7th, in red, green, and blue solid lines, respectively. [*Note: data not collected for outDTP-P due to microfluidic leakages and technical issues with the QCM module.*]

Table S4: Summary of thermogravimetric analysis

Polymer	Film thickness [nm]	Passive swelling [%]	Mass at 0.8 V after 60 s [ng/cm²]
**inDTP-P**	54	11	2824
**outDTP-P**	45	13	3075
**inDTP-T**	63	12	3075
**outDTP-T**	66	16	2530
**inDTP-2T**	49	15	3292
**outDTP-2T**	52	16	3123

References

[1] C. B. Nielsen, A. Giovannitti, D. T. Sbircea, E. Bandiello, M. R. Niazi, D. A. Hanifi, M. Sessolo, A. Amassian, G. G. Malliaras, J. Rivnay, et al., *J. Am. Chem. Soc.* **2016**, *138*, 10252–10259.

[2] Z. S. Parr, R. Halaksa, P. A. Finn, R. B. Rashid, A. Kovalenko, M. Weiter, J. Rivnay, J. Krajčovič, C. B. Nielsen, *Chempluschem* **2019**, *84*, 1384–1390.
